# Cross-reactive CD4^+^ T cells enhance SARS-CoV-2 immune responses upon infection and vaccination

**DOI:** 10.1126/science.abh1823

**Published:** 2021-08-31

**Authors:** Lucie Loyal, Julian Braun, Larissa Henze, Beate Kruse, Manuela Dingeldey, Ulf Reimer, Florian Kern, Tatjana Schwarz, Maike Mangold, Clara Unger, Friederike Dörfler, Shirin Kadler, Jennifer Rosowski, Kübrah Gürcan, Zehra Uyar-Aydin, Marco Frentsch, Florian Kurth, Karsten Schnatbaum, Maren Eckey, Stefan Hippenstiel, Andreas Hocke, Marcel A. Müller, Birgit Sawitzki, Stefan Miltenyi, Friedemann Paul, Marcus A. Mall, Holger Wenschuh, Sebastian Voigt, Christian Drosten, Roland Lauster, Nils Lachman, Leif-Erik Sander, Victor M. Corman, Jobst Röhmel, Lil Meyer-Arndt, Andreas Thiel, Claudia Giesecke-Thiel

**Affiliations:** 1 Si-M/“Der Simulierte Mensch,” a Science Framework of Technische Universität Berlin and Charité – Universitätsmedizin Berlin, Berlin, Germany.; 2 Charité – Universitätsmedizin Berlin, Corporate Member of Freie Universität Berlin, Humboldt – Universität zu Berlin, and Berlin Institute of Health, Berlin, Germany.; 3 JPT Peptide Technologies GmbH, Berlin, Germany.; 4 Department of Clinical and Experimental Medicine, Brighton and Sussex Medical School, Brighton, UK.; 5 Institute of Virology, Charité – Universitätsmedizin Berlin, Berlin, Germany.; 6 Medical Biotechnology, Institute for Biotechnology, Technische Universität Berlin, Berlin, Germany.; 7 Department of Hematology, Oncology and Tumor Immunology, Charité – Universitätsmedizin Berlin, Berlin, Germany.; 8 Therapy-Induced Remodeling in Immuno-Oncology, Berlin Institute of Health at Charité – Universitätsmedizin Berlin, Berlin, Germany.; 9 Department of Infectious Diseases and Respiratory Medicine, Charité – Universitätsmedizin Berlin, Berlin, Germany.; 10 Department of Tropical Medicine, Bernhard Nocht Institute for Tropical Medicine, and Department of Medicine, University Medical Center Hamburg-Eppendorf, Hamburg, Germany.; 11 German Centre for Infection Research (DZIF), Partner Site Charité, Berlin, Germany.; 12 Institute of Medical Immunology, Charité – Universitätsmedizin Berlin, Berlin, Germany.; 13 Miltenyi Biotec GmbH, Bergisch-Gladbach, Germany.; 14 Experimental and Clinical Research Center, Max Delbrueck Center for Molecular Medicine, and Charité – Universitätsmedizin Berlin, Berlin, Germany.; 15 Clinical Neuroimmunology, NeuroCure Clinical Research Center, Charité – Universitätsmedizin Berlin, Berlin, Germany.; 16 Department of Pediatric Respiratory Medicine, Immunology and Critical Care Medicine, Charité – Universitätsmedizin Berlin, Berlin, Germany.; 17 German Center for Lung Research, Associated Partner, Berlin, Germany.; 18 Department of Infectious Diseases, Robert Koch Institute, Berlin, Germany.; 19 Institute for Virology, Universitätsklinikum Essen, Essen, Germany.; 20 Institute for Transfusion Medicine, Tissue Typing Laboratory, Charité – Universitätsmedizin Berlin, Berlin, Germany.; 21 Department of Neurology and Experimental Neurology, Charité – Universitätsmedizin Berlin, Berlin, Germany.; 22 Max Planck Institute for Molecular Genetics, Berlin, Germany.

## Abstract

The functional relevance of preexisting cross-immunity to severe acute respiratory syndrome coronavirus 2 (SARS-CoV-2) is a subject of intense debate. Here, we show that human endemic coronavirus (HCoV)–reactive and SARS-CoV-2–cross-reactive CD4^+^ T cells are ubiquitous but decrease with age. We identified a universal immunodominant coronavirus-specific spike peptide (S816-830) and demonstrate that preexisting spike- and S816-830–reactive T cells were recruited into immune responses to SARS-CoV-2 infection and their frequency correlated with anti–SARS-CoV-2-S1-IgG antibodies. Spike–cross-reactive T cells were also activated after primary BNT162b2 COVID-19 messenger RNA vaccination and displayed kinetics similar to those of secondary immune responses. Our results highlight the functional contribution of preexisting spike–cross-reactive T cells in SARS-CoV-2 infection and vaccination. Cross-reactive immunity may account for the unexpectedly rapid induction of immunity after primary SARS-CoV-2 immunization and the high rate of asymptomatic or mild COVID-19 disease courses.

## Cross-reactive T cells aid in the fight

There is mounting evidence that immunological memory after infection with seasonal human coronaviruses (hCoVs) contributes to cross-protection against severe acute respiratory syndrome coronavirus 2 (SARS-CoV-2). Loyal *et al*. identified a universal immunodominant coronavirus peptide found within the fusion peptide domain of coronavirus spike protein. This peptide is recognized by CD4^+^ T cells in 20% of unexposed individuals, more than 50% of SARS-CoV-2 convalescents, and 97% of subjects treated with the Pfizer–BioNTech COVID-19 vaccine. Although ubiquitous, these coronavirus-reactive T cells decreased with age, which may explain in part the increased susceptibility of elderly people to COVID-19. —STS

## Abstract (structured)

**INTRODUCTION:**

The clinical manifestations of COVID-19 vary from asymptomatic infection to respiratory failure. Severe disease courses are primarily associated with advanced age, immune dysfunctions, and comorbidities. Initially, severe acute respiratory syndrome coronavirus 2 (SARS-CoV-2) was thought to encounter an immunologically unprotected population. However, SARS-CoV-2 has significant homologies with endemic seasonal cold coronaviruses (HCoVs), and recent HCoV infection is associated with a less severe course of COVID-19, suggesting a protective role of cross-reactive immunity.

**RATIONALE:**

The preexistence of SARS-CoV-2–cross-reactive CD4^+^ T cells in unexposed individuals has been repeatedly demonstrated, but their contribution to host responses is an active area of investigation. Here, we investigated the functional role of preexisting SARS-CoV-2–cross-reactive and HCoV-reactive CD4^+^ T cells with high resolution.

**RESULTS:**

We demonstrate broad CD4^+^ T cell cross-reactivity in unexposed individuals, with the spike glycoprotein serving as one of the immunodominant targets. Although the N-terminal part of spike (covered by the S-I peptide pool) did not elicit cross-reactive T cell responses, the more HCoV-homologous C-terminal section (covered by the S-II peptide pool) induced T cell responses in unexposed donors. We identified a universal immunodominant coronavirus peptide located within the fusion peptide domain of spike (S816-830) recognized by CD4^+^ T cells in 20% of unexposed individuals, 50 to 60% of SARS-CoV-2 convalescents, and 97% of BNT162b2-vaccinated individuals. S816-830– and spike–cross-reactive T cells were recruited in immune responses to SARS-CoV-2 infection and BNT162b2 COVID-19 mRNA vaccination. S816-830-reactive T cells initially contributed up to 100% of the S-II–reactive CD4^+^ T cells but their proportion decreased during the course of the S-II–specific immune response. Upon primary vaccination, cross-reactive cellular and humoral immunity exhibited kinetics typical for secondary immune responses. The frequencies of preexisting cross-reactive T cells correlated positively with functional avidity of the T cell receptor, as well as with the induction and stabilization of anti–SARS-CoV-2-S1-IgG and neutralizing antibodies. Although HCoV-responsive T cells are ubiquitous, their frequencies and the frequencies of SARS-CoV-2–cross-reactive CD4^+^ T cells decreased with age, consistent with an increased vulnerability of the elderly to severe COVID-19 disease.

**CONCLUSION:**

Preexisting cross-reactive CD4^+^ T cells enhance immune responses in SARS-CoV-2 infection and BNT162b2 vaccination. Because these cells are greatly diminished in the elderly, our results suggest that their decrease may contribute to the increased susceptibility of this population to severe COVID-19. Preexisting cross-reactive immunity may be responsible for the unexpectedly rapid induction of protective immunity after primary SARS-CoV-2 immunization and the high rate of asymptomatic and mild COVID-19 disease courses.

**Figure:**
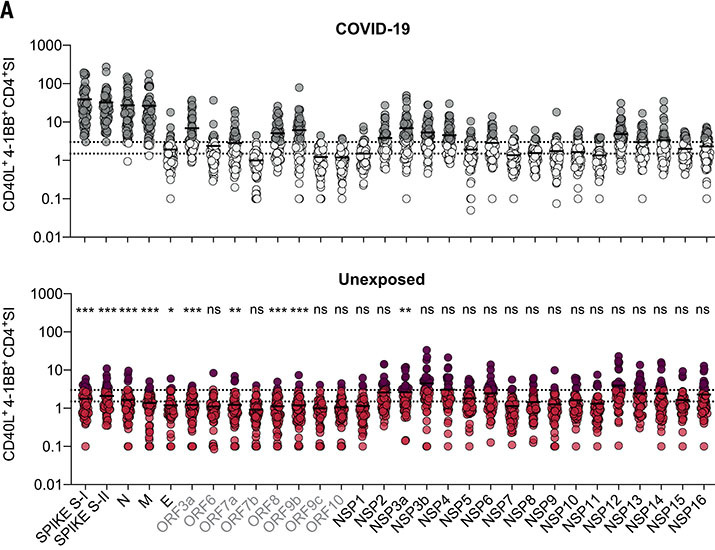

## Teaser

Preexisting spike–cross-reactive T cells correlate with higher neutralizing anti-spike protein antibodies in SARS-CoV-2 infection and vaccination

## Graphical abstract

**Figure:**
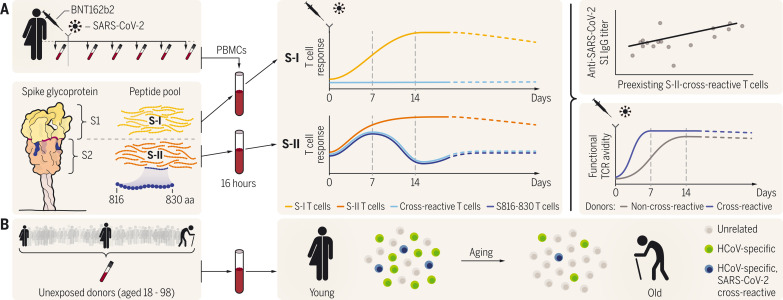
Cross-reactive CD4^+^ T cells enhance SARS-CoV-2 immune responses upon infection and vaccination. (**A**) Preexisting SARS-CoV-2 S-II– and spike (S)816-830–cross-reactive T cells show booster response characteristics upon SARS-CoV-2 infection and vaccination and their abundance correlates with higher protective anti–SARS-CoV-2 S1-IgG titers and higher functional T cell avidity. (**B**) The frequency of HCoV-reactive and SARS-CoV-2–cross-reactive T cells decreases with age.

Most individuals infected with severe acute respiratory syndrome coronavirus 2 (SARS-CoV-2) experience an asymptomatic or mild course of COVID-19. However, severe or fatal disease occurs in ~5% of those infected and is primarily associated with advanced age and comorbidities such as diabetes and chronic cardiovascular, pulmonary, and kidney diseases (*1*). Given that SARS-CoV-2 is a newly emerged human pathogen, it was assumed that SARS-CoV-2 encounters an immunologically naive population. However, SARS-CoV-2 displays considerable homologies with endemic human common cold coronaviruses collectively referred to as “HCoV” (*2*, *3*). There is now strong evidence for cellular and humoral cross-reactivity to SARS-CoV-2 (*3*–*14*), although the role of cross-reactive immunity in SARS-CoV-2 infection is unclear (*2*, *8*, *15*, *16*). Recent HCoV infection is associated with less severe COVID-19, suggesting a protective role (*17*). A better understanding of the extent and impact of cross-immunity in SARS-CoV-2 infection and vaccination is needed because cognate cross-immunity may influence the efficacy of vaccination regimens.

Here, we investigated the functional role of preexisting SARS-CoV-2– and HCoV-reactive CD4^+^ T cells. The SARS-CoV-2 spike glycoprotein (spike) was the dominant target of broad T cell cross-reactivity in unexposed individuals, which decreased with age. We identified an immunodominant coronavirus peptide located within the fusion peptide domain of spike (S816-830) recognized by CD4^+^ T cells in 20% of unexposed individuals, 50 to 60% of SARS-CoV-2 convalescents, and 97% of BNT162b2-vaccinated individuals. S816-830– and spike–cross-reactive T cells were recruited into primary SARS-CoV-2 immune responses and also into BNT162b2 COVID-19 mRNA vaccination responses. Finally, upon primary vaccination, cross-reactive immunity exhibited kinetics similar to those in secondary immune responses. Already at an early stage of the immune response, the frequencies of preexisting cross-reactive T cells correlated positively with functional avidity as well as with the induction and stabilization of anti–S1-IgG antibodies. Thus, cross-reactive CD4^+^ T cells accelerate the immune response in SARS-CoV-2 infection and vaccination. These findings add to the discussion surrounding single-dose vaccination of healthy adults and multiple-dose vaccination of the elderly.

## Frequent and broad SARS-CoV-2 cross-reactivity in unexposed healthy donors

**Fig. 1.**
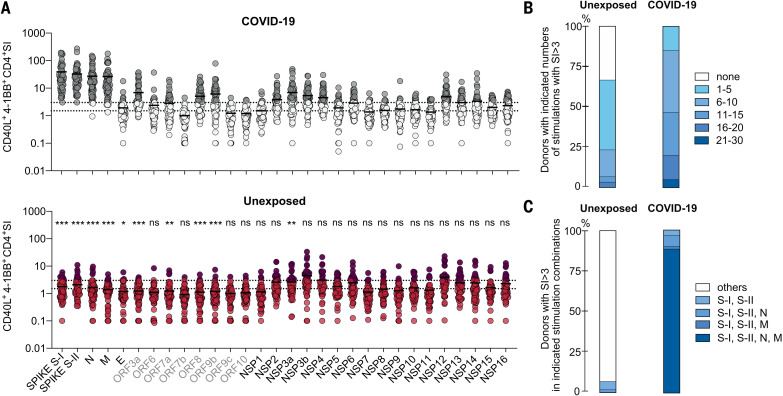
CD4^+^ T cell cross-reactivity against the SARS-CoV-2 orfeome. (**A**) Ex vivo stimulation of PBMCs from COVID-19 convalescent patients (top panel, *n* = 59) and unexposed individuals (bottom panel, *n* = 60). The percentage of CD40L^+^4-1BB^+^ CD4^+^ T cells among stimulated PBMCs was divided by the percentage of these cells among unstimulated PBMCs to determine the SI shown on the *y*-axis. The SARS-CoV-2 orfeome peptide pools used for stimulation are shown below the bottom panel. Gray labels highlight proteins exclusive for SARS-CoV-2 (i.e., those not shared with HCoVs). Gray circles (COVID-19) or red circles (unexposed) identify donors with an SI ≥ 3. Dotted lines indicate an SI of 1.5 and 3. Statistically significant differences between COVID-19 convalescents and unexposed groups (with respect to each peptide pool) are indicated above the bottom panel. *P < 0.05, **P < 0.01, and ***P < 0.001; ns, not significant at *P* > 0.05 by unpaired Student’s *t* test. (**B**) Bars show the proportions of individuals with the indicated number of SARS-CoV-2 orfeome peptide pool stimulations with an SI ≥ 3. (**C**) Proportions of individuals with an SI ≥ 3 for each stimulation in each indicated stimulation combination.

To determine the extent of cellular cross-reactivity to SARS-CoV-2 antigens, we stimulated CD4^+^ T cells of 60 unexposed healthy donors and 59 COVID-19 convalescents as controls (table S1) with peptide pools covering all open reading frames (ORFs) of SARS-CoV-2, referred to here as the “SARS-CoV-2 orfeome” (Fig. 1A). The SARS-CoV-2 orfeome consists of 11 ORFs, five of which [N, spike, E, M, and ORF1a/b (encoding for the nonstructural proteins (NSPs) 1 to 16)] are also found in HCoVs 229E, OC43, NL63, and HKU1. Amino acid sequence alignment revealed discrete areas of high homology in almost all SARS-CoV-2 proteins to the corresponding proteins in HCoVs. Parts of the ORF1a/b including NSP8, NSP10, and NSP12-16 displayed the highest degree of homology and thus potential cross-reactive epitopes to all HCoVs (fig. S1A). Nevertheless, COVID-19 convalescents did not show significantly increased CD4^+^ T cell reactivity against the NSPs compared with unexposed individuals (Fig. 1A and fig. S1B). Reactivity against the combination of spike N-terminal S-I (amino acid residues 1 to 643), C-terminal S-II (amino acid residues 633 to 1273), N, and M peptide pools clearly distinguished COVID-19 convalescents from unexposed individuals irrespective of the disease course (Fig. 1, A to C). In unexposed individuals, we detected variable but low CD4^+^ T cell reactivity to virtually all SARS-CoV-2 antigens, including those exclusive to SARS-CoV-2 (not shared with HCoVs). However, the degree of amino acid sequence homology between HCoV and SARS-CoV-2 proteins did not correlate with cross-reactivity (fig. S1C). Thus, apart from cognate cross-reactivity (resulting, for example, from previous exposure to similar proteins found in HCoVs), we also identified noncognate cross-reactivity (i.e., cross-reactivity that cannot be explained by the previous exposure to similar proteins in HCoVs). Of all 30 orfeome peptide pools, the spike S-I and S-II pools alone elicited T cell reactivity in all COVID-19 convalescents and in a subset of unexposed individuals. Because antibodies to spike induced by SARS-CoV-2 infection can neutralize the virus (*18*) and most of the recently approved SARS-CoV-2 vaccines are highly effective and include spike as the main vaccine antigen, we examined cellular immunity to spike more closely.

## SARS-CoV-2 spike S-II–cross-reactive T cells decrease with age

**Fig. 2.**
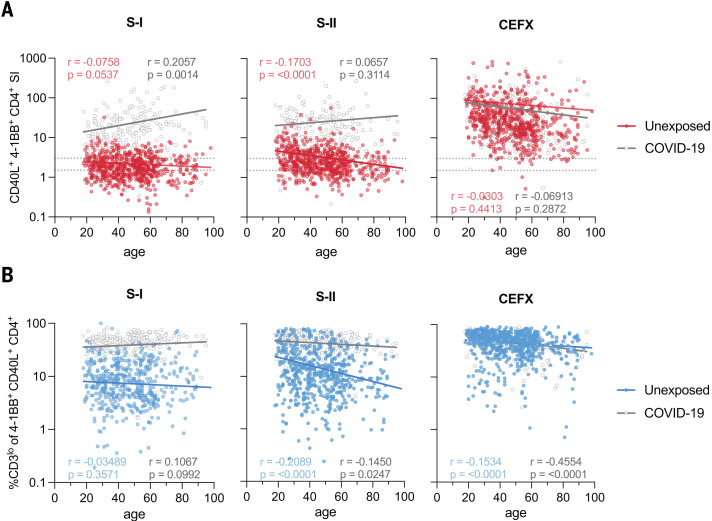
The magnitude of SARS-CoV-2 cross-reactivity decreases with age. (**A**) Scatter plots showing the SI (CD40L^+^41BB^+^ CD4^+^ T cells) among PBMCs stimulated with SARS-CoV-2 S-I, SARS-CoV-2 S-II, or CEFX (known T cell–stimulating peptides from CMV, EBV, influenza virus, and other common pathogens) plotted against age in *n* = 568 unexposed donors and *n* = 174 COVID-19 convalescents. Dotted lines indicate an SI of 1.5 and 3. (**B**) Frequencies of CD3^lo^ cells among S-I–, S-II–, or CEFX-reactive CD40L^+^4-1BB^+^ CD4^+^ T cells over age. CD3^lo^ frequencies are shown for T cell responses with an SI ≥ 1.5. Regression lines denote linear regression on age in each group; the corresponding Pearson correlation coefficients are shown.

A distinct feature of SARS-CoV-2 infection is the strong correlation of higher age with disease severity. Immunosenescence is associated with a lack of newly generated T cells and, instead, the expansion of a small number of clones resulting from persistent infections, which limits the breadth and quality of T cell responsiveness (*19*, *20*). To assess the impact of age on SARS-CoV-2–cross-reactive T cell immunity, we examined SARS-CoV-2 spike–specific CD4^+^ T cell responses in 568 unexposed individuals and 174 COVID-19 convalescents (Fig. 2A and table S1). T cells reacting to a peptide pool representing a mixture of selected T cell epitopes from common pathogens (the CEFX pool) remained relatively stable with age in both cohorts (Fig. 2A). COVID-19 convalescents displayed a significant age-associated increase in spike S-I–reactive T cells that correlated with higher disease severity in the elderly (table S1). However, consistent with our previous findings (*3*) in unexposed individuals, T cell cross-reactivity to spike S-I was rare, close to the limit of detection, and remained stable, albeit at low levels with increasing age. By contrast, reactivity to S-II was more frequent and generally higher in unexposed individuals but significantly decreased with increasing age (Fig. 2A). When total CD4^+^ T cells were analyzed for activation-induced interferon-γ (IFN-γ) or tumor necrosis factor-α (TNF-α) expression, we did not detect any age-related changes (fig. S2A). However, among bona fide T cell receptor (TCR)–activated antigen-specific CD40L^+^4-1BB^+^ CD4^+^ T cells, IFN-γ^+^TNF-α^+^ cells decreased with age (fig. S2B). In contrast to CD40L^+^4-1BB^+^ CD4^+^ T cells, total CD40L^+^ CD4^+^ T cells, which can also be induced in part in a TCR-independent manner (*21*), did not decrease with age, consistent with the large compartment of memory T cells in older individuals (fig. S2C). Thus, elderly individuals exhibit decreased cognate cross-reactive immunity to the SARS-CoV-2 spike S-II portion, which exhibits higher homology to HCoV than the S-I portion.

## Low CD3 surface expression identifies SARS-CoV-2–reactive T cells with high functional avidity ex vivo

To assess the quality of the spike–cross-reactive T cell response in terms of functional T cell avidity, we examined the level of CD3 surface expression in CD40L^+^4-1BB^+^ CD4^+^ T cells after short-term in vitro stimulation (Fig. 2B). Strong TCR activation, which is characteristic of T cells with high TCR avidity, blocks recycling of the TCR-CD3 complex and can be detected by reduced CD3 surface expression (*22*), a phenomenon known as high functional avidity. Thus, cognate cross-reactivity with higher probability of high functional avidity is distinguishable from noncognate cross-reactivity with higher probability of low functional avidity by analyzing the frequency of CD3^lo^ T cells among TCR-activated CD4^+^ T cells (fig. S3, A and B). After stimulation with spike S-I and S-II peptide pools, COVID-19 convalescents showed high frequencies of S-I– and S-II–activated CD4^+^ T cells that largely lacked CD3 expression characteristic of cognate T cell activation (Fig. 2B). In unexposed individuals, however, the frequency of CD3^lo^ cells among S-I– and S-II–activated CD4^+^ T cells was markedly lower. Nevertheless, especially in the younger subjects, spike S-II stimulation induced higher frequencies of CD3^lo^ cells than S-I stimulation, indicating that spike S-I–cross-reactive CD4^+^ T cells have high functional avidity (Fig. 2B). This is consistent with the high degree of homology between the C-terminal S-II portions of SARS-CoV-2 spike and HCoV spike proteins.

## Frequency of HCoV spike–reactive high-functional-avidity CD4^+^ T cells decreases with age

**Fig. 3.**
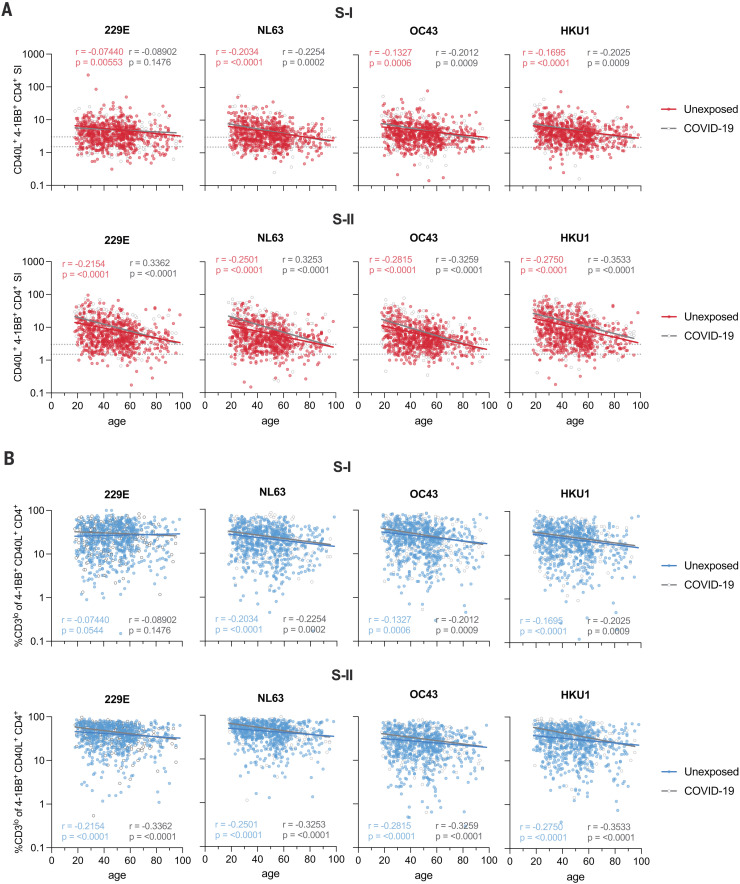
Frequencies of high-functional-avidity T cells specific for spike S-II from HCoVs decrease with age. (**A**) Scatter plots show the SI of CD40L^+^4-1BB^+^ CD4^+^ T cells in unexposed individuals (*n* = 568) and COVID-19 convalescents (*n* = 174) after PBMC stimulation with HCoV (229E, NL63, OC43, and HKU1) spike S-I or S-II peptide pools plotted against age. Dotted lines indicate an SI of 1.5 and 3. (**B**) Frequencies of CD3^lo^ cells in CD40L^+^4-1BB^+^ CD4^+^ T cells from unexposed and COVID-19 convalescents plotted against age. CD3^lo^ frequencies are shown for T cell responses with an SI ≥ 1.5. Regression lines denote linear regression on age in each group; the corresponding Pearson correlation coefficients are shown.

We hypothesized that previous HCoV exposures induce cognate cross-reactive CD4^+^ T cells. Therefore, we next characterized CD4^+^ T cell immunity to HCoV spike in unexposed individuals and COVID-19 convalescents. HCoV-S-I– and HCoV-S-II–reactive CD4^+^ T cells were more readily detectable than SARS-CoV-2 spike–specific T cells and found in 80% (S-I) and 98% (S-II) of SARS-CoV-2–unexposed individuals, respectively (Fig. 3A). Their frequency decreased with age, and SARS-CoV-2 infection did not result in an increase in HCoV-S-I– or HCoV-S-II–reactive T cells. We also examined the functional avidities of HCoV-reactive CD4^+^ T cells (Fig. 3B). High frequencies of CD3^lo^ T cells were found among both HCoV-S-I– and HCoV-S-II–reactive CD4^+^ T cells, although they significantly decreased with advancing age. Thus, a high degree of HCoV exposure in the population appears to lead to widespread cross-reactivity to the SARS-CoV-2 spike protein. HCoV-reactive CD4^+^ T cells frequently contain a subset of cells with high functional avidity but significantly decrease with age.

## The immunodominant peptide S816-830 is recognized by SARS-CoV-2 spike S-II–cross-reactive CD4^+^ T cells

**Fig. 4.**
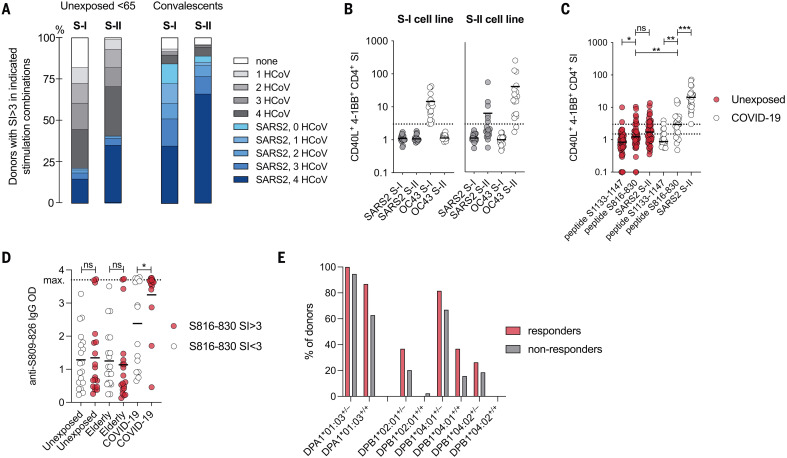
Peptide S816-830 constitutes an immunodominant epitope of SARS-CoV-2 T cell cross-reactivity. (**A**) Bars show the proportions of unexposed individuals <65 years of age (*n* = 491) and COVID-19 convalescents (*n* = 174, 18–79 years) with S-I– or S-II–specific T cell responses to HCoV and/or SARS-CoV-2 with an SI ≥ 3. (**B**) Plots showing the SI (CD40L^+^4-1BB^+^ CD4^+^ T cells) of short-term T cell lines derived from OC43 S-I– and S-II–reactive primary T cells after restimulation with autologous antigen-presenting cells in the presence of OC43 or SARS-CoV-2 spike glycoprotein pools S-I and S-II. The dotted line indicates an SI of 3. (**C**) The SIs of CD40L^+^4-1BB^+^ CD4^+^ T cells from unexposed (*n* = 48) or COVID-19 convalescents (*n* = 22) after stimulation with the single peptide 204_3 (S816-830), the control single peptide 284 (S1133-1147), or the S-II peptide pool. (**D**) Levels (optical density, OD) of anti–S809-826 peptide IgG (ELISA) in unexposed young (<65 years) and elderly (>65 years) individuals as well as COVID-19 convalescents. ELISA plates were coated with an 18-aa peptide overlapping by 11 aa with S816-30. Serum was diluted 1:100. (**E**) Bars show the frequencies of common class-II HLA alleles in definite S816-830 responders (SI ≥ 3) and definite nonresponders (SI < 1.5) (*n* = 308). +/+, homozygous; +/–, heterozygous. *P < 0.05, **P < 0.01, ***P < 0.001; ns at *P* > 0.05 by Student’s *t* test.

All SARS-CoV-2–cross-reactive unexposed donors showed a response against at least two (S-I) or three (S-II) HCoVs, suggesting that repeated infection with different HCoVs establishes a detectable prominent SARS-CoV-2–cross-reactive T cell pool already early in life and/or that specific T cells are directed against highly homologous sequences shared across multiple HCoVs and SARS-CoV-2 (Fig. 4A). We next investigated whether HCoV spike glycoprotein-specific T cells directly cross-react to SARS-CoV-2 spike glycoprotein. Short-term CD40L^+^4-1BB^+^ OC43 S-I– or S-II–reactive CD4^+^ T cell lines were restimulated with OC43- or SARS-CoV-2 spike pool S-I and S-II, respectively. Six out of 18 OC43 S-II–specific T cell lines displayed cross-reactivity against SARS-CoV-2 S-II, whereas OC43 S-I–specific T cell lines lacked cross-reactivity against SARS-CoV-2 S-I (Fig. 4B). We further identified and validated two overlapping T cell–stimulating peptides, peptides 204 (SKRSFIEDLLFNKVT, amino acids 813 to 827) and 205 (FIEDLLFNKVTLADA, amino acids 817 to 831), derived from the S-II portion of spike, in all five donors analyzed (fig. S4, A to D). Only one donor responded to other identified peptides (peptides 188, 189, and 251) (fig. S4B). Sequence alignment revealed that S-II peptides 204 and 205 together covered the fusion peptide domain of spike, which is characterized by strong homology with HCoV (fig. S4C). By analyzing additional 15-aa peptides along the sequence covered by the peptides 204 and 205, we identified the sequence SFIEDLLFNKVTLAD (amino acids 816 to 830) as an immunodominant coronavirus peptide, hereafter referred to as S816-830 (peptide 204_3; fig. S4D). We next examined direct ex vivo T cell reactivity against S816-830 compared with a control peptide 284 (amino acids 1133 to 1147, hereafter referred to as S1133-1147) and the SARS-CoV-2 spike S-II peptide pool in 48 unexposed individuals and 22 COVID-19 convalescents. S816-830–reactive CD4^+^ T cells were detected in 50% of convalescents and 20% of unexposed individuals, with significantly higher frequencies in the former (Fig. 4C). Antibodies to the SARS-CoV-2 spike amino acid residues S809 to S826 were previously reported in COVID-19 patients but also in unexposed individuals (*23*, *24*). When we examined the sera of responders and nonresponders to the S816-830 T cell assay, we detected S809-826–binding antibodies in all individuals. However, significantly higher concentrations of these antibodies were found in COVID-19 convalescents with substantially more S816-830–reactive T cells (Fig. 4D). Compared with definite nonresponders [stimulation index (SI) < 1.5], definite S816-830 peptide responders (SI ≥ 3) were more frequently positive for HLA-DPB1*02:01, HLA-DPB1*04:02, and especially homozygous expression of HLA-DPB1*04:01 (Fig. 4E). Because HLA-DPA1*01:03 was found in 100% of the responders and 94.8% of the nonresponders, we investigated whether combinations of HLA-DPA1*01:03 and HLA-DPB1*02:01/DPB1*04:01/DPB1*04:02 were likely to present peptide S816-830 or fragments thereof. HLA-peptide–binding predictions identified excellent potential binders (fig. S4E), which was also true for the homologous S816-830 peptide in other HCoVs (fig. S4F).

## Preexisting SARS-CoV-2 spike S-II–cross-reactive T cells are recruited into primary SARS-CoV-2 immune responses

**Fig. 5.**
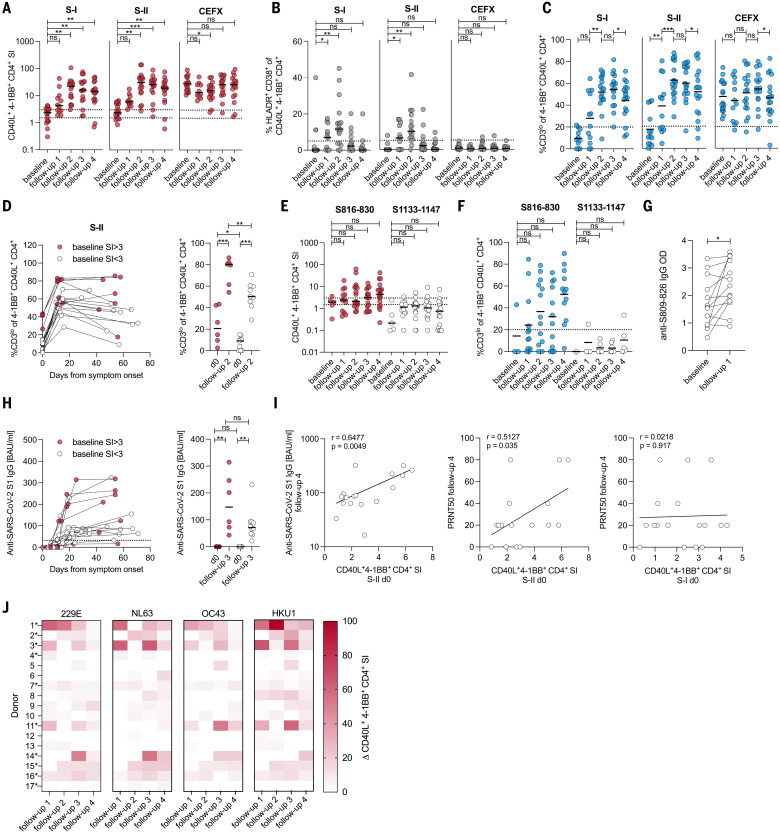
HCoV-specific SARS-CoV-2–cross-reactive T cells are recruited into the primary SARS-CoV-2 infection response. (**A** to **C**) SI of CD40L^+^4-1BB^+^ CD4^+^ T cells (A), frequencies of HLADR^+^CD38^+^ cells (B), and frequencies of CD3^lo^ cells (C) among CD40L^+^4-1BB^+^ CD4^+^ T cells after stimulation with SARS-CoV-2 S-I, S-II, and CEFX peptide pools of donors before infection (baseline) and at four different follow-up time points (table S2) after symptom onset. CD3^lo^ frequencies are shown only for T cell responses with an SI ≥ 1.5. (**D**) Changes to CD3^lo^ frequencies among CD40L^+^4-1BB^+^ CD4^+^ T cells between baseline, follow-up 2 (10 to 16 days after symptom onset), and follow-up 4 (29 to 71 days after symptom onset) (left plot), and statistics (right plot) for baseline and follow-up measurement time point 2 in cross-reactive donors (baseline SI ≥ 3, red circles) and non–cross-reactive donors (baseline SI < 3, white circles). (**E** and **F**) SI (E) and frequency (F) of CD3^lo^ of CD40L^+^4-1BB^+^ CD4^+^ T cells after stimulation with peptide S816-830 or control peptide S1133-1147. CD3^lo^ frequencies are shown for T cell responses with an SI ≥ 1.5. (**G**) Levels (OD) of anti–S809-826 peptide IgG (ELISA) at baseline and follow-up time point 1 (3 to 9 days after symptom onset). ELISA plates were coated with an 18-aa peptide overlapping by 11 aa with S816-830. (**H**) Anti–S1-IgG binding antibody units (BAUs) in cross-reactive (baseline SI ≥ 3, red circles) and non–cross-reactive donors (baseline SI < 3, white circles) were plotted against time (left) and compared between baseline and follow-up 3 (right). (**I**) Scatter plots showing the relationship between anti–SARS-CoV-2 S1 IgG antibody levels (OD) at follow-up 4 and the SI of CD40L^+^4-1BB^+^ CD4^+^ T cells upon S-II stimulation at baseline (left), the relationship between neutralizing antibody titers (PRNT50) at follow-up 4, and the SI of CD40L^+^4-1BB^+^ CD4^+^ T cells upon S-II stimulation (left) or S-I stimulation (right) at baseline. (**J**) Heatmap showing the change in SI of CD40L^+^4-1BB^+^ CD4^+^ T cells after stimulation with S-II pools of the indicated HCoVs. Δ represents the change of the parameter at the given time point relative to baseline (i.e., white depicts no increase). Asterisks indicate S816-830 peptide responders. For (A), (B), (E), and (F), *P < 0.05, **P < 0.01, ***P < 0.001, and ns at *P* > 0.05 by repeated-measures one-way-ANOVA with Dunnett’s correction. For (C) and (G), *P < 0.05, **P < 0.01, ***P < 0.001, and ns at *P* > 0.05 by paired Student’s *t* test. For (D) and (H), *P < 0.05, **P < 0.01, ***P < 0.001, and ns at *P* > 0.05 by Student’s *t* test. For (E), ns at *P* > 0.05 by paired Student’s *t* test. For (I) ns at *P* > 0.05 by Pearson correlation.

A still open question is whether and to what extent SARS-CoV-2–cross-reactive T cells influence the disease course of primary SARS-CoV-2 infection. By monitoring the healthy, previously unexposed study participants for primary SARS-CoV-2 infection, we identified 17 cases of acute primary SARS-CoV-2 infection (Fig. 5 and table S2). All 17 patients showed detectable virus titers (fig. S5A) and mild COVID-19 disease course (no hospitalization required) (table S2). Robust CD4^+^ T cell responses specific to SARS-CoV-2 spike S-I and S-II were detected, and the proportions of HLADR^+^CD38^+^ cells among CD40L^+^4-1BB^+^ CD4^+^ T cells significantly increased at follow-up time points 1 and 2 (3 to 16 days), indicating their in vivo activation (Fig. 5, A and B). CD3^lo^ cells substantially increased during acute primary SARS-CoV-2 infection and remained at high levels after the infection resolved (Fig. 5C). Individuals who already had spike S-II–cross-reactive CD4^+^ T cells with a SI ≥ 3 at baseline showed significantly higher functional avidity throughout the initiation of the T cell response (Fig. 5D). S816-830–reactive T cells increased in both frequency and in functional avidity in 10 of 17 donors after infection (Fig. 5, E and F). Immunoglobulin G (IgG) antibodies against the S809-826 peptide were boosted as early as 3 to 9 days (follow-up time point 1) after the presumed infection (Fig. 5G). Anti–SARS-CoV-2-S1-IgG serum antibodies were detectable at follow-up time point 2 and peaked after day 20 in most individuals, although their kinetics and quantity varied widely (Fig. 5H). Anti–SARS-CoV-2-S1 binding antibody (IgG) units at late time points positively correlated with S-II– but not S-I–cross-reactive T cell levels at day 0, suggesting that preexisting cross-reactive CD4^+^ T cells enhance SARS-CoV-2–specific humoral immunity (Fig. 5I, left). Moreover, the neutralizing antibody titers also positively correlated with spike S-II– but not spike S-I–cross-reactive CD4^+^ T cells at baseline, suggesting a protective role of cross-reactive CD4^+^ T cells (Fig. 5I, middle and right). Finally, the frequency of HCoV-reactive CD4^+^ T cells also increased in almost all individuals shortly after primary SARS-CoV-2 infection (Fig. 5J). There was a concomitant increase in the frequency of CD3^lo^ cells (fig. S5B) and HLADR^+^CD38^+^ cells (fig. S5C) among HCoV-reactive CD4^+^ T cells, demonstrating that preexisting HCoV-reactive cellular immunity was activated and transiently expanded during primary SARS-CoV-2 infection. Clearly, preexisting SARS-CoV-2 S-II–cross-reactive CD4^+^ T cells were recruited into primary SARS-CoV-2 immune responses in healthy, previously unexposed individuals. Thus, the quantity and functional avidity of preexisting cross-reactive cellular immunity corresponds to the quality and magnitude of specific cellular and humoral anti–SARS-CoV-2 responses. It may therefore contribute to a milder course of COVID-19 by limiting viral propagation.

## BNT162b2 vaccination reactivates preexisting SARS-CoV-2 spike S-II–cross-reactive T cells

**Fig. 6.**
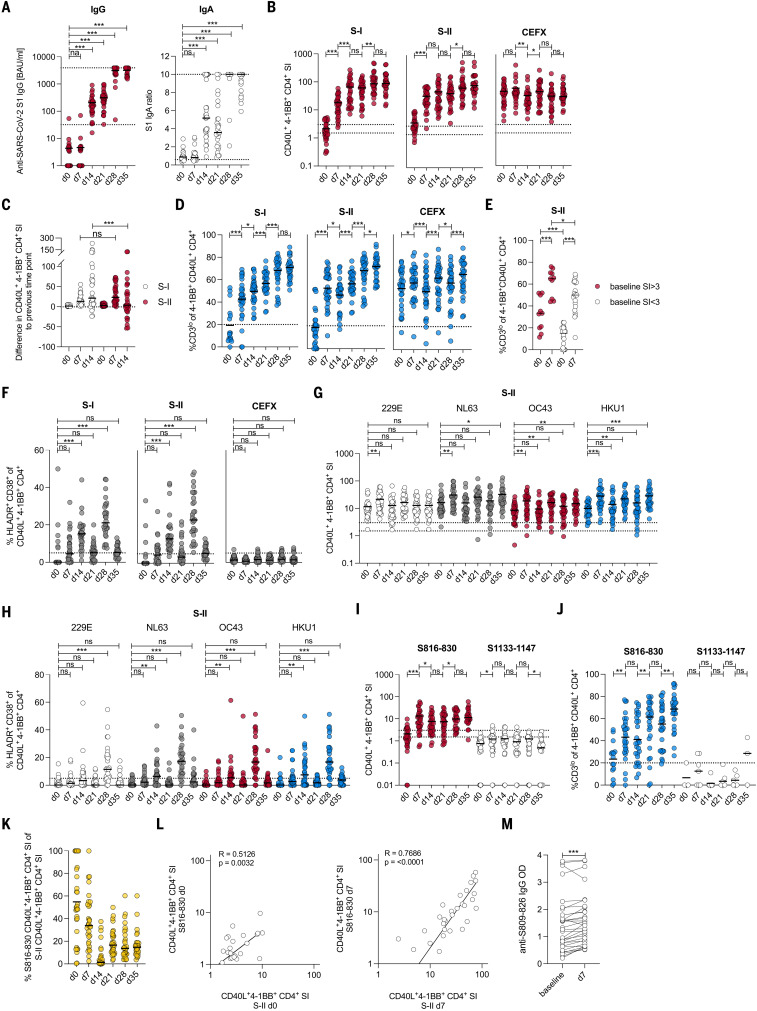
HCoV-specific SARS-CoV-2–cross-reactive T cells are recruited into the BNT162b2 vaccine response. (**A**) Serum anti-SARS-CoV-2 S1 IgG BAUs and IgA titer ratio were determined at baseline, day 7, and day 14 after primary vaccination with BNT162b, immediately before secondary vaccination (day 21) as well as 1 (day 28) and 2 weeks (day 35) after secondary vaccination. All values <1 were set to 1. The lower and upper cut-off levels for IgG were set at 32 and 3900, respectively; the corresponding IgA cut-offs were set at 0.6 and 10, respectively, indicated by dotted lines. (**B**) Plots showing the SI of CD40L^+^4-1BB^+^ CD4^+^ T cells after stimulation with S-I, S-II, and CEFX at baseline and at the indicated time points. (**C**) Difference in SI after stimulation with S-I and S-II at each time point relative to the previous time point. (**D**) Plots showing the frequencies of CD3^lo^ of CD40L^+^4-1BB^+^ CD4^+^ T cells after stimulation with S-I, S-II, and CEFX for responses with an SI ≥ 1.5. (**E**) Frequencies of CD3^lo^ of CD40L^+^4-1BB^+^ CD4^+^ T cells at days 0 and 7 in cross-reactive donors (baseline SI ≥ 3, red circles) and non–cross-reactive donors (baseline SI < 3, white circles). (**F**) Frequencies of HLADR^+^CD38^+^ cells among CD40L^+^4-1BB^+^ CD4^+^ T cells after stimulation with S-I, S-II, and CEFX at the indicated time points. (**G** and **H**) SI of CD40L^+^4-1BB^+^ CD4^+^ T cells (G) and frequencies of HLADR^+^CD38^+^ among these cells (H) after stimulation with HCoV S-II peptide pools at baseline and the indicated time points. (**I** and **J**) SI of CD40L^+^4-1BB^+^ CD4^+^ T cells (I) and frequencies of CD3^lo^ events (SI ≥ 1.5) (J) among these cells after stimulation with peptide S816-830 and control peptide S1133-1147 at baseline and the indicated time points. (**K**) Proportion of S816-830–reactive T cells over SARS-CoV-2 S-II–reactive T cells. (**L**) Relationship between responses to S816-830 and SARS-CoV-2 S-II peptide pool stimulation at day 0 (left) and day 7 (right). (**M**) OD of anti–S809-826-peptide IgG ELISA from sera before and 7 days after primary vaccination. For (A) and (F) to (J), *P < 0.05, **P < 0.01, ***P < 0.001, and ns at *P* > 0.05 by repeated-measures one-way-ANOVA with Dunnett’s correction. For (B) to (D) and (M), *P < 0.05, **P < 0.01, ***P < 0.001, and ns at *P* > 0.05 by paired Student’s *t* test. For (E), *P < 0.05, **P < 0.01, ***P < 0.001, and ns at *P* > 0.05 by Student’s *t* test. For (L), ns at *P* > 0.05 by Pearson correlation.

Finally, we investigated how preexisting SARS-CoV-2 S-II–cross-reactive T cells in healthy, unexposed individuals influence the course of BNT162b2 COVID-19 spike mRNA vaccine responses. We monitored baseline and follow-up humoral and T cell responses against SARS-CoV-2 and HCoV spike glycoproteins in 31 healthy adults who underwent primary (day 0) and booster (day 21) vaccination with BNT162b2. At day 21, 30 of 31 donors had detectable anti–SARS-CoV-2 S1 IgG, and all donors had detectable anti–SARS-CoV-2 S1 IgA levels (Fig. 6A). Booster vaccination further increased these antibody levels. Primary vaccination also induced robust S-I– and S-II–reactive CD4^+^ T cell responses in all individuals that were only slightly enhanced by booster vaccination (Fig. 6B). The kinetics of S-I– and S-II–reactive T cells differed in that S-II–reactive T cells showed a sharp increase from baseline to day 7 but not thereafter, whereas S-I–reactive T cells showed an additional significant increase from day 7 to day 14 (Fig. 6, B and C). This was indicative of the secondary response kinetics of S-II–reactive cells and the primary response kinetics of S-I–reactive cells. High-functional-avidity, CD3^lo^ CD40L^+^4-1BB^+^ CD4^+^ T cells increased more rapidly in cross-reactive donors (Fig. 6, D and E). Moreover, at day 14, S-I– and S-II–reactive CD4^+^ T cells included high frequencies of HLADR^+^CD38^+^ cells in all but three donors, indicating their recent in vivo activation (Fig. 6F). Like SARS-CoV-2–specific T cells, HCoV S-II–reactive T cells were significantly increased 7 days after primary vaccination (Fig. 6G). This was associated with an increased frequency of HCoV S-II–reactive HLADR^+^CD38^+^ T cells (Fig. 6H). Thus, cognate cross-reactive T cells were activated early in response to SARS-CoV-2 spike–specific vaccination but did not expand thereafter. All but two of 31 donors (94%) responded with T cells that had high functional avidity to S816-830 at days 7 and 14 (Fig. 6, I and J). S816-830–reactive T cells initially contributed up to 100% of the CD40L^+^4-1BB^+^ cells in S-II stimulations, but their proportion decreased as other specificities increased during the course of the SARS-CoV-2 S-II–specific immune response (Fig. 6K). Thus, HCoV imprinting does not appear to hamper an immune response tailored to SARS-CoV-2. We observed a correlation between the S816-830–reactive and the S-II–reactive T cell response at day 0 that was even more pronounced at day 7, emphasizing the importance of the S816-830 peptide in the early stages of the anti–SARS-CoV-2 cellular immune response (Fig. 6L). A humoral response to S809-826 (overlapping with S816-830) was detectable as early as 7 days after primary vaccination (Fig. 6M) and this was distinct from the slower anti–SARS-CoV-2-S1-IgG response. This supports the concept that preexisting cross-reactive immunity mediates secondary response kinetics (*26*).

## Discussion

The functional relevance of preexisting cognate cross-immunity to SARS-CoV-2 is a subject of intense debate. Noncognate cross-reactivity has been reported but appears to play a minor role compared with HCoV-mediated cognate cross-reactivity (*15*). A recent HCoV infection is associated with a less severe course of COVID-19 (*17*). Unexpectedly, >90% of the population is HCoV-seropositive. Thus, a large proportion of the population might benefit from cross-reactive humoral immunity (*27*, *28*). However, prepandemic serum from PCR-validated HCoV-positive individuals contains neutralizing antibodies against all HCoVs but not SARS-CoV-2 (*27*). In a subsequent study, only low spike-specific cross-reactive antibody activity was detected in just five of 34 donors with recent HCoV infection and in just one of 31 donors without recent HCoV infection, indicating that humoral cross-immunity is weak and decays rapidly (12). Finally, although infection with SARS-CoV-2 increases the prevalence of antibodies against seasonal HCoVs, these antibodies do not provide protection, which highlights the role of cross-reactive cellular immunity (*9*, *27*, *28*).

Recently, T cells cross-reactive to several SARS-CoV-2 antigens were identified in unexposed individuals using predicted peptides individually (*4*, *5*) or as megapools (*8*, *29*). Our work reveals significant cross-reactivity of ORF1a/b–encoded proteins but also shows that most of the anti-SARS-CoV-2 reactivity is directed against the spike, N, and M proteins. We further demonstrate that the magnitude and quality of SARS-CoV-2 cross-reactivity and HCoV reactivity declines with age. The failure of an aging immune system to maintain HCoV-induced SARS-CoV-2–cross-reactive T cells along with a smaller pool of naïve T cells that can be recruited into SARS-CoV-2–specific responses (*20*) may contribute to the increased susceptibility of elderly to severe COVID-19. Our results show that HCoV-specific, SARS-CoV-2–cross-reactive T cells contribute to SARS-CoV-2 immune responses upon infection and vaccination. Additionally, such cognate cross-reactivity correlates with a rapid cellular and enhanced humoral response, both of which may favor mild disease courses. The sequential administration of different haptens sharing the same carrier to mice induced preexisting T cell help for the second hapten, leading to more efficient B cell recruitment in secondary immunization (*30*). Accordingly, B cells recognizing SARS-CoV-2 may benefit from HCoV-reactive T cells cross-reacting with SARS-CoV-2 peptides. Further studies in mice showed that increasing the numbers of antigen-specific T cells at the onset of the immune response also increased B cell activation and proliferation. Moreover, the presence of cognate T cell help during viral infection promotes germinal center formation, which is required for fast and high-affinity antibody generation (*30*–*32*). Because the early induction of SARS-CoV-2 T cell reactivity has been associated with rapid viral clearance and mild disease (*33*), cross-reactive T cells that enhance the immune response to SARS-CoV-2 may well serve as a correlate of immune protection against severe COVID-19 disease courses (*34*, *35*).

Upon BNT162b2 vaccination, we observed immune responses that exceeded the response to actual SARS-CoV-2 infection in terms of spike-specific T cell and antibody levels. Responses to S-II, unlike responses to the non–cross-reactive S-I, however, displayed kinetics reminiscent of a secondary immune response (*25*, *26*). These observations may provide an explanation for the results of large studies showing the high efficacy of SARS-CoV-2 vaccines. Protection levels against SARS-CoV-2 infection have been reported to be >75% as early as 15 to 28 days after primary vaccination with BNT162b2 (*36*). In addition, just one dose of the BNT162b2 or the Astra Zeneca ChAdOx1 vaccine reduced the risk of hospitalization by 85 and 94%, respectively, at days 28 to 34 after primary vaccination, an unusually high efficacy for a primary vaccination (*36*). Similarly, a single-shot vaccination based on AdV26 adenovirus–encoded modified spike protein from Johnson & Johnson has been reported to have a vaccine efficacy of 66% and was recently approved by the U.S. Food and Drug Administration and the European Medicines Agency (*37*, *38*). Our results may provide an immunological explanation for the reported high efficacies. Conversely, in the elderly, who have waning HCoV T cell reactivity and thus reduced SARS-CoV-2 T cell cross-reactivity, additional booster vaccinations may be critical (*39*, *40*).

The immunodominant cross-reactive peptide S816-830 identified here is located within the highly conserved spike fusion peptide domain downstream of the S2′ cleavage site (*41*). We demonstrate that S816-830–reactive T cells are efficiently recruited into the SARS-CoV-2 response in most infected and nearly all vaccinated individuals. Previous reports have also shown that specific antibodies against this region are generated after SARS-CoV-2 infection and vaccination with BNT162b2 (*23*, *24*). In addition, it has been proposed that antibodies specific to the S2 portion of spike have neutralizing activity and may be involved in the early induction of protection before SARS-CoV-2 S1–specific antibodies emerge (*28*, *41*–*43*). In summary, the S816-830 peptide may serve as a conserved universal coronavirus target in the S2 portion of spike for both B cells and T cells. Enhancing the immune response to S816-830 may induce efficient protection and should be a focus of future studies.

## Materials and Methods

### Study participants

This study was approved by the institutional review board of the Charité (EA/152/20). Written informed consent was obtained from all included participants (*44*), and the study was conducted in agreement with the Declaration of Helsinki. Participants who had tested positive for SARS-CoV-2 RNA [reverse transcriptase quantitative polymerase chain reaction (RT-qPCR) from nasopharyngeal swabs] were classified as convalescent donors. All donors were assessed for age, gender, body-mass index, comorbidities, and medications (table S1). Convalescent donors were subclassified according to their symptoms into World Health Organization severity grades, and information about hospitalization or admission to an intensive care unit is provided in table S1. Day of infection was set as day –3 before reported symptom onset. Measurement day after symptom onset is indicated in the graphs or table S1. Study participants who reported symptoms typical for a SARS-CoV-2 infection were RT-qPCR tested for virus RNA, and positive donors were enrolled for follow-up measurements. Details of the follow-up cohort (age, gender, comorbidities, symptoms, measurement time points after symptom onset) are provided in table S2.

### Coronavirus RT-qPCR

RNA was extracted using the MagNA Pure 96 system and the MagNA Pure Viral NA Small Volume Kit (Roche, Germany). RNA extraction was performed from a 200-μl swab dilution (swab suspended in 4.3 ml of Cobas PCR Media, Roche), eluted in 100 μl of elution buffer. Coronavirus detection using 5 μl of the RNA eluate was based on two genomic targets (E- and N gene, TIB Molbiol, Berlin, Germany). An in vitro–transcribed RNA of equine arteritis virus was used as an internal RT and PCR control. SARS-CoV-2 was quantified using the E-gene target and by applying calibration curves and using serial diluted photometrically quantified in vitro–transcribed RNA as described previously (*45*). All RT-qPCRs were performed using a LightCycler 480 II (Roche).

### Blood and serum sampling and PBMC isolation

Whole blood was collected in lithium heparin tubes for peripheral blood mononuclear cell (PBMC) isolation and SSTII advance (all Vacutainer, BD Biosciences) tubes for serology. SSTII advance tubes were centrifuged for 10 min at 1000*g* before removing serum. Serum aliquots were frozen at –20°C until further use. PBMCs were isolated by gradient density centrifugation according to the manufacturer’s instructions (Leucosep tubes, Greiner; Biocoll, Bio&SELL).

### Ex vivo T cell stimulation

Freshly isolated PBMCs were cultivated at a concentration of 5 × 10^6^/ml in AB medium containing RPMI 1640 medium (Invitrogen) supplemented with 10% heat inactivated AB serum (Pan Biotech), 100 U/ml of penicillin (Biochrom), and 0.1 mg/ml of streptomycin (Biochrom). Stimulations were conducted with PepMix overlapping peptide pools (15-aa length with 11-aa overlaps, JPT Peptide Technologies) covering the proteins of interest, including the entire SARS-CoV-2 orfeome: the spike glycoprotein (S), NCAP-1 (N), VEMP-1 (E), VME-1 (M), AP3A (ORF3a), NS6, NS7A, NS7B, NS8, ORF9B, ORF10, Y14 (ORF9c), the ORF1a/b proteins (NSP01, NSP02, NSP03a, NSP03b, NSP04, NSP05, NSP06, NSP07, NSP08, NSP09, NSP10, NSP11, NSP12, NSP13, NSP14, NSP15, and NSP16), as well as the spike glycoproteins of HCoV-229E, HCoV-OC43, HCoV-NL63, and HCoV-HKU1 (all JPT Peptide Technologies). Single-peptide stimulations were conducted with the following peptides: 204 (N′-SKRSFIEDLLFNKVT-C′), 204_1 (N′-KRSFIEDLLFNKVTL-C′), 204_2 (N′-RSFIEDLLFNKVTLA-C′), 204_3 (N′-SFIEDLLFNKVTLAD-C′), 205 (N′-FIEDLLFNKVTLADA-C′), and the control peptide 284 (N′-VNNTVYDPLQPELDS-C′) (all JPT Peptide Technologies). All stimulations (peptide pools and single peptides) were performed at final concentrations of 1 μg/ml per peptide. For a negative control, the stimulation peptide solvent dimethyl sulfoxide diluted 1:1 in phosphate-buffered saline was used at the same concentration as in peptide-stimulated tubes. SEB/TSST-1 (1.5 and 1.0 mg/ml, respectively) (Sigma-Aldrich) and/or the CEFX Ultra SuperStim pool (1 μg/ml per peptide) (JPT Peptide Technologies) were used as positive stimulation controls. For optimized costimulation, purified anti-CD28 (clone CD28.2, BD Biosciences) was added to each stimulation at a final concentration of 1 μg/ml. Incubation was performed at 37°C at 5% CO_2_ for 16 hours in the presence of 10 μg/ml brefeldin A (Sigma-Aldrich) during the last 14 hours. CD4^+^ T cell activation was calculated as SI = percentage of CD40L^+^4-1BB^+^ CD4^+^ T cells in the stimulation divided by the percentage of CD40L^+^4-1BB^+^ CD4^+^ T cells in the unstimulated control. Dotted lines indicate an SI of 1.5 (positive with uncertainty) and 3 (definite positive).

### T cell enrichment and expansion

Activated cells were enriched from stimulated PBMCs by magnetic-activated cell sorting (MACS). Cells were stimulated with indicated PepMixes in the presence of 1 μg/ml of purified anti-CD28 (clone CD28.2, BD Biosciences) and 1 μg/ml of purified anti-CD40 (5C3, BioLegend) for 16 hours, followed by staining with anti-CD40L-APC (5C8, Miltenyi Biotec) and anti–4-1BB-PE (4B4-1, BD Biosciences). The activated cells were enriched using anti-phycoerythrin (anti-PE) MultiSort MicroBeads (Miltenyi Biotec) according to the manufacturer’s instructions. After release of anti-PE beads, a second, analogous enrichment step was performed using anti-APC MicroBeads (Miltenyi Biotec). The purity of the enriched population was routinely checked to >80% of live cells. Feeder cells were obtained from the 4-1BB-PE–negative fraction of the initial enrichment step by CD3 MicroBeads (Miltenyi Biotec) depletion and subsequent irradiation at 50 Gy. Enriched CD40L^+^4-1BB^+^ cells were co-cultured with feeder cells at a ratio of 1:1 in AB medium supplemented with 10 ng/ml of interleukin-7 (IL-7) and 10 ng/ml of IL-15 (both from Miltenyi Biotec) for 10 days, followed by 2 days of cytokine starvation. The cells were then restimulated in the presence of CD3-depleted autologous feeder cells as described above and as indicated in the figure legends. For spike glycoprotein epitope identification, restimulation was performed with the Epitope Mapping Peptide Set SARS-CoV-2 (JPT) according to the manufacturer’s instructions.

### Flow cytometry

Stimulations were stopped by incubation in 2 mM EDTA for 5 min. Surface staining was performed for 15 min in the presence of 1 mg/ml of Beriglobin (CSL Behring) with the following fluorochrome-conjugated antibodies titrated to their optimal concentrations as specified in table S3: FITC-conjugated anti-CD3 (Miltenyi Biotec), VioGreen-conjugated anti-CD4 (Miltenyi Biotec), VioBlue-conjugated anti-CD8 (Miltenyi Biotec), APC-conjugated anti-CD38 (Miltenyi Biotec), and PerCP-Vio 700–conjugated anti–HLA-DR (Miltenyi Biotec). During the last 10 min of incubation, Zombie Yellow fixable viability staining (BioLegend) was added. Fixation and permeabilization were performed with eBioscience FoxP3 fixation and PermBuffer (Invitrogen) according to the manufacturer’s protocol. Intracellular staining was performed for 30 min in the dark at room temperature with PE-conjugated anti–4-1BB (Miltenyi Biotec), PE-Vio 770–conjugated anti-CD40L (Miltenyi Biotec), Alexa Fluor 700–conjugated anti–IFN-γ (BioLegend), and Brilliant Violet 605–conjugated anti–TNF-α (BioLegend,). All samples were measured on a MACSQuant Analyzer 16 (Miltenyi Biotec). Instrument performance was monitored before every measurement with Rainbow Calibration Particles (BD Biosciences).

Anti–SARS-CoV-2 IgG and IgA ELISA specific for the S subunit 1 (S1) was performed using the commercial kits (QuantiVac for IgG, EUROIMMUN Medizinische Labordiagnostika) according to the manufacturer’s instructions and as described previously (*46*). Upper and lower cut-offs were set at 3900 and 32 for IgG, respectively, and at 0.6 and 10 for IgA, respectively.

### SARS-CoV-2 neutralization assay

Neutralization activity of SARS-CoV-2–specific antibodies was assessed with a plaque reduction neutralization test (PRNT) as described previously (*45*).

### Epitope-specific antibody ELISA

Biotinylated peptide S809-826 (Biotin-Ttds-PSKPSKR*SFIEDLLFNKV*-OH, Ttds linker=N-(3-{2-[2-(3-Amino-propoxy)-ethoxy]-ethoxy}-propyl)-succinamic acid, JPT Peptide Technologies) (400 nM) was immobilized on a 96-well streptavidin plate (Steffens Biotechnische Analysen) for 1 hour at room temperature. After blocking for 1 hour at 30°C, serum samples were diluted 1:100 and incubated for 1 hour at 30°C. Horseradish peroxidase (HRP)–coupled, anti–human IgG secondary antibody (Jackson ImmunoResearch) was diluted 1:5000 (Jackson ImmunoResearch) and added to the serum samples for 1 hour at 30°C, then HRP substrate was added (TMB, Kem-En-Tec). The reaction was stopped by adding sulfuric acid, and absorption was measured at 450 nm using a FlexStation 3.

### HLA typing and analysis

HLA typing was performed by LABType CWD assays (One Lambda, West Hills, CA, USA) based on reverse sequence-specific oligonucleotides according to the manufacturer’s instructions. Briefly, the HLA genomic region was amplified individually using locus-specific biotinylated primers for HLA-DRB1, HLA-DQA1, HLA-DQB1, HLA-DPA1, and HLA-DPB1. Amplicons were hybridized to HLA allele- and allele group–specific probes attached to Luminex beads. Complementary binding was detected by addition of R-PE–conjugated streptavidin and acquired using a FLEXMAP 3D flow analyzer (Luminex, Austin, TX, USA). HLA alleles were derived at two-field code resolution (highest probability) as referenced in the catalog of common and well-documented HLA alleles version 2.0.0 33. MHC class II binding prediction were performed using the Immune Epitope Database and Analysis Resource (www.IEDB.org) (*47**, **48*), based on the IEDB recommended method version 2.22. For the purposes of this analysis, we refer to an individual as “homozygous” if the two corresponding alleles of the same locus are identical in the first two fields.

### Homology score

For the calculation of the homology score, all possible 9-mers were generated for each respective PepMix of SARS-CoV-2. Each of the 9-mers was scored against each unique 9-mer from the proteomes of the corona viruses 229E, NL63, OC43, and HKU1 (isolates N1, N2, and N5) using the PAM30 substitution matrix. The homology score is the percentage of comparisons with a pairwise 9-mer score >30.

### Data analysis and statistics

Study data were collected and managed using REDCap electronic data capture tools hosted at Charité (*49*, *50*). Flow cytometry data were analyzed with FlowJo 10.6, and statistical analysis was conducted with GraphPad Prism 9. If not stated otherwise, data are plotted as means. *N* indicates the number of donors. *P* values were set as follows: *P < 0.05, **P < 0.01, and ***P < 0.001.

## Supplementary Material

20210831-1

## Supplementary Materials

### Other Supplementary Material for this manuscript includes the following:

MDAR Reproducibility Checklist

## Notes

View/request a protocol for this paper from *Bio-protocol*.
