# Supplementary material for: Cross-reactive CD4+ T cells enhance SARS-CoV-2 immune responses upon infection and vaccination
Source: Science. 2021 Aug 31;374(6564):eabh1823. doi: 10.1126/science.abh1823 (PMC10026850; doi:10.1126/science.abh1823)
Supplement: 20210831-1 [file science.abh1823.v1.pdf]

Cite as: L. Loyal *et al.*, *Science*
10.1126/science.abh1823 (2021).

Cross-reactive CD4⁺ T cells enhance SARS-CoV-2 immune responses upon infection and vaccination

Lucie Loyal[1,2], Julian Braun[1,2]†, Larissa Henze[1,2]†, Beate Kruse[1,2]†, Manuela Dingeldey[1,2]†, Ulf Reimer[3]†, Florian Kern[3,4]†, Tatjana Schwarz[5]†, Maike Mangold[1,2], Clara Unger[1,2], Friederike Dörfler[1], Shirin Kadler[1,6], Jennifer Rosowski[1,6], Kübrah Gürcan[1,6], Zehra Uyar-Aydin[1,6], Marco Frentsch[7,8], Florian Kurth[9,10], Karsten Schnatbaum[3], Maren Eckey[3], Stefan Hippenstiel[9], Andreas Hocke[9], Marcel A. Müller[2,5,11], Birgit Sawitzki[12], Stefan Miltenyi[13], Friedemann Paul[14,15], Marcus A. Mall[16,17], Holger Wenschuh[3], Sebastian Voigt[18,19], Christian Drosten[2,5,11], Roland Lauster[1,6]‡, Nils Lachman[20]‡, Leif-Erik Sander[9]‡, Victor M. Corman[2,5,11]‡, Jobst Röhmel[16]‡, Lil Meyer-Arndt[1,2,14,15,21]‡, Andreas Thiel[1,2]‡*, Claudia Giesecke-Thiel[22]‡*

[1]Si-M/"Der Simulierte Mensch" a Science Framework of Technische Universität Berlin and Charité - Universitätsmedizin Berlin, Berlin, Germany. [2]Charité-Universitätsmedizin Berlin, Corporate Member of Freie Universität Berlin, Humboldt-Universität zu Berlin, and Berlin Institute of Health, Berlin, Germany. [3]JPT Peptide Technologies GmbH, Berlin, Germany. [4]Brighton and Sussex Medical School, Department of Clinical and Experimental Medicine, Brighton, UK. [5]Charité - Universitätsmedizin Berlin, Institute of Virology, Berlin, Germany. [6]Medical Biotechnology, Institute for Biotechnology, Technische Universität Berlin, Berlin, Germany. [7]Charité - Universitätsmedizin Berlin, Department of Hematology, Oncology and Tumor Immunology, Berlin, Germany. [8]Berlin Institute of Health at Charité - Universitätsmedizin Berlin, Therapy-Induced Remodeling in Immuno-Oncology, Berlin, Germany. [9]Charité - Universitätsmedizin Berlin, Department of Infectious Diseases and Respiratory Medicine, Berlin, Germany. [10]Department of Tropical Medicine, Bernhard Nocht Institute for Tropical Medicine and Department of Medicine, University Medical Center Hamburg-Eppendorf, Hamburg, Germany. [11]German Centre for Infection Research (DZIF), Partner Site Charité, Berlin, Germany. [12]Charité - Universitätsmedizin Berlin, Institute of Medical Immunology, Berlin, Germany. [13]Miltenyi Biotec GmbH, Bergisch-Gladbach, Germany. [14]Experimental and Clinical Research Center, Max Delbrueck Center for Molecular Medicine and Charité - Universitätsmedizin Berlin, Berlin, Germany. [15]Charité - Universitätsmedizin Berlin, Clinical Neuroimmunology, NeuroCure Clinical Research Center, Berlin, Germany. [16]Department of Pediatric Respiratory Medicine, Immunology and Critical Care Medicine, Charité - Universitätsmedizin Berlin, Berlin, Germany. [17]German Center for Lung Research, Associated Partner, Berlin, Germany. [18]Department of Infectious Diseases, Robert Koch Institute, Berlin, Germany. [19]Institute for Virology, Universitätsklinikum Essen, Essen, Germany. [20]Charité - Universitätsmedizin Berlin, Institute for Transfusion Medicine, Tissue Typing Laboratory, Berlin, Germany. [21]Charité - Universitätsmedizin Berlin, Department of Neurology and Experimental Neurology, Berlin, Germany. [22]Max Planck Institute for Molecular Genetics, Berlin, Germany.

†These authors contributed equally to this work.

‡These authors jointly supervised this work.

***Corresponding author. Email: andreas.thiel@charite.de (A.T.); giesecke@molgen.mpg.de (C.G.-T.)**

The functional relevance of pre-existing cross-immunity to SARS-CoV-2 is a subject of intense debate. Here, we show that human endemic coronavirus (HCoV)-reactive and SARS-CoV-2-cross-reactive CD4⁺ T cells are ubiquitous but decrease with age. We identified a universal immunodominant coronavirus-specific spike peptide (S816-830) and demonstrate that pre-existing spike- and S816-830-reactive T cells were recruited into immune responses to SARS-CoV-2 infection and their frequency correlated with anti-SARS-CoV-2-S1-IgG antibodies. Spike-cross-reactive T cells were also activated after primary BNT162b2 COVID-19 mRNA vaccination displaying kinetics similar to secondary immune responses. Our results highlight the functional contribution of pre-existing spike-cross-reactive T cells in SARS-CoV-2 infection and vaccination. Cross-reactive immunity may account for the unexpectedly rapid induction of immunity following primary SARS-CoV-2 immunization and the high rate of asymptomatic/mild COVID-19 disease courses.

The majority of individuals infected with severe acute respiratory syndrome coronavirus 2 (SARS-CoV-2) experience an asymptomatic or mild course of coronavirus disease 2019 (COVID-19). However, severe or fatal disease occurs in about 5% of those infected and is primarily associated with advanced age and comorbidities such as diabetes, chronic cardiovascular, pulmonary, and kidney diseases (*1*). Given that SARS-CoV-2 is a newly emerged human pathogen, it was assumed that SARS-CoV-2 encounters an immunologically naive population. However, SARS-CoV-2 displays considerable homologies with endemic, human common cold coronaviruses (collectively referred to as "HCoV") (*2*, *3*). There is now strong evidence for cellular and humoral cross-reactivity to SARS-CoV-2 (*3*–*14*), although the role of cross-reactive immunity in SARS-CoV-2 infection is unclear (*2*, *8*, *15*, *16*). Recent HCoV infection is associated with less severe COVID-19, suggesting a protective role (*17*). A better understanding of the extent and impact of cross-immunity in

SARS-CoV-2 infection and vaccination is needed, as cognate cross-immunity may influence the efficacy of vaccination regimens.

Here we investigated the functional role of pre-existing SARS-CoV-2- and HCoV-reactive CD4⁺ T cells. The SARS-CoV-2 spike glycoprotein (spike) was the dominant target of broad T cell cross-reactivity in unexposed individuals, which decreased with age. We identified an immunodominant coronavirus peptide located within the fusion peptide domain of spike (S816-830) recognized by CD4⁺ T cells in 20% of unexposed individuals, 50 to 60% of SARS-CoV-2 convalescents, and 97% of BNT162b2-vaccinated individuals. S816-830- and spike-cross-reactive T cells were recruited into primary SARS-CoV-2 immune responses and also into BNT162b2 COVID-19 mRNA vaccination responses. Finally, upon primary vaccination, cross-reactive immunity exhibited kinetics akin to secondary immune responses. Already at an early stage of the immune response, the frequencies of pre-existing cross-reactive T cells correlated positively with functional avidity as well as with the induction and stabilization of anti-S1-IgG antibodies. Thus, cross-reactive CD4⁺ T cells accelerate the immune response in SARS-CoV-2 infection and vaccination. These findings add to the discussion surrounding single-dose vaccination of healthy adults and multiple-dose vaccination of the elderly.

Frequent and broad SARS-CoV-2-cross-reactivity in unexposed healthy donors

To determine the extent of cellular cross-reactivity to SARS-CoV-2 antigens, we stimulated CD4⁺ T cells of 60 unexposed healthy donors and 59 COVID-19 convalescents as controls (table S1) with peptide pools covering all open reading frames (ORFs) of SARS-CoV-2, referred to here as the "SARS-CoV-2 orfeome" (Fig. 1A). The SARS-CoV-2 orfeome consists of 11 ORFs, five of which (N, spike, E, M, and ORF1a/b (encoding for the non-structural proteins (NSPs) 1-16)) are also found in HCoVs 229E, OC43, NL63, and HKU1. Amino acid (aa) sequence alignment revealed discrete areas of high homology in almost all SARS-CoV-2 proteins to the corresponding proteins in HCoVs. Parts of the ORF1a/b including NSP8, NSP10, and NSP12-16 displayed the highest degree of homology and thus potential cross-reactive epitopes to all HCoVs (fig. S1A). Nevertheless, COVID-19 convalescents did not show significantly increased CD4⁺ T cell reactivity against the NSPs compared to unexposed individuals (Fig. 1A and fig. S1B). Reactivity against the combination of spike N-terminal S-I (aa residues 1-643), C-terminal S-II (aa residues 633-1273), N, and M peptide pools clearly distinguished COVID-19 convalescents from unexposed individuals irrespective of the disease course (Fig. 1, A to C). In unexposed individuals, we detected variable but low CD4⁺ T cell reactivity to virtually all SARS-CoV-2 antigens, including those exclusive to SARS-CoV-2 (not shared with HCoVs). However, the degree of aa sequence homology between HCoVs and SARS-CoV-2 proteins did not correlate with cross-reactivity (fig. S1C). Thus, apart from cognate cross-reactivity (resulting, for example, from previous exposure to similar proteins found in HCoVs), we also identified non-cognate cross-reactivity (i.e., cross-reactivity that cannot be explained by the previous exposure to similar proteins in HCoVs). Of all 30 orfeome peptide pools, the spike S-I/-II pools alone elicited T cell reactivity in all COVID-19 convalescents as well as in a subset of unexposed individuals. Since antibodies to spike induced by SARS-CoV-2 infection can neutralize the virus (*18*), and most of the recently approved SARS-CoV-2 vaccines are highly effective and include spike as the main vaccine antigen, we examined cellular immunity to spike more closely.

SARS-CoV-2 spike S-II-cross-reactive T cells decrease with age

A striking feature of SARS-CoV-2 infection is the strong correlation of higher age with disease severity. Immunosenescence is associated with a lack of newly generated T cells and, instead, the expansion of a small number of clones resulting from persistent infections, which limits the breadth and quality of T cell responsiveness (*19*, *20*). To assess the impact of age on SARS-CoV-2-(cross)-reactive T cell immunity, we examined SARS-CoV-2 spike-specific CD4⁺ T cell responses in 568 unexposed individuals and 174 COVID-19 convalescents (Fig. 2A and table S1). T cells reacting to a peptide pool representing a mixture of selected T cell epitopes from common pathogens (CEFX pool) remained relatively stable with age in both cohorts (Fig. 2A). COVID-19 convalescents displayed a significant age-associated increase in spike S-I-reactive T cells that correlated with higher disease severity in the elderly (table S1). However, in line with our previous findings (3) in unexposed individuals, T cell cross-reactivity to S-I was rare, close to the limit of detection, and remained stable albeit at low levels with increasing age. By contrast, reactivity to S-II was more frequent and generally higher in unexposed individuals which significantly decreased with increasing age (Fig. 2A). When total CD4⁺ T cells were analyzed for activation-induced IFN-γ or TNF-α expression, we did not detect any age-related changes (fig. S2A). However, among bona fide TCR-activated antigen-specific CD40L⁺4-1BB⁺ CD4⁺ T cells, IFN-γ⁺TNF-α⁺ cells decreased with age (fig. S2B). In contrast to CD40L⁺4-1BB⁺ CD4⁺ T cells, total CD40L⁺ CD4⁺ T cells, which can also be induced in part in a TCR-independent manner (*21*), did not decrease with age, consistent with the large compartment of memory T cells in older individuals (fig. S2C). Thus, elderly individuals exhibit decreased cognate cross-reactive immunity to the SARS-CoV-2 spike S-II portion, which exhibits higher homology to HCoV than the S-I portion.

Low CD3 surface expression identifies SARS-CoV-2-reactive T cells with high functional avidity ex vivo

To assess the quality of the spike-(cross-)reactive T cell response in terms of functional T cell avidity, we examined the level of CD3 surface expression in CD40L$^+$4-1BB$^+$ CD4$^+$ T cells following short-term in vitro stimulation (Fig. 2B). Strong TCR activation, characteristic of T cells with high TCR avidity, blocks recycling of the TCR–CD3 complex and can be detected by reduced CD3 surface expression (*22*), a phenomenon known as high functional avidity. Thus, cognate cross-reactivity with higher probability of high functional avidity is distinguishable from non-cognate cross-reactivity with higher probability of low functional avidity by analyzing the frequency of CD3^{lo} T cells among TCR activated CD4$^+$ T cells (fig. S3, A and B). After stimulation with spike S-I/S-II peptide pools, COVID-19 convalescents showed high frequencies of S-I- and S-II-activated CD4$^+$ T cells that largely lacked CD3 expression characteristic of cognate T cell activation (Fig. 2B). In unexposed individuals, however, the frequency of CD3^{lo} cells among S-I- and S-II-activated CD4$^+$ T cells was markedly lower. Nevertheless, especially in the younger, S-II stimulation induced higher frequencies of CD3^{lo} cells than S-I stimulation indicating that spike S-II (cross)-reactive CD4$^+$ T cells have high functional avidity (Fig. 2B). This is consistent with the high degree of homology between the C-terminal S-II portions of SARS-CoV-2 spike and HCoV spike proteins.

HCoV spike-reactive high functional avidity CD4$^+$ T cells decreases with age

We hypothesized that previous HCoV exposures induce cognate cross-reactive CD4$^+$ T cells. Therefore, we next characterized CD4$^+$ T cell immunity to HCoV spike in unexposed individuals and COVID-19 convalescents. HCoV-S-I- and -S-II-reactive CD4$^+$ T cells were more readily detectable than SARS-CoV-2 spike-specific T cells and found in 80% (S-I) and 98% (S-II) of SARS-CoV-2 unexposed individuals, respectively (Fig. 3A). Importantly, their frequency decreased with age and SARS-CoV-2 infection did not result in an increase in HCoV-S-I- or -S-II-reactive T cells. We also examined the functional avidities of HCoV-reactive CD4$^+$ T cells (Fig. 3B). High frequencies of CD3^{lo} T cells were found among both HCoV S-I- and -S-II-reactive CD4$^+$ T cells, although they significantly decreased with advancing age. Thus, a high degree of HCoV exposure in the population appears to lead to widespread cross-reactivity to SARS-CoV-2 spike. HCoV-reactive CD4$^+$ T cells frequently comprise cells with high functional avidity but significantly decrease with age.

The immunodominant peptide S816-830 is recognized by SARS-CoV-2 spike glycoprotein S-II-cross-reactive CD4$^+$ T cells

All SARS-CoV-2-cross-reactive unexposed donors showed a response against at least two (S-I) or three (S-II) HCoVs, suggesting that repeated infection with different HCoVs establishes a detectable prominent SARS-CoV-2 cross-reactive T cell pool already early in life and/or that specific T cells are directed against highly homologous sequences shared across multiple HCoVs and SARS-CoV-2 (Fig. 4A). We next examined whether HCoV spike glycoprotein-specific T cells directly cross-react to SARS-CoV-2 spike glycoprotein. Therefore, short-term CD40L$^+$4-1BB$^+$ OC43 S-I or S-II-reactive CD4$^+$ T cell lines were restimulated with OC43- or SARS-CoV-2 spike pool S-I and S-II, respectively. Six out of 18 OC43 S-II-specific T cell lines displayed cross-reactivity against SARS-CoV-2 S-II, whereas OC43 S-I-specific T cell lines lacked cross-reactivity against SARS-CoV-2 S-I (Fig. 4B). We further identified and validated two overlapping T cell–stimulating peptides (peptides 204 (SKRSFIEDLLFNKVT, aa 813-827) and 205 (FIEDLLFNKVTLADA, aa 817-831)) derived from the S-II portion, in all five donors analyzed (fig. S4, A to D). Only one donor responded to other identified peptides (peptides 188, 189, and 251) (fig. S4B). Sequence alignment revealed that S-II peptides 204 and 205 together covered the fusion peptide domain of spike, which is characterized by strong homology with HCoV (fig. S4C). By analyzing additional 15-aa peptides along the sequence covered by the peptides 204 and 205, we identified the sequence SFIEDLLFNKVTLAD (aa 816-830) as an immunodominant coronavirus peptide, hereafter referred to as S816-830 (peptide 204_3, fig. S4D). We next examined direct ex vivo T cell reactivity against S816-830 compared to a control peptide 284 (aa 1133-1147, hereafter referred to as S1133-1147) and the SARS-CoV-2 spike S-II peptide pool in 48 unexposed individuals and 22 COVID-19 convalescents. S816-830-reactive CD4$^+$ T cells were detected in 50% of convalescents and 20% of unexposed individuals with significantly higher frequencies in the former (Fig. 4C). Antibodies to the SARS-CoV-2 spike aa residues S809-826 were previously reported in COVID-19 patients but also in unexposed individuals (*23*, *24*). When we examined the sera of responders and non-responders to the S816-830 T cell assay, we detected S809-826 binding antibodies in all individuals. However, significantly higher concentrations of these antibodies were found in COVID-19 convalescents with substantially more S816-830-reactive T cells (Fig. 4D). Compared to definite non-responders (stimulation index (SI)<1.5), definite S816-830 peptide responders (SI≥3) were more frequently positive for HLA-DPB1*02:01, HLA-DPB1*04:02, and especially homozygous expression of HLA-DPB1*04:01 (Fig. 4E). Since HLA-DPA1*01:03 was found in 100% of the responders and 94.8% of the non-responders, we investigated if combinations of HLA-DPA1*01:03 and HLA-DPB1*02:01/DPB1*04:01/DPB1*04:02 were likely to present peptide S816-830 or fragments thereof. HLA-peptide-binding predictions identified excellent potential binders (fig. S4E), which was also true for the homologous S816-830 peptide in other HCoVs (fig. S4F).

Pre-existing SARS-CoV-2 S-II-cross-reactive T cells are recruited into primary SARS-CoV-2 immune responses

A still open question is whether and the extent to which SARS-CoV-2-cross-reactive T cells influence the disease course of primary SARS-CoV-2 infection. By monitoring the healthy unexposed study participants for primary SARS-CoV-2 infection, we identified 17 cases of acute primary SARS-CoV-2 infection (Fig. 5 and table S2). All 17 patients showed detectable virus titers (fig. S5A) and mild COVID-19 disease course (no hospitalization required) (table S2). Robust $CD4^+$ T cell responses specific of SARS-CoV-2 spike S-I and S-II were detected and the proportions of $HLADR^+CD38^+$ cells among $CD40L^+4\text{-}1BB^+$ $CD4^+$ T cells significantly increased at follow-up time points 1 and 2 (3-16 days), indicating their in vivo activation (Fig. 5, A and B). $CD3^{lo}$ cells substantially increased during acute primary SARS-CoV-2 infection and remained at high levels after the infection resolved (Fig. 5C). Individuals who already had spike S-II-cross-reactive $CD4^+$ T cells with a SI≥3 at baseline showed significantly higher functional avidity throughout the initiation of the T cell response (Fig. 5D). S816-830–reactive T cells increased in both frequency and in functional avidity in 10 of 17 donors after infection (Fig. 5, E and F). Notably, IgG antibodies against the S809-826 peptide were boosted as early as 3-9 days (follow-up time point 1) after the presumed infection (Fig. 5G). Anti-SARS-CoV-2-S1-IgG serum antibodies were detectable at follow-up time point 2 and peaked after day 20 in most individuals, although their kinetics and quantity varied widely (Fig. 5H). Anti-SARS-CoV-2-S1 binding antibody (IgG) units (BAU) at late time points positively correlated with S-II- but not S-I-cross-reactive T cell levels at d0 suggesting that pre-existing cross-reactive $CD4^+$ T cells enhance SARS-CoV-2-specific humoral immunity (Fig. 5I, left). Moreover, the neutralizing antibody titers also positively correlated with S-II- but not S-I-cross-reactive $CD4^+$ T cells at baseline, pointing to a protective role of cross-reactive $CD4^+$ T cells (Fig. 5I, middle and right). Finally, the frequency of HCoV-reactive $CD4^+$ T cells also increased in almost all individuals shortly after primary SARS-CoV-2 infection (Fig. 5J). There was a concomitant increase in the frequency of $CD3^{lo}$ cells (fig. S5B) and $HLADR^+CD38^+$ cells (fig. S5C) among HCoV-reactive $CD4^+$ T cells, demonstrating that pre-existing HCoV-reactive cellular immunity was activated and transiently expanded during primary SARS-CoV-2 infection. Clearly, pre-existing SARS-CoV-2 S-II-cross-reactive $CD4^+$ T cells were recruited into primary SARS-CoV-2 immune responses in healthy previously unexposed individuals. Thus, the quantity and functional avidity of pre-existing cross-reactive cellular immunity corresponds to the quality and magnitude of specific cellular and humoral anti-SARS-CoV-2 responses. It may therefore contribute to a milder course of COVID-19 by limiting viral propagation.

BNT162b2 vaccination reactivates pre-existing SARS-CoV-2 spike S-II-cross-reactive T cells

Finally, we investigated how pre-existing SARS-CoV-2 S-II-cross-reactive T cells in healthy unexposed individuals influence the course of BNT162b2 COVID-19 spike mRNA vaccine responses. We monitored baseline and follow-up humoral and T cell responses against SARS-CoV-2- and HCoV spike glycoproteins in 31 healthy adults who underwent primary (day 0) and booster vaccination (day 21) with BNT162b2. At day 21, 30 of 31 donors had detectable anti-SARS-CoV-2 S1 IgG and all donors had detectable anti-SARS-CoV-2 S1 IgA levels (Fig. 6A). Booster vaccination further increased these antibody levels. Primary vaccination also induced robust S-I- and S-II-reactive $CD4^+$ T cell responses in all individuals which were only slightly enhanced by booster vaccination (Fig. 6B). The kinetics of S-I- and S-II-reactive T cells differed in that S-II-reactive T cells showed a sharp increase from baseline to day 7 but not thereafter, whereas S-I-reactive T cells showed an additional significant increase from day 7 to day 14 (Fig. 6, B and C). This was indicative of secondary response kinetics of S-II-reactive cells and primary response kinetics of S-I-reactive cells (*22*). High-functional-avidity, $CD3^{lo}$ $CD40L^+4\text{-}1BB^+$ $CD4^+$ T cells increased more rapidly in cross-reactive donors (Fig. 6, D and E). Moreover, at day 14, S-I- and S-II-reactive $CD4^+$ T cells included high frequencies of $HLADR^+CD38^+$ cells in all but three donors indicating their recent in vivo activation (Fig. 6F). Like SARS-CoV-2–specific T cells, HCoV S-II-reactive T cells were significantly increased 7 days after primary vaccination (Fig. 6G). This was associated with an increased frequency of HCoV S-II-reactive $HLADR^+CD38^+$ T cells (Fig. 6H). Thus, cognate cross-reactive T cells were activated early in response to SARS-CoV-2 spike-specific vaccination but did not expand thereafter. All but 2 of 31 donors (94%) responded with T cells that had high functional avidity to S816-830 at days 7 and 14 (Fig. 6, I and J). S816-830–reactive T cells initially contributed up to 100% of the $CD40L^+4\text{-}1BB^+$ cells in S-II stimulations but their proportion decreased as other specificities increased during the course of the SARS-CoV-2 S-II-specific immune response (Fig. 6K). Thus, HCoV imprinting does not appear to hamper an immune response tailored to SARS-CoV-2. We observed a correlation between the S816-830-reactive T cell response and the S-II-reactive T cell response at day 0 that was even more pronounced at day 7 emphasizing the importance of the S816-830 peptide in the early stages of the of the anti-SARS-CoV-2 cellular immune response (Fig. 6L). A humoral response to S809-826 (overlapping with S816-830) was detectable upon vaccination as early as 7 days after primary vaccination (Fig. 6M) that was distinct from the slower anti-SARS-CoV-2-S1-IgG response. This supports the concept that pre-existing cross-reactive immunity mediates secondary response kinetics (*26*).

Discussion

The functional relevance of pre-existing cognate cross-immunity to SARS-CoV-2 is a subject of intense debate. Non-cognate cross-reactivity has been reported, but appears to play a minor role, compared to HCoV-mediated cognate cross-reactivity (*16*, *26*). A recent HCoV infection is associated with a less severe course of COVID-19 (*17*). Interestingly, more than 90% of the population is HCoV-seropositive. Thus, a large proportion of the population might benefit from cross-reactive humoral immunity (*27*, *28*). However, pre-pandemic serum from PCR-validated HCoV-positive individuals contains neutralizing antibodies against all HCoVs but not SARS-CoV-2 (*27*). In a subsequent study, only low spike-specific cross-reactive antibody activity was detected in just 5 of 34 donors with recent HCoV infection and in just 1 of 31 donors without recent HCoV infection, indicating that humoral cross-immunity is weak and decays rapidly (12). Finally, although infection with SARS-CoV-2 increases the prevalence of antibodies against seasonal HCoVs, they do not provide protection, which highlights the role of cross-reactive cellular immunity (*9*, *27*, *28*).

Recently T cells, cross-reactive to several SARS-CoV-2 antigens, were identified in unexposed individuals using predicted peptides individually (*4*, *5*) or as megapools (*8*, *29*). Our work reveals significant cross-reactivity of ORF1a/b-encoded proteins, but also shows that most of the anti-SARS-CoV-2 reactivity is directed against the spike, N, and M proteins. We further demonstrate that the magnitude and quality of SARS-CoV-2 cross-reactivity and HCoV-reactivity declines with age. The failure of an aging immune system to maintain HCoV-induced SARS-CoV-2-cross-reactive T cells along with a smaller pool of naïve T cells, which can be recruited into SARS-CoV-2-specific responses (*20*), may contribute to the increased susceptibility of elderly to severe COVID-19. Our results show that HCoV-specific, SARS-CoV-2-cross-reactive T cells contribute to SARS-CoV-2 immune responses upon infection and vaccination. Additionally, such cognate cross-reactivity correlates with a rapid cellular and enhanced humoral response, which may both favor mild disease courses. The sequential administration of different haptens sharing the same carrier to mice induces pre-existing T cell help for the second hapten, leading to more efficient B cell recruitment in secondary immunization (*30*). Accordingly, B cells recognizing SARS-CoV-2 may benefit from HCoV-reactive T cells cross-reacting with SARS-CoV-2 peptides. Further studies in mice showed that increasing the numbers of antigen-specific T cells at the onset of the immune response also increased B cell activation and proliferation. Moreover, the presence of cognate T cell help during viral infection promotes germinal center formation, which is required for fast and high-affinity antibody generation (*30*–*32*). Since the early induction of SARS-CoV-2 T cell reactivity has been associated with rapid viral clearance and mild disease (*33*), cross-reactive T cells that enhance the immune response to SARS-CoV-2 may well serve as a correlate of immune protection against severe COVID-19 disease courses (*34*, *35*).

Upon BNT162b2 vaccination, we observed immune responses that exceeded the response to actual SARS-CoV-2 infection in terms of spike-specific T cell and antibody levels. Responses to S-II, unlike responses to the non-cross-reactive S-I, however, displayed kinetics reminiscent of a secondary immune response (*25*, *26*). These observations may provide an explanation for the results of large studies showing high efficacy of SARS-CoV-2 vaccines. Protection levels against SARS-CoV-2 infection have been reported to be greater than 75% as early as 15–28 days after primary vaccination with BNT162b2 (*36*). In addition, just one dose of the BNT162b2 or the Astra Zeneca ChAdOx1 vaccine reduced the risk of hospitalization by 85% and 94%, respectively, at days 28-34 post primary vaccination, pointing to an unusually high vaccine efficacy for a primary vaccination (*36*). Similarly, a single-shot vaccination based on AdV26 adenovirus-encoded modified spike protein from Johnson & Johnson has been reported to have a vaccine efficacy of 66% was recently approved by the FDA and the EMA (*37*, *38*). Our results may provide an immunological explanation for the reported high efficacies. Conversely, in the elderly, with waning HCoV T cell reactivity and thus reduced SARS-CoV-2 T cell cross-reactivity, additional booster vaccinations may be critical (*39*).

The immunodominant cross-reactive peptide (S816-830) identified here is located within the highly conserved spike fusion peptide domain downstream of the S2′ cleavage site (*40*). We demonstrate that S816-830-reactive T cells are efficiently recruited into the SARS-CoV-2 response in the majority of infected and nearly all vaccinated individuals. Previous reports have also shown that specific antibodies against this region are generated after SARS-CoV-2 infection and vaccination with BNT162b2 (*23*, *24*). In addition, it has been proposed that antibodies specific to the S2 portion of spike possess neutralizing activity and may be involved in the early induction of protection before SARS-CoV-2-S1-specifc antibodies emerged (*28*, *41*–*43*). In summary, the S816-830 peptide may serve as a conserved universal coronavirus target in the S2 portion of spike for both B cells and T cells. Enhancing the immune response to S816-830 may induce efficient protection and should be a focus of future studies.

Materials and Methods

Study participants

This study was approved by the Institutional Review board of the Charité (EA/152/20). Written informed consent was obtained from all included participants (*44*) and the study

was conducted in agreement with the declaration of Helsinki. Participants who had tested positive for SARS-CoV-2 RNA (RT-qPCR from nasopharyngeal swabs) were classified as convalescent donors. All donors were assessed for age, gender, BMI, comorbidities, and medications (table S1). Convalescent donors were subclassified according to their symptoms into WHO severity grades and information about hospitalization or admission to an intensive care unit (ICU) is given in table S1. Day of infection was set as day −3 prior to reported symptom onset. Measurement day post symptom onset is indicated in the graphs or table S1. Study participants who reported symptoms typical for a SARS-CoV-2 infection were RT-qPCR tested for virus RNA and positive donors were enrolled for follow-up measurements. Details of the follow-up cohort (age, gender, comorbidities, symptoms, measurement timepoints post symptom onset) are provided in table S2.

Coronavirus RT-qPCR

RNA was extracted using the MagNA Pure 96 system and the MagNA Pure Viral NA Small Volume Kit (Roche, Germany). RNA extraction was performed from a 200-µl swab dilution (swab suspended in 4.3 ml of Cobas PCR Media, Roche), eluted in 100 µl of elution buffer. Coronavirus detection using 5 µl of the RNA eluate was based on two genomic targets (E- and N gene, TIB Molbiol, Berlin, Germany). An in-vitro transcribed RNA of equine arteritis virus was used as an internal RT and PCR control. SARS-CoV-2 was quantified using the E-gene target and by applying calibration curves and using serial diluted photometrically quantified in-vitro transcribed RNA as described before (*45*). All RT-qPCRs were performed using a LightCycler 480 II (Roche).

Blood and serum sampling and PBMC isolation

Whole blood was collected in lithium heparin tubes for peripheral blood mononuclear cells (PBMC) isolation and SSTII advance (all Vacutainer®, BD) tubes for serology. SSTII advance tubes were centrifuged for 10 min at 1000*g* prior to removing serum. Serum aliquots were frozen at −20°C until further use. PBMCs were isolated by gradient density centrifugation according to the manufacturer's instructions (Leucosep tubes, Greiner; Biocoll, Bio&SELL).

Ex vivo T cell stimulation

Freshly isolated PBMC were cultivated at a concentration of 5×10^6 PBMC/ml in AB-medium containing RPMI 1640 medium (Gibco) supplemented with 10% heat inactivated AB serum (Pan Biotech), 100 U/ml of penicillin (Biochrom), and 0.1 mg/ml of streptomycin (Biochrom). Stimulations were conducted with PepMix™ overlapping peptide pools (15-aa length with 11-aa overlaps, JPT Peptide Technologies) covering the proteins of interest, including the entire SARS-CoV-2 orfeome i.e., the spike glycoprotein (S), NCAP-1 (N), VEMP-1 (E), VME-1 (M), AP3A (ORF3a), NS6, NS7A, NS7B, NS8, ORF9B, ORF10, Y14 (ORF9c), the ORF1a/b proteins (NSP01, NSP02, NSP03a, NSP03b, NSP04, NSP05, NSP06, NSP07, NSP08, NSP09, NSP10, NSP11, NSP12, NSP13, NSP14, NSP15, and NSP16), as well as the spike glycoproteins of HCoV-229E, HCoV-OC43, HCoV-NL63, and HCoV-HKU1 (all JPT Peptide Technologies). Single peptide stimulations were conducted with the following peptides: 204 (N′-SKRSFIEDLLFNKVT-C′), 204_1 (N′-KRSFIEDLLFNKVTL-C′), 204_2 (N′-RSFIEDLLFNKVTLA-C′), 204_3 (N′-SFIEDLLFNKVTLAD-C′), 205 (N′-FIEDLLFNKVTLADA-C′), and the control peptide 284 (N′-VNNTVYDPLQPELDS-C′) (all JPT Peptide Technologies). All stimulations (peptide pools and single peptides) were performed at final concentrations of 1 µg/ml per peptide. For negative control the stimulation peptide solvent DMSO diluted 1:1 in PBS was used at the same concentration as in peptide-stimulated tubes. SEB/TSST-1 (1.5 mg/ml and 1.0 mg/ml, respectively) (Sigma) and/or the CEFX Ultra SuperStim pool (1 µg/ml per peptide) (JPT Peptide Technologies) were used as positive stimulation controls. For optimized costimulation, purified anti-CD28 (clone CD28.2, BD Biosciences) was added to each stimulation at a final concentration of 1 µg/ml. Incubation was performed at 37°C, 5% CO_2 for 16 hours in the presence of 10 µg/ml brefeldin A (Sigma-Aldrich) during the last 14 hours. $CD4^+$ T cell activation was calculated as a stimulation index (SI)=% of $CD40L^+4\text{-}1BB^+$ $CD4^+$ T cells in the stimulation / % of $CD40L^+4\text{-}1BB^+$ $CD4^+$ T cells in the unstimulated control. Dotted lines indicate an SI of 1.5 (positive with uncertainty) and 3 (definite positive).

T cell enrichment and expansion

Activated cells were enriched from stimulated PBMCs by magnetic cell sorting (MACS). Cells were stimulated with indicated PepMixes in the presence of 1 µg/ml of purified anti-CD28 (clone CD28.2, BD Biosciences) and 1 µg/ml of purified anti-CD40 (5C3, Biolegend) for 16 hours followed by staining with anti-CD40L-APC (5C8, Miltenyi) and anti-4-1BB-PE (4B4-1, BD). The activated cells were enriched using anti-PE MultiSort MicroBeads (Miltenyi) according to the manufacturer's instructions. After release of anti-PE beads a second, analogous enrichment step was performed using anti-APC MicroBeads (Miltenyi). The purity of the enriched population was routinely checked to >80% of live cells. Feeder cells were obtained from the 4-1BB-PE negative fraction of the initial enrichment step by CD3 MicroBeads (Miltenyi) depletion and subsequent irradiation at 50 Gy. Enriched $CD40L^+4\text{-}1BB^+$ cells were co-cultured with feeder cells at a ratio of 1:1 in AB-medium supplemented with 10 ng/ml of IL-7 and 10 ng/ml of IL-15 (both from Miltenyi) for 10 days followed by 2 days of cytokine starvation. They were

then restimulated in the presence of CD3-depleted autologous feeder cells as described above and indicated in the figure legends. For spike glycoprotein epitope identification, restimulation was performed with the Epitope Mapping Peptide Set SARS-CoV-2 (JPT) according to the manufacturer's instructions.

Flow cytometry

Stimulations were stopped by incubation in 2 mM EDTA for 5 min. Surface staining was performed for 15 min in the presence of 1 mg/ml of Beriglobin (CSL Behring) with the following fluorochrome-conjugated antibodies titrated to their optimal concentrations as specified in table S3: FITC-conjugated anti-CD3 (Miltenyi), VioGreen-conjugated anti-CD4 (Miltenyi), VioBlue-conjugated anti-CD8 (Miltenyi), APC-conjugated anti-CD38 (Miltenyi), and PerCP-Vio 700-conjugated anti-HLA-DR (Miltenyi). During the last 10 min of incubation, Zombie Yellow fixable viability staining (Biolegend) was added. Fixation and permeabilization were performed with eBioscience™ FoxP3 fixation and PermBuffer (Invitrogen) according to the manufacturer's protocol. Intracellular staining was carried out for 30 min in the dark at room temperature with PE-conjugated anti-4-1BB (Miltenyi), PE-Vio 770-conjugated anti-CD40L (Miltenyi), Alexa Fluor 700-conjugated anti-IFN-γ (Biolegend), and Brilliant Violet 605-conjugated anti-TNF-α (Biolegend,). All samples were measured on a MACSQuant® Analyzer 16 (Miltenyi). Instrument performance was monitored prior to every measurement with Rainbow Calibration Particles (BD Biosciences).

Anti-SARS-CoV-2 IgG and IgA ELISA specific for the S subunit 1 (S1) was performed using the commercial kits ((QuantiVac for IgG), EUROIMMUN Medizinische Labordiagnostika AG) according to the manufacturer's instructions and as described previously (*46*). Upper and lower cut-offs were set at 3900 and 32 for IgG, respectively, and at 0.6 and 10 for IgA, respectively.

SARS-CoV-2 neutralization assay

Neutralization activity of SARS-CoV-2 specific antibodies was assessed with a plaque reduction neutralization test (PRNT) as described before (*39*).

Epitope specific antibody ELISA

Biotinylated peptide S809-826 (Biotin-Ttds-PSKPSKR*SFIEDLLFNKV*-OH, Ttds linker=N-(3-{2-[2-(3-Amino-propoxy)-ethoxy]-ethoxy}-propyl)-succinamic acid, JPT Peptide Technologies) (400 nM) was immobilized on a 96-well Streptavidin plate (Steffens Biotechnische Analysen GmbH) for 1 hour at RT. After blocking (1 hour, 30°C) serum samples were diluted 1:100 and incubated for 1 hour at 30°C. HRP-coupled, anti-human-IgG secondary antibody (Jackson Immunoresearch) was diluted 1:5000 (Jackson Immunoresearch) and added to the serum samples for 1 hour at 30°C, then HRP substrate was added (TMB, Kem-En-Tec). The reaction was stopped by adding sulfuric acid and absorption was measured at 450 nm using a FlexStation 3.

HLA typing and analysis

HLA typing was performed by LABType® CWD assays (One Lambda, West Hills, CA, USA) based on reverse sequence-specific oligonucleotides (rSSO) according to the manufacturer's instructions. Briefly, the HLA genomic region was amplified individually using locus-specific biotinylated primers for HLA-DRB1, HLA-DQA1, HLA-DQB1, HLA-DPA1, and HLA-DPB1. Amplicons were hybridized to HLA allele- and allele-group-specific probes attached to Luminex® beads. Complementary binding was detected by addition of R-phycoerythrin-conjugated streptavidin and acquired using a FLEXMAP 3D flow analyzer (Luminex, Austin, TX, USA). HLA alleles were derived at two-field code resolution (highest probability) as referenced in the catalog of common and well-documented HLA alleles version 2.0.0 33. MHC class II binding prediction were performed using the IEDB Analysis Resource (www.IEDB.org) (*47, 48*), based on the IEDB recommended method version 2.22. For the purpose of this analysis, we refer to an individual as "homozygous" if the two corresponding alleles of the same locus are identical in the first two fields.

Homology score

For the calculation of the homology score, all possible 9-mers were generated for each respective PepMix of SARS-CoV-2. Each of the 9-mers was scored against each unique 9-mer from the proteomes of the corona viruses 229E, NL63, OC43, and HKU1 (isolates N1, N2, and N5) using the PAM30 substitution matrix. The homology score is the percentage of comparisons with a pairwise 9-mer score above 30.

Data analysis and statistics

Study data were collected and managed using REDCap electronic data capture tools hosted at Charité (*49*, *50*). Flow cytometry data were analyzed with FlowJo 10.6 (FlowJo LLC) and statistical analysis conducted with GraphPad Prism 9. If not stated otherwise, data are plotted as means. *N* indicates the number of donors. *P*-values were set as follows: $*P < 0.05$, $**P < 0.01$, and $***P < 0.001$.

REFERENCES AND NOTES

1. E. J. Williamson, A. J. Walker, K. Bhaskaran, S. Bacon, C. Bates, C. E. Morton, H. J. Curtis, A. Mehrkar, D. Evans, P. Inglesby, J. Cockburn, H. I. McDonald, B. MacKenna, L. Tomlinson, I. J. Douglas, C. T. Rentsch, R. Mathur, A. Y. S. Wong, R. Grieve, D. Harrison, H. Forbes, A. Schultze, R. Croker, J. Parry, F. Hester, S. Harper, R. Perera, S. J. W. Evans, L. Smeeth, B. Goldacre, Factors associated with COVID-19-related death using OpenSAFELY. *Nature* **584**, 430–436 (2020). doi:10.1038/s41586-020-2521-4 Medline
2. U. Stervbo, S. Rahmann, T. Roch, T. H. Westhoff, N. Babel, Epitope similarity cannot explain the pre-formed T cell immunity towards structural SARS-CoV-2 proteins. *Sci. Rep.* **10**, 18995 (2020). doi:10.1038/s41598-020-75972-z Medline
3. J. Braun, L. Loyal, M. Frentsch, D. Wendisch, P. Georg, F. Kurth, S. Hippenstiel, M.

Dingeldey, B. Kruse, F. Fauchere, E. Baysal, M. Mangold, L. Henze, R. Lauster, M. A. Mall, K. Beyer, J. Röhmel, S. Voigt, J. Schmitz, S. Miltenyi, I. Demuth, M. A. Müller, A. Hocke, M. Witzenrath, N. Suttorp, F. Kern, U. Reimer, H. Wenschuh, C. Drosten, V. M. Corman, C. Giesecke-Thiel, L. E. Sander, A. Thiel, SARS-CoV-2-reactive T cells in healthy donors and patients with COVID-19. *Nature* **587**, 270–274 (2020). doi:10.1038/s41586-020-2598-9 Medline

4. A. Grifoni, D. Weiskopf, S. I. Ramirez, J. Mateus, J. M. Dan, C. R. Moderbacher, S. A. Rawlings, A. Sutherland, L. Premkumar, R. S. Jadi, D. Marrama, A. M. de Silva, A. Frazier, A. F. Carlin, J. A. Greenbaum, B. Peters, F. Krammer, D. M. Smith, S. Crotty, A. Sette, Targets of T cell responses to SARS-CoV-2 coronavirus in humans with COVID-19 disease and unexposed individuals. *Cell* **181**, 1489–1501.e15 (2020). doi:10.1016/j.cell.2020.05.015 Medline

5. A. Nelde, T. Bilich, J. S. Heitmann, Y. Maringer, H. R. Salih, M. Roerden, M. Lübke, J. Bauer, J. Rieth, M. Wacker, A. Peter, S. Hörber, B. Traenkle, P. D. Kaiser, U. Rothbauer, M. Becker, D. Junker, G. Krause, M. Strengert, N. Schneiderhan-Marra, M. F. Templin, T. O. Joos, D. J. Kowalewski, V. Stos-Zweifel, M. Fehr, A. Rabsteyn, V. Mirakaj, J. Karbach, E. Jäger, M. Graf, L.-C. Gruber, D. Rachfalski, B. Preuß, I. Hagelstein, M. Märklin, T. Bakchoul, C. Gouttefangeas, O. Kohlbacher, R. Klein, S. Stevanović, H.-G. Rammensee, J. S. Walz, SARS-CoV-2-derived peptides define heterologous and COVID-19-induced T cell recognition. *Nat. Immunol.* **22**, 74–85 (2021). doi:10.1038/s41590-020-00808-x Medline

6. N. Le Bert, A. T. Tan, K. Kunasegaran, C. Y. L. Tham, M. Hafezi, A. Chia, M. H. Y. Chng, M. Lin, N. Tan, M. Linster, W. N. Chia, M. I.-C. Chen, L.-F. Wang, E. E. Ooi, S. Kalimuddin, P. A. Tambyah, J. G.-H. Low, Y.-J. Tan, A. Bertoletti, SARS-CoV-2-specific T cell immunity in cases of COVID-19 and SARS, and uninfected controls. *Nature* **584**, 457–462 (2020). doi:10.1038/s41586-020-2550-z Medline

7. J. Mateus, A. Grifoni, A. Tarke, J. Sidney, S. I. Ramirez, J. M. Dan, Z. C. Burger, S. A. Rawlings, D. M. Smith, E. Phillips, S. Mallal, M. Lammers, P. Rubiro, L. Quiambao, A. Sutherland, E. D. Yu, R. da Silva Antunes, J. Greenbaum, A. Frazier, A. J. Markmann, L. Premkumar, A. de Silva, B. Peters, S. Crotty, A. Sette, D. Weiskopf, Selective and cross-reactive SARS-CoV-2 T cell epitopes in unexposed humans. *Science* **370**, 89–94 (2020). doi:10.1126/science.abd3871 Medline

8. P. Bacher, E. Rosati, D. Esser, G. R. Martini, C. Saggau, E. Schiminsky, J. Dargvainiene, I. Schröder, I. Wieters, Y. Khodamoradi, F. Eberhardt, M. J. G. T. Vehreschild, H. Neb, M. Sonntagbauer, C. Conrad, F. Tran, P. Rosenstiel, R. Markewitz, K.-P. Wandinger, M. Augustin, J. Rybniker, M. Kochanek, F. Leypoldt, O. A. Cornely, P. Koehler, A. Franke, A. Scheffold, Low-avidity CD4+ T cell responses to SARS-CoV-2 in unexposed individuals and humans with severe COVID-19. *Immunity* **53**, 1258–1271.e5 (2020). doi:10.1016/j.immuni.2020.11.016 Medline

9. E. M. Anderson, E. C. Goodwin, A. Verma, C. P. Arevalo, M. J. Bolton, M. E. Weirick, S. Gouma, C. M. McAllister, S. R. Christensen, J. Weaver, P. Hicks, T. B. Manzoni, O. Oniyide, H. Ramage, D. Mathew, A. E. Baxter, D. A. Oldridge, A. R. Greenplate, J. E. Wu, C. Alanio, K. D'Andrea, O. Kuthuru, J. Dougherty, A. Pattekar, J. Kim, N. Han, S. A. Apostolidis, A. C. Huang, L. A. Vella, L. Kuri-Cervantes, M. B. Pampena, M. R. Betts, E. J. Wherry, N. J. Meyer, S. Cherry, P. Bates, D. J. Rader, S. E. Hensley; UPenn COVID Processing Unit, Seasonal human coronavirus antibodies are boosted upon SARS-CoV-2 infection but not associated with protection. *Cell* **184**, 1858–1864.e10 (2021). doi:10.1016/j.cell.2021.02.010 Medline

10. N. Mishra, X. Huang, S. Joshi, C. Guo, J. Ng, R. Thakkar, Y. Wu, X. Dong, Q. Li, R. S. Pinapati, E. Sullivan, A. Caciula, R. Tokarz, T. Briese, J. Lu, W. I. Lipkin, Immunoreactive peptide maps of SARS-CoV-2. *Commun. Biol.* **4**, 225 (2021). doi:10.1038/s42003-021-01743-9

11. A. P. Ferretti, T. Kula, Y. Wang, D. M. V. Nguyen, A. Weinheimer, G. S. Dunlap, Q. Xu, N. Nabilsi, C. R. Perullo, A. W. Cristofaro, H. J. Whitton, A. Virbasius, K. J. Olivier Jr., L. R. Buckner, A. T. Alistar, E. D. Whitman, S. A. Bertino, S. Chattopadhyay, G. MacBeath, Unbiased screens show CD8+ T cells of COVID-19 patients recognize shared epitopes in SARS-CoV-2 that largely reside outside the Spike protein. *Immunity* **53**, 1095–1107.e3 (2020). doi:10.1016/j.immuni.2020.10.006 Medline

12. K. W. Ng, N. Faulkner, G. H. Cornish, A. Rosa, R. Harvey, S. Hussain, R. Ulferts, C. Earl, A. G. Wrobel, D. J. Benton, C. Roustan, W. Bolland, R. Thompson, A. Agua-Doce, P. Hobson, J. Heaney, H. Rickman, S. Paraskevopoulou, C. F. Houlihan, K. Thomson, E. Sanchez, G. Y. Shin, M. J. Spyer, D. Joshi, N. O'Reilly, P. A. Walker, S. Kjaer, A. Riddell, C. Moore, B. R. Jebson, M. Wilkinson, L. R. Marshall, E. C. Rosser, A. Radziszewska, H. Peckham, C. Ciurtin, L. R. Wedderburn, R. Beale, C. Swanton, S. Gandhi, B. Stockinger, J. McCauley, S. J. Gamblin, L. E. McCoy, P. Cherepanov, E. Nastouli, G. Kassiotis, Preexisting and de novo humoral immunity to SARS-CoV-2 in humans. *Science* **370**, 1339–1343 (2020). doi:10.1126/science.abe1107 Medline

13. D. Weiskopf, K. S. Schmitz, M. P. Raadsen, A. Grifoni, N. M. A. Okba, H. Endeman, J. P. C. van den Akker, R. Molenkamp, M. P. G. Koopmans, E. C. M. van Gorp, B. L. Haagmans, R. L. de Swart, A. Sette, R. D. de Vries, Phenotype and kinetics of SARS-CoV-2-specific T cells in COVID-19 patients with acute respiratory distress syndrome. *Sci. Immunol.* **5**, eabd2071 (2020). doi:10.1126/sciimmunol.abd2071 Medline

14. T. Sekine, A. Perez-Potti, O. Rivera-Ballesteros, K. Strålin, J.-B. Gorin, A. Olsson, S. Llewellyn-Lacey, H. Kamal, G. Bogdanovic, S. Muschiol, D. J. Wullimann, T. Kammann, J. Emgård, T. Parrot, E. Folkesson, O. Rooyackers, L. I. Eriksson, J.-I. Henter, A. Sönnerborg, T. Allander, J. Albert, M. Nielsen, J. Klingström, S. Gredmark-Russ, N. K. Björkström, J. K. Sandberg, D. A. Price, H.-G. Ljunggren, S. Aleman, M. Buggert, Robust T cell immunity in convalescent individuals with asymptomatic or mild COVID-19. *Cell* **183**, 158–168.e14 (2020). doi:10.1016/j.cell.2020.08.017 Medline

15. A. A. Lehmann, G. A. Kirchenbaum, T. Zhang, P. A. Reche, P. V. Lehmann, Deconvoluting the T cell response to SARS-CoV-2: Specificity versus chance and cognate cross-reactivity. *Front. Immunol.* **12**, 635942 (2021). doi:10.3389/fimmu.2021.635942 Medline

16. W. S. Lee, A. K. Wheatley, S. J. Kent, B. J. DeKosky, Antibody-dependent enhancement and SARS-CoV-2 vaccines and therapies. *Nat. Microbiol.* **5**, 1185–1191 (2020). doi:10.1038/s41564-020-00789-5 Medline

17. M. Sagar, K. Reifler, M. Rossi, N. S. Miller, P. Sinha, L. F. White, J. P. Mizgerd, Recent endemic coronavirus infection is associated with less-severe COVID-19. *J. Clin. Invest.* **131**, e143380 (2021). doi:10.1172/JCI143380 Medline

18. L. Liu, P. Wang, M. S. Nair, J. Yu, M. Rapp, Q. Wang, Y. Luo, J. F.-W. Chan, V. Sahi, A. Figueroa, X. V. Guo, G. Cerutti, J. Bimela, J. Gorman, T. Zhou, Z. Chen, K.-Y. Yuen, P. D. Kwong, J. G. Sodroski, M. T. Yin, Z. Sheng, Y. Huang, L. Shapiro, D. D. Ho, Potent neutralizing antibodies against multiple epitopes on SARS-CoV-2 spike. *Nature* **584**, 450–456 (2020). doi:10.1038/s41586-020-2571-7 Medline

19. D. L. Farber, N. A. Yudanin, N. P. Restifo, Human memory T cells: Generation, compartmentalization and homeostasis. *Nat. Rev. Immunol.* **14**, 24–35 (2014). doi:10.1038/nri3567 Medline

20. S. Kohler, A. Thiel, Life after the thymus: CD31+ and CD31- human naive CD4+ T-cell subsets. *Blood* **113**, 769–774 (2009). doi:10.1182/blood-2008-02-139154 Medline

21. A. Sattler, U. Wagner, M. Rossol, J. Sieper, P. Wu, A. Krause, W. A. Schmidt, S. Radmer, S. Kohler, C. Romagnani, A. Thiel, Cytokine-induced human IFN-γ-secreting effector-memory Th cells in chronic autoimmune inflammation. *Blood* **113**, 1948–1956 (2009). doi:10.1182/blood-2008-02-139147 Medline

22. H. Liu, M. Rhodes, D. L. Wiest, D. A. A. Vignali, On the dynamics of TCR:CD3 complex cell surface expression and downmodulation. *Immunity* **13**, 665–675 (2000). doi:10.1016/S1074-7613(00)00066-2 Medline

23. P. Holenya, P. J. Lange, U. Reimer, W. Woltersdorf, T. Panterodt, M. Glas, M. Wasner, M. Eckey, M. Drosch, J.-M. Hollidt, M. Naumann, F. Kern, H. Wenschuh, R. Lange, K. Schnatbaum, F. F. Bier, Peptide microarray based analysis of antibody responses to SARS-CoV-2 identifies unique epitopes with potential for diagnostic test development. medRxiv 2020.11.24.20216663 [Preprint]. 27 November 2020. https://doi.org/10.1101/2020.11.24.20216663.

24. E. Shrock, E. Fujimura, T. Kula, R. T. Timms, I.-H. Lee, Y. Leng, M. L. Robinson, B. M. Sie, M. Z. Li, Y. Chen, J. Logue, A. Zuiani, D. McCulloch, F. J. N. Lelis, S. Henson, D. R. Monaco, M. Travers, S. Habibi, W. A. Clarke, P. Caturegli, O. Laeyendecker, A. Piechocka-Trocha, J. Z. Li, A. Khatri, H. Y. Chu, A.-C. Villani, K. Kays, M. B. Goldberg, N. Hacohen, M. R. Filbin, X. G. Yu, B. D. Walker, D. R. Wesemann, H. B. Larman, J. A. Lederer, S. J. Elledge; MGH COVID-19 Collection & Processing Team, Viral epitope profiling of COVID-19 patients reveals cross-reactivity and correlates of severity. *Science* **370**, eabd4250 (2020). doi:10.1126/science.abd4250 Medline

25. A. A. Minervina, M. V. Pogorelyy, E. A. Komech, V. K. Karnaukhov, P. Bacher, E. Rosati, A. Franke, D. M. Chudakov, I. Z. Mamedov, Y. B. Lebedev, T. Mora, A. M. Walczak, Primary and secondary anti-viral response captured by the dynamics and phenotype of individual T cell clones. *eLife* **9**, e53704 (2020). doi:10.7554/eLife.53704 Medline

26. K. Fink, Origin and function of circulating plasmablasts during acute viral infections. *Front. Immunol.* **3**, 78 (2012). doi:10.3389/fimmu.2012.00078 Medline

27. D. Poston, Y. Weisblum, H. Wise, K. Templeton, S. Jenks, T. Hatziioannou, P. Bieniasz, Absence of severe acute respiratory syndrome coronavirus 2 neutralizing activity in prepandemic sera from individuals with recent seasonal coronavirus infection. *Clin. Infect. Dis.* ciaa1803 (2020). doi:10.1093/cid/ciaa1803 Medline
28. W. R. Morgenlander, S. N. Henson, D. R. Monaco, A. Chen, K. Littlefield, E. M. Bloch, E. Fujimura, I. Ruczinski, A. R. Crowley, H. Natarajan, S. E. Butler, J. A. Weiner, M. Z. Li, T. S. Bonny, S. E. Benner, A. Balagopal, D. Sullivan, S. Shoham, T. C. Quinn, S. H. Eshleman, A. Casadevall, A. D. Redd, O. Laeyendecker, M. E. Ackerman, A. Pekosz, S. J. Elledge, M. Robinson, A. A. R. Tobian, H. B. Larman, Antibody responses to endemic coronaviruses modulate COVID-19 convalescent plasma functionality. *J. Clin. Invest.* **131**, e146927 (2021). doi:10.1172/JCI146927 Medline
29. A. Tarke, J. Sidney, C. K. Kidd, J. M. Dan, S. I. Ramirez, E. D. Yu, J. Mateus, R. da Silva Antunes, E. Moore, P. Rubiro, N. Methot, E. Phillips, S. Mallal, A. Frazier, S. A. Rawlings, J. A. Greenbaum, B. Peters, D. M. Smith, S. Crotty, D. Weiskopf, A. Grifoni, A. Sette, Comprehensive analysis of T cell immunodominance and immunoprevalence of SARS-CoV-2 epitopes in COVID-19 cases. *Cell Rep. Med.* **2**, 100204 (2021). doi:10.1016/j.xcrm.2021.100204 Medline
30. T. A. Schwickert, B. Alabyev, T. Manser, M. C. Nussenzweig, Germinal center reutilization by newly activated B cells. *J. Exp. Med.* **206**, 2907–2914 (2009). doi:10.1084/jem.20091225 Medline
31. T. A. Schwickert, G. D. Victora, D. R. Fooksman, A. O. Kamphorst, M. R. Mugnier, A. D. Gitlin, M. L. Dustin, M. C. Nussenzweig, A dynamic T cell-limited checkpoint regulates affinity-dependent B cell entry into the germinal center. *J. Exp. Med.* **208**, 1243–1252 (2011). doi:10.1084/jem.20102477 Medline
32. K. Fink, N. Manjarrez-Orduño, A. Schildknecht, J. Weber, B. M. Senn, R. M. Zinkernagel, H. Hengartner, B cell activation state-governed formation of germinal centers following viral infection. *J. Immunol.* **179**, 5877–5885 (2007). doi:10.4049/jimmunol.179.9.5877 Medline
33. A. T. Tan, M. Linster, C. W. Tan, N. Le Bert, W. N. Chia, K. Kunasegaran, Y. Zhuang, C. Y. L. Tham, A. Chia, G. J. D. Smith, B. Young, S. Kalimuddin, J. G. H. Low, D. Lye, L.-F. Wang, A. Bertoletti, Early induction of functional SARS-CoV-2-specific T cells associates with rapid viral clearance and mild disease in COVID-19 patients. *Cell Rep.* **34**, 108728 (2021). doi:10.1016/j.celrep.2021.108728 Medline
34. M. Lipsitch, Y. H. Grad, A. Sette, S. Crotty, Cross-reactive memory T cells and herd immunity to SARS-CoV-2. *Nat. Rev. Immunol.* **20**, 709–713 (2020). doi:10.1038/s41577-020-00460-4 Medline
35. A. T. Huang, B. Garcia-Carreras, M. D. T. Hitchings, B. Yang, L. C. Katzelnick, S. M. Rattigan, B. A. Borgert, C. A. Moreno, B. D. Solomon, L. Trimmer-Smith, V. Etienne, I. Rodriguez-Barraquer, J. Lessler, H. Salje, D. S. Burke, A. Wesolowski, D. A. T. Cummings, A systematic review of antibody mediated immunity to coronaviruses: Kinetics, correlates of protection, and association with severity. *Nat. Commun.* **11**, 4704 (2020). doi:10.1038/s41467-020-18450-4 Medline
36. S. Amit, G. Regev-Yochay, A. Afek, Y. Kreiss, E. Leshem, Early rate reductions of SARS-CoV-2 infection and COVID-19 in BNT162b2 vaccine recipients. *Lancet* **397**, 875–877 (2021). doi:10.1016/S0140-6736(21)00448-7 Medline
37. G. Alter, J. Yu, J. Liu, A. Chandrashekar, E. N. Borducchi, L. H. Tostanoski, K. McMahan, C. Jacob-Dolan, D. R. Martinez, A. Chang, T. Anioke, M. Lifton, J. Nkolola, K. E. Stephenson, C. Atyeo, S. Shin, P. Fields, I. Kaplan, H. Robins, F. Amanat, F. Krammer, R. S. Baric, M. Le Gars, J. Sadoff, A. M. de Groot, D. Heerwegh, F. Struyf, M. Douoguih, J. van Hoof, H. Schuitemaker, D. H. Barouch, Immunogenicity of Ad26.COV2.S vaccine against SARS-CoV-2 variants in humans. *Nature* **596**, 268–272 (2021). doi:10.1038/s41586-021-03681-2 Medline
38. J. Sadoff, G. Gray, A. Vandebosch, V. Cárdenas, G. Shukarev, B. Grinsztejn, P. A. Goepfert, C. Truyers, H. Fennema, B. Spiessens, K. Offergeld, G. Scheper, K. L. Taylor, M. L. Robb, J. Treanor, D. H. Barouch, J. Stoddard, M. F. Ryser, M. A. Marovich, K. M. Neuzil, L. Corey, N. Cauwenberghs, T. Tanner, K. Hardt, J. Ruiz-Guiñazú, M. Le Gars, H. Schuitemaker, J. Van Hoof, F. Struyf, M. Douoguih; ENSEMBLE Study Group, Safety and efficacy of single-dose Ad26.COV2.S vaccine against Covid-19. *N. Engl. J. Med.* **384**, 2187–2201 (2021). doi:10.1056/NEJMoa2101544 Medline
39. J. L. Bernal, N. Andrews, C. Gower, J. Stowe, C. Robertson, E. Tessier, R. Simmons, S. Cottrell, R. Roberts, M. O'Doherty, K. Brown, C. Cameron, D. Stockton, J. McMenamin, M. Ramsay, Early effectiveness of COVID-19 vaccination with BNT162b2 mRNA vaccine and ChAdOx1 adenovirus vector vaccine on symptomatic disease, hospitalisations and mortality in older adults in England. MedRxiv 2021.03.01.21252652 [Preprint]. 2 March 2021. https://doi.org/10.1101/2021.03.01.21252652.
40. C. M. Saad-Roy, S. E. Morris, C. J. E. Metcalf, M. J. Mina, R. E. Baker, J. Farrar, E. C. Holmes, O. G. Pybus, A. L. Graham, S. A. Levin, B. T. Grenfell, C. E. Wagner, Epidemiological and evolutionary considerations of SARS-CoV-2 vaccine dosing regimes. *Science* **372**, 363–370 (2021). doi:10.1126/science.abg8663 Medline
41. A. L. Lai, J. K. Millet, S. Daniel, J. H. Freed, G. R. Whittaker, The SARS-CoV fusion peptide forms an extended bipartite fusion platform that perturbs membrane order in a calcium-dependent manner. *J. Mol. Biol.* **429**, 3875–3892 (2017). doi:10.1016/j.jmb.2017.10.017 Medline
42. C. M. Poh, G. Carissimo, B. Wang, S. N. Amrun, C. Y.-P. Lee, R. S.-L. Chee, S.-W. Fong, N. K.-W. Yeo, W.-H. Lee, A. Torres-Ruesta, Y.-S. Leo, M. I.-C. Chen, S.-Y. Tan, L. Y. A. Chai, S. Kalimuddin, S. S. G. Kheng, S.-Y. Thien, B. E. Young, D. C. Lye, B. J. Hanson, C.-I. Wang, L. Renia, L. F. P. Ng, Two linear epitopes on the SARS-CoV-2 spike protein that elicit neutralising antibodies in COVID-19 patients. *Nat. Commun.* **11**, 2806 (2020). doi:10.1038/s41467-020-16638-2 Medline
43. S. Ravichandran, Y. Lee, G. Grubbs, E. M. Coyle, L. Klenow, O. Akasaka, M. Koga, E. Adachi, M. Saito, I. Nakachi, T. Ogura, R. Baba, M. Ito, M. Kiso, A. Yasuhara, S. Yamada, Y. Sakai-Tagawa, K. Iwatsuki-Horimoto, M. Imai, S. Yamayoshi, H. Yotsuyanagi, Y. Kawaoka, S. Khurana, Longitudinal antibody repertoire in "mild" versus "severe" COVID-19 patients reveals immune markers associated with disease severity and resolution. *Sci. Adv.* **7**, eabf2467 (2021). doi:10.1126/sciadv.abf2467 Medline
44. F. Kurth, M. Roennefarth, C. Thibeault, V. M. Corman, H. Müller-Redetzky, M. Mittermaier, C. Ruwwe-Glösenkamp, K. M. Heim, A. Krannich, S. Zvorc, S. Schmidt, L. Kretzler, C. Dang-Heine, M. Rose, M. Hummel, A. Hocke, R. H. Hübner, B. Opitz, M. A. Mall, J. Röhmel, U. Landmesser, B. Pieske, S. Knauss, M. Endres, J. Spranger, F. P. Mockenhaupt, F. Tacke, S. Treskatsch, S. Angermair, B. Siegmund, C. Spies, S. Weber-Carstens, K.-U. Eckardt, D. Schürmann, A. Uhrig, M. S. Stegemann, T. Zoller, C. Drosten, N. Suttorp, M. Witzenrath, S. Hippenstiel, C. von Kalle, L. E. Sander, Studying the pathophysiology of coronavirus disease 2019: A protocol for the Berlin prospective COVID-19 patient cohort (Pa-COVID-19). *Infection* **48**, 619–626 (2020). doi:10.1007/s15010-020-01464-x Medline
45. R. Wölfel, V. M. Corman, W. Guggemos, M. Seilmaier, S. Zange, M. A. Müller, D. Niemeyer, T. C. Jones, P. Vollmar, C. Rothe, M. Hoelscher, T. Bleicker, S. Brünink, J. Schneider, R. Ehmann, K. Zwirglmaier, C. Drosten, C. Wendtner, Virological assessment of hospitalized patients with COVID-2019. *Nature* **581**, 465–469 (2020). doi:10.1038/s41586-020-2196-x Medline
46. N. M. A. Okba, M. A. Müller, W. Li, C. Wang, C. H. GeurtsvanKessel, V. M. Corman, M. M. Lamers, R. S. Sikkema, E. de Bruin, F. D. Chandler, Y. Yazdanpanah, Q. Le Hingrat, D. Descamps, N. Houhou-Fidouh, C. B. E. M. Reusken, B.-J. Bosch, C. Drosten, M. P. G. Koopmans, B. L. Haagmans, Severe acute respiratory syndrome coronavirus 2–specific antibody responses in coronavirus disease patients. *Emerg. Infect. Dis.* **26**, 1478–1488 (2020). doi:10.3201/eid2607.200841 Medline
47. P. Wang, J. Sidney, C. Dow, B. Mothé, A. Sette, B. Peters, A systematic assessment of MHC class II peptide binding predictions and evaluation of a consensus approach. *PLOS Comput. Biol.* **4**, e1000048 (2008). doi:10.1371/journal.pcbi.1000048 Medline
48. P. Wang, J. Sidney, Y. Kim, A. Sette, O. Lund, M. Nielsen, B. Peters, Peptide binding predictions for HLA DR, DP and DQ molecules. *BMC Bioinformatics* **11**, 568 (2010). doi:10.1186/1471-2105-11-568 Medline
49. P. A. Harris, R. Taylor, B. L. Minor, V. Elliott, M. Fernandez, L. O'Neal, L. McLeod, G. Delacqua, F. Delacqua, J. Kirby, S. N. Duda; REDCap Consortium, The REDCap consortium: Building an international community of software platform partners. *J. Biomed. Inform.* **95**, 103208 (2019). doi:10.1016/j.jbi.2019.103208 Medline
50. P. A. Harris, R. Taylor, R. Thielke, J. Payne, N. Gonzalez, J. G. Conde, Research electronic data capture (REDCap)—A metadata-driven methodology and workflow process for providing translational research informatics support. *J. Biomed. Inform.* **42**, 377–381 (2009). doi:10.1016/j.jbi.2008.08.010 Medline

ACKNOWLEDGMENTS

The authors thank S. Kolling and the Charité Corona Cross (CCC) Study Group associates J. Schlotterbeck, E. Baysal, T. Panne, F. Legler, M. Girod, B. Bohnen, T. Nguyen, J. Schmitz, R. Klemencic, L. Hintze, N. Avinc, P. Resch, A. Maraj, A. Farghaly, P. Schulz, K. Du, N. Matuschewski, K.Gutwenger, I. Katsianas, Z. Cheng, A. Braginets, G. Janach, and A. Bogdanski for their contributions to donor recruitment, sample processing and measurement and F. Gutsch, T. Bleicker, J. Tesch, P. Tscheak, M. L. Schmidt, and J. Riege for their support. We thank P. Jordan for the illustration of the graphical abstract figure. We thank U. Klein for the critical reading of the manuscript. **Funding:** This work was funded by the Federal Ministery of Health through a resolution of the German Bundestag (Charité Corona Cross (CCC) and Charité Corona Cross 2.0 (CCC 2.0)). Parts of the work was funded by the German Research Foundation through KFO339 to J.B. and SFB-TR84 projects A4, B6, C8, C10 to L.E.S., A.H., S.H. and B8 to M.A.Ma.; by the European Union's Horizon 2020 research and innovation program through project RECOVER (GA101003589) to C.D.; the German Ministry of Research through the projects RAPID (01KI1723A) to C.D., A.H., S.H., DZIF (301-4-7-01.703) to C.D., DZL (82DZL0098B1) to M.A. Ma, VARIPath (01KI2021) to V.M.C., RECAST to B.S., M.A.Ma., J.Rö., V.M.C., L.E.S. and NaFoUniMedCovid19 - COVIM, FKZ: 01KX2021 to L.E.S., C.D., V.M.C. and A.T. **Author contributions**: Conceptualization: L.L., C.G.T., A.T.; Data curation: L.L., J.B., L.H., V.M.C., T.S.; Formal Analysis: L.L., J.B., L.H., F.Ke.; Funding acquisition: A.T.; Investigation: L.L., L.H., J.B.; Resources: B.K., M.D., U.R., F.Ke., M.M., C.U., T.S., F.D., R.L., S.K., J.Ro., K.G., Z.U.-A., M.F., F.Ku., K.S., M.E., S.H., A.H., B.S., S.M., F.P., M.A.Ma., H.W., S.V., M.A.Mu., C.D., N.L., L.M.-A., L.E.S., V.M.C., J.Rö.; Visualization: L.L., J.B., U.R., F.Ke.; Writing: L.L., F.Ke., A.T., C.G.T. **Competing interests**: The authors L.L., J.B., L.H. and A.T. filed a patent application regarding the usage of flow cytometrically assessed CD3 down-regulation as indicator for functional TCR avidity. L.L. and A.T. filed a patent application regarding S816-830 as dominant cross-reactive T cell epitope. The authors K.S., M.E., U.R. and F.Ke. are employees, H.W. is chief executive officer of JPT. F.K. is co-inventor and co-owner of a patent describing the use of protein-spanning, overlapping peptide pools for detecting antigen-specific T-cells as applied in this study (WO2001063286A2, now expired). F.K. also holds a part-time position as research fellow with the Department of Laboratory Medicine, Medizinische Hochschule Brandenburg, Neuruppin/Germany. S.M. is chief executive officer of Miltenyi Biotec. M.A.Mu. and V.M.C. are named together with Euroimmun GmbH on a patent application filed recently regarding the diagnostic of SARS-CoV-2 by antibody testing. F.P. reports research grants and speaker honoraria from Bayer, Teva, Genzyme, Merck, MedImmune and Novartis unrelated to this work and is member of the steering committee of the OCTIMS study (Novartis). J.R. reports personal fees or grants from Vertex Pharmaceuticals during the conduct of the study. All other authors declare that they have no competing interests. **Data and materials availability**: All data are available in the main text or in the supplementary materials. This work is licensed under a Creative Commons Attribution 4.0 International (CC BY 4.0) license, which permits unrestricted use, distribution, and reproduction in any medium, provided the original work is properly cited. To view a copy of this license, visit https://creativecommons.org/licenses/by/4.0/. This license does not apply to figures/photos/artwork or other content included in the article that is credited to a third party; obtain authorization from the rights holder before using such material.

SUPPLEMENTARY MATERIALS

science.sciencemag.org/cgi/content/full/science.abh1823/DC1
Figs. S1 to S6
Tables S1 to S3
MDAR Reproducibility Checklist

14 March 2021; accepted 24 August 2021
Published online 31 August 2021
10.1126/science.abh1823

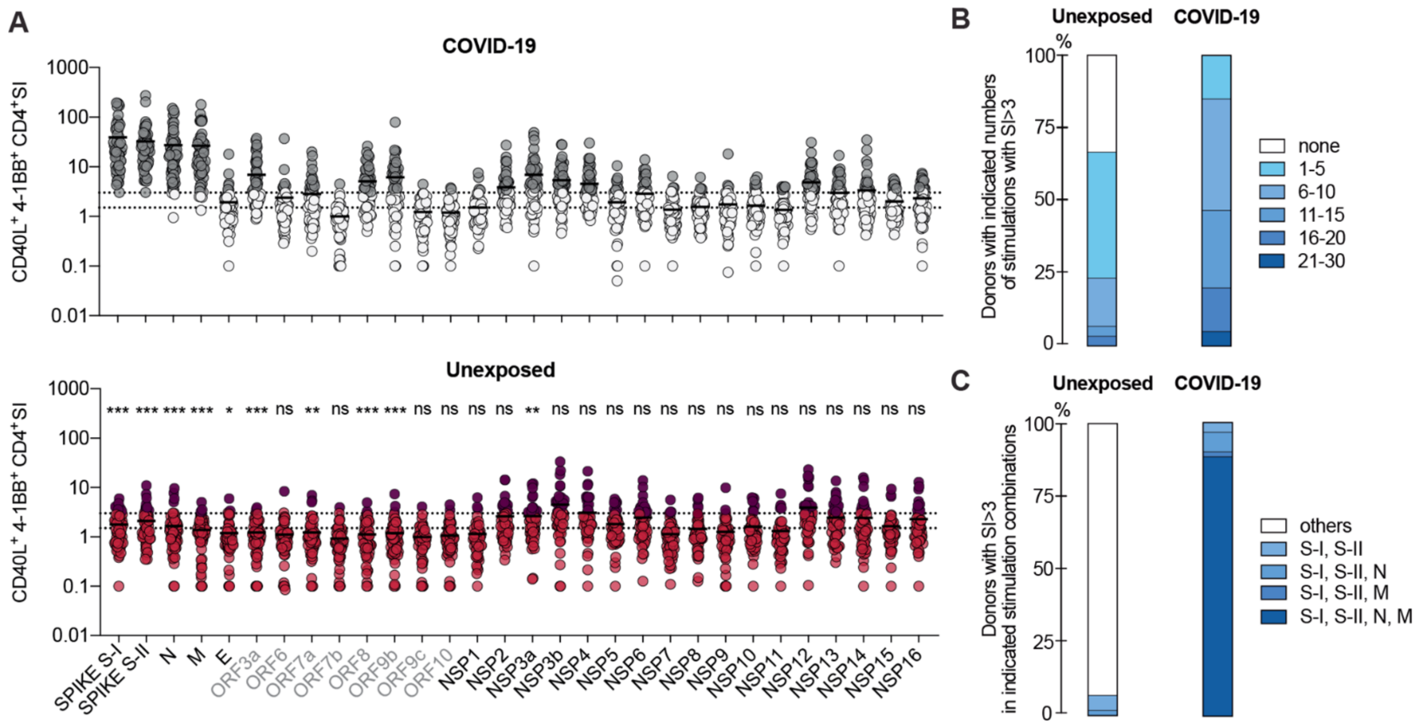

Fig. 1. CD4⁺ T cell cross-reactivity against the SARS-CoV-2 orfeome. (**A**) Ex vivo stimulation of PBMCs from COVID-19 convalescent patients (upper panel, *n*=59) and unexposed individuals (lower panel, *n*=60). The percentage of CD40L⁺4-1BB⁺ CD4⁺ T cells among stimulated PBMC was divided by the percentage of these cells among unstimulated PBMC to determine the stimulation index (SI) shown on the *y*-axis. The SARS-CoV-2-orfeome peptide pools used for stimulation are shown below the lower panel. Gray labels highlight proteins exclusive for SARS-CoV-2 (i.e., those not shared with HCoVs). Gray (COVID-19) or red circles (unexposed) identify donors with an SI≥3. Dotted lines indicate an SI of 1.5 and 3. Statistically significant differences between COVID-19 convalescents and unexposed groups (with respect to each peptide pool) are indicated above the lower panel (**P*<0.05, ***P*<0.01, ****P*<0.001, and ns=not significant at *P*>0.05; unpaired Student's *t* test). (**B**) Bars show the proportions of individuals with the indicated number of SARS-CoV-2-orfeome peptide pool stimulations with an SI≥3. (**C**) Proportions of individuals with an SI≥3 for each stimulation in each indicated stimulation combination.

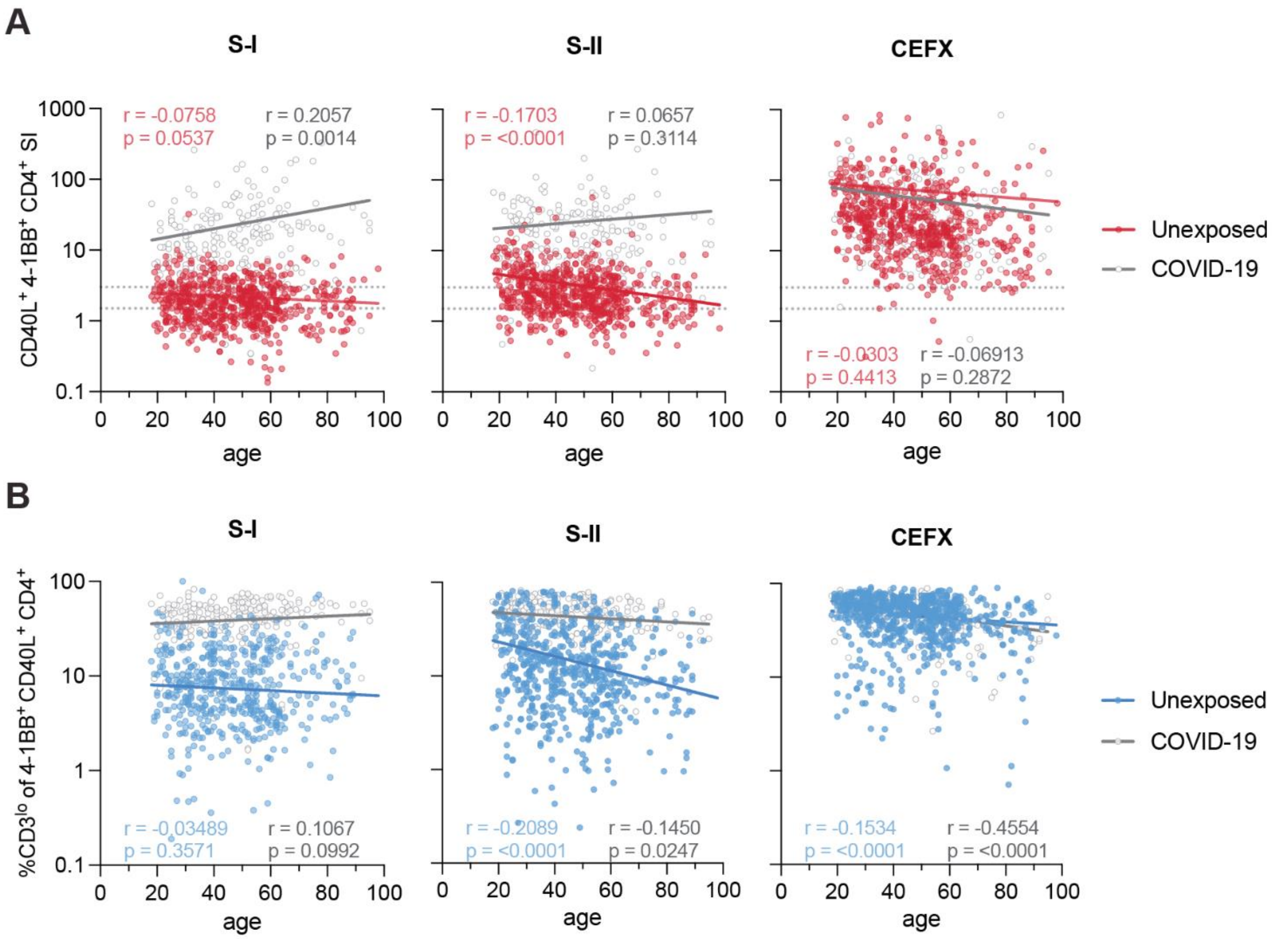

Fig. 2. The magnitude of SARS-CoV-2-cross-reactivity decreases with age. (**A**) Scatter plots show the SI (CD40L⁺41BB⁺ CD4⁺ T cells) among PBMCs stimulated with SARS-CoV-2 S-I, SARS-CoV-2 S-II, or CEFX (known T cell-stimulating peptides from CMV, EBV, flu and other common pathogens) plotted against age in *n*=568 unexposed donors and *n*=174 COVID-19 convalescents. Dotted lines indicate an SI of 1.5 and 3. (**B**) Frequencies of CD3^{lo} cells among S-I-, S-II-, or CEFX-reactive CD40L^{+}4-1BB^{+} CD4^{+} T cells over age. CD3^{lo} frequencies are shown for T cell responses with an SI≥1.5. Regression lines denote linear regression on age in each group. The corresponding Pearson correlation coefficients are shown.

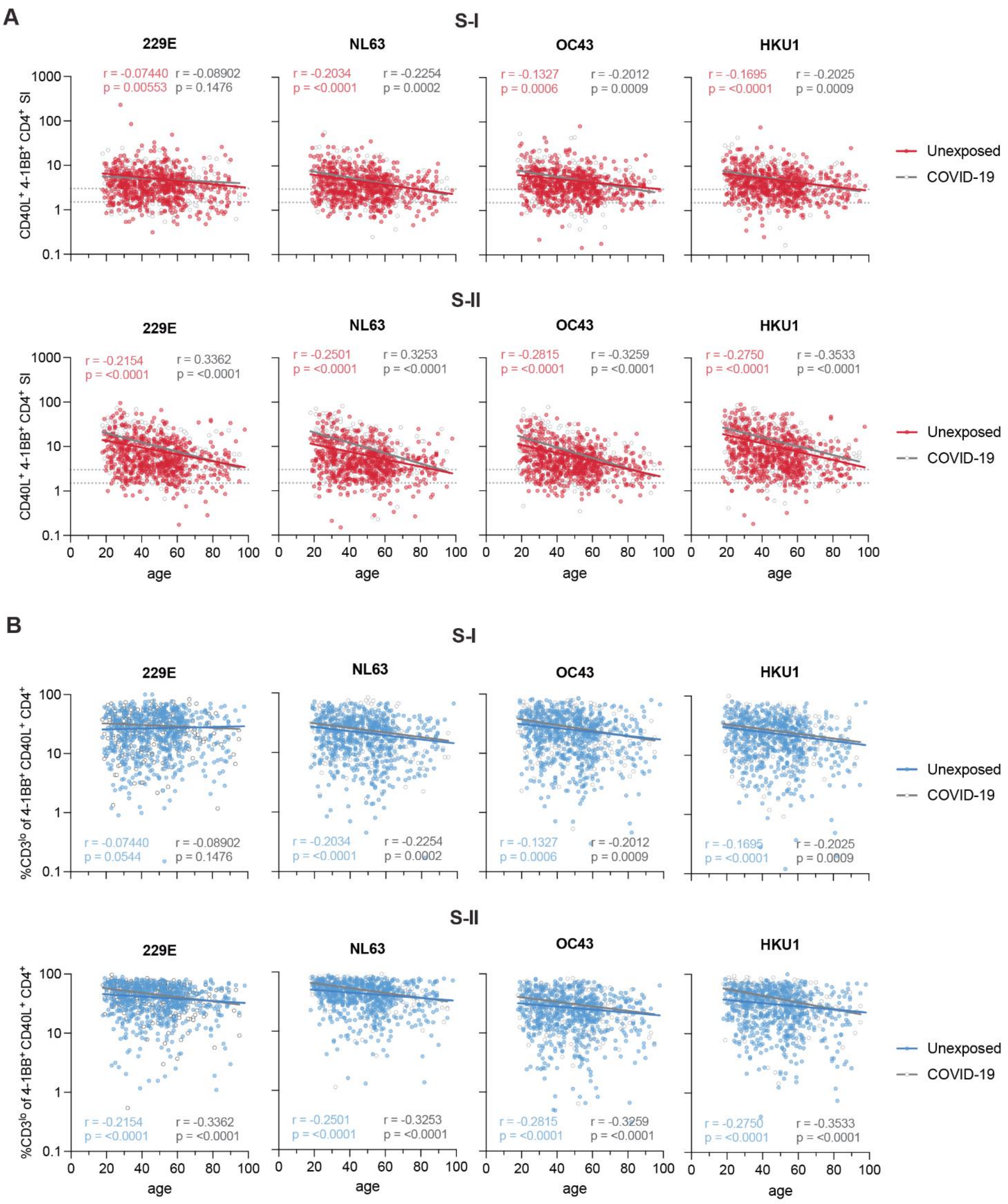

Fig. 3. High-functional-avidity T cells specific for spike S-II from HCoVs decrease with age. (**A**) Scatter plots show the SI of CD40L⁺4-1BB⁺ CD4⁺ T cells in unexposed (*n*=568) and COVID-19 convalescents (*n*=174) after PBMC stimulation with HCoV (229E, NL63, OC43, and HKU1) spike glycoprotein S-I or S-II peptide pools plotted against age. Dotted lines indicate an SI of 1.5 and 3. (**B**) Frequencies of CD3^{lo} cells in CD40L⁺4-1BB⁺ CD4⁺ T cells from unexposed and COVID-19 convalescents plotted against age. CD3^{lo} frequencies are shown for T cell responses with an SI≥1.5. Regression lines denote linear regression on age in each group; the corresponding Pearson correlation coefficients are shown.

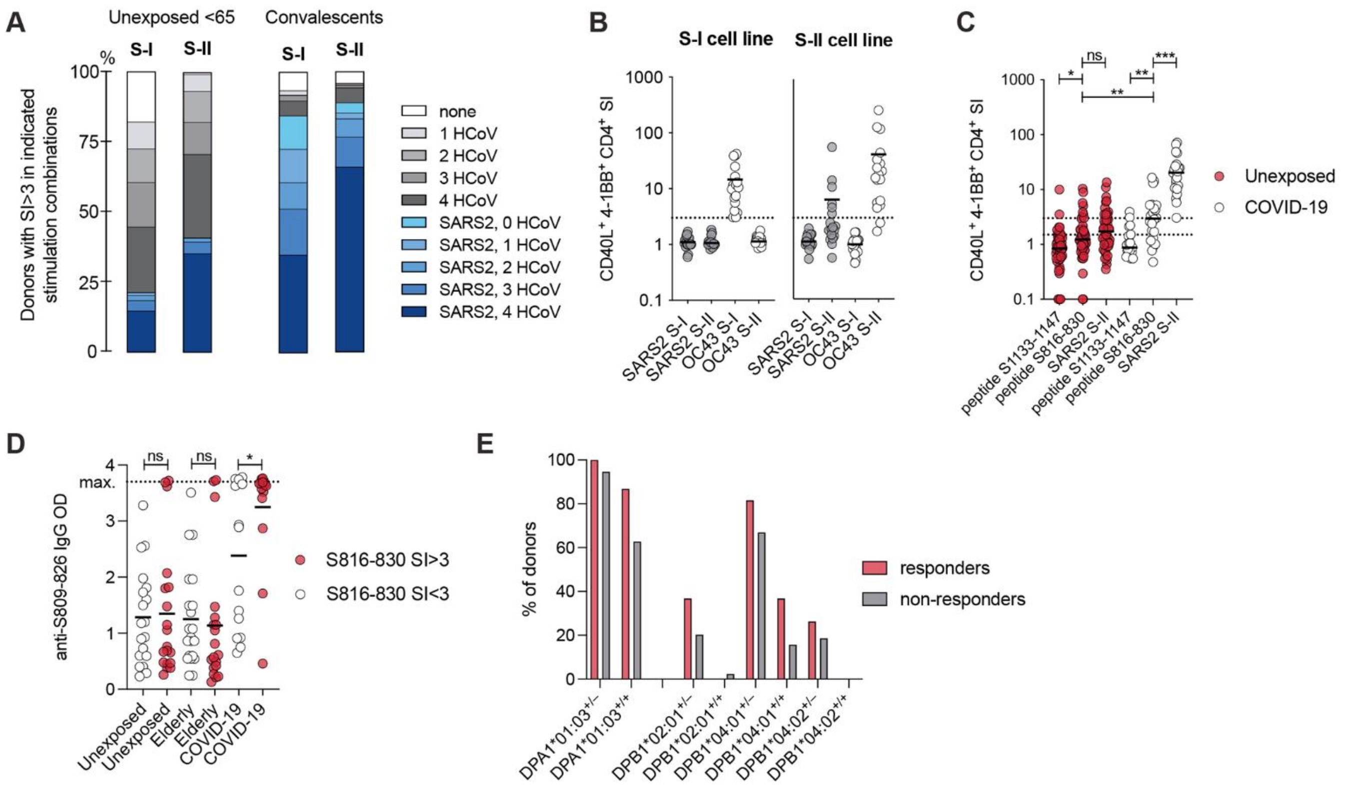

Fig. 4. Peptide S816-830 constitutes an immunodominant epitope of SARS-CoV-2 T cell cross-reactivity. (**A**) Bars show the proportions of unexposed individuals aged below 65 (n=491) and COVID-19 convalescents (n=174) with S-I or S-II-specific T cell responses to HCoV and/or SARS-CoV-2 with an SI≥3. (**B**) Plots show the SI (CD40L$^+$4-1BB$^+$ CD4$^+$ T cells) of short-term T cell lines derived from OC43 S-I- and S-II-reactive primary T cells after restimulation with autologous antigen presenting cells (APCs) in the presence of OC43 or SARS-CoV-2 spike glycoprotein pools S-I and S-II. The dotted line indicates an SI of 3. (**C**) The SIs of CD40L$^+$4-1BB$^+$ CD4$^+$ T cells from unexposed (n=48) or COVID-19 convalescents (n=22) after stimulation with the single peptide 204_3 (S816-830), the control single peptide 284 (S1133-1147) or the S-II peptide pool are shown. (**D**) Levels (optical density, OD) of anti-S809-826-peptide IgG (ELISA) in unexposed young (<65 years) and elderly (>65 years) individuals as well as COVID-19 convalescents. ELISA plates were coated with an 18 amino acid-peptide overlapping by 11 amino acids with S816-30. Serum was diluted 1:100. (**E**) Bars show the frequencies of common class-II HLA alleles in definite S816-830 responders (SI≥3) and definite non-responders (SI<1.5), n=308, $^{+/+}$=homozygous, $^{+/-}$=heterozygous. *P<0.05, **P<0.01, ***P<0.001 and ns for P>0.05 (Student's t test).

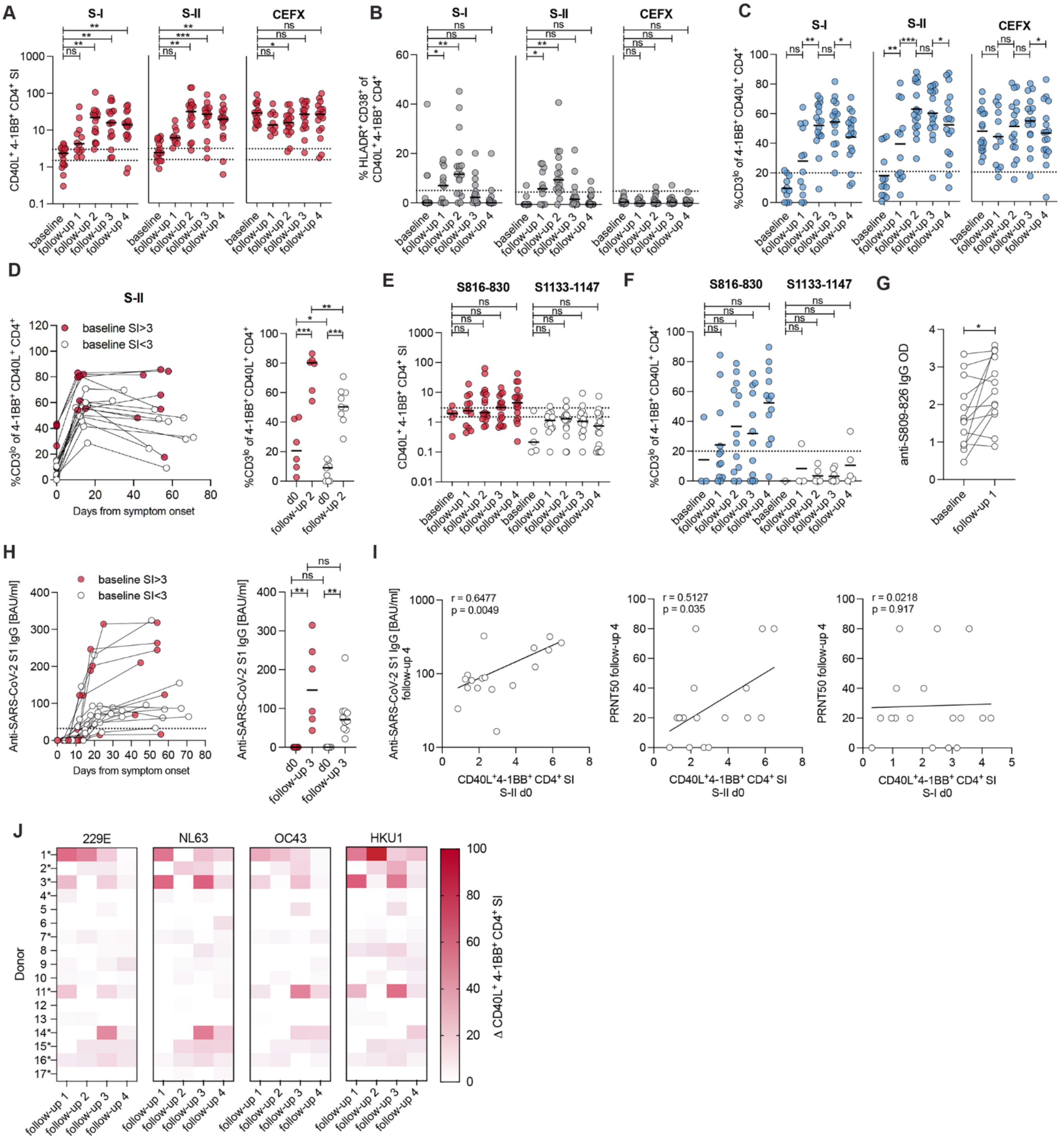
A
S-I
S-II
CEFX
CD40L+ 4-1BB+ CD4+ SI
baseline
follow-up 1
follow-up 2
follow-up 3
follow-up 4
B
% HLADR+ CD38+ of CD40L+ 4-1BB+ CD4+
C
%CD3lo of 4-1BB+ CD40L+ CD4+
D
baseline SI>3
baseline SI<3
%CD3lo of 4-1BB+ CD40L+ CD4+
Days from symptom onset
d0
E
S816-830
S1133-1147
F
G
anti-S809-826 IgG OD
H
Anti-SARS-CoV-2 S1 IgG [BAU/ml]
I
r = 0.6477
p = 0.0049
Anti-SARS-CoV-2 S1 IgG [BAU/ml] follow-up 4
CD40L+4-1BB+ CD4+ SI
S-II d0
r = 0.5127
p = 0.035
PRNT50 follow-up 4
r = 0.0218
p = 0.917
S-I d0
J
229E
NL63
OC43
HKU1
Donor
Δ CD40L+ 4-1BB+ CD4+ SI

Fig. 5. HCoV-specific SARS-CoV-2-cross-reactive T cells are recruited into the primary SARS-CoV-2 infection response. (**A**-**C**) SI of CD40L$^+$4-1BB$^+$ CD4$^+$ T cells (A), frequencies of HLADR$^+$CD38$^+$ cells (B), and frequencies of CD3^{lo} cells (C) among CD40L$^+$4-1BB$^+$ CD4$^+$ T cells after stimulation with SARS-CoV-2 S-I, S-II, and CEFX peptide pools of donors prior to infection (baseline) and at four different follow-up time points (table S2) post symptom onset. CD3^{lo} frequencies are shown for T cell responses with an SI≥1.5 only. (**D**) Changes to CD3^{lo} frequencies among CD40L$^+$4-1BB$^+$ CD4$^+$ T cells between baseline, follow-up 2 (10-16 days) and follow-up 4 (29-71 days after symptom onset) (left plot), and statistics (right plot) for baseline and follow-up measurement time point 2 in cross-reactive donors (baseline SI≥3, red circles) and non-cross-reactive donors (baseline SI<3, white circles). (**E**) SI of CD40L$^+$4-1BB$^+$ CD4$^+$ T cells and (**F**) frequency of CD3^{lo} of CD40L$^+$4-1BB$^+$ CD4$^+$ T cells after stimulation with peptide S816-830 or control peptide S1133-1147. CD3^{lo} frequencies are shown for T cell responses with an SI≥1.5. (**G**) Levels (optical density, OD) of anti-S809-826-peptide IgG (ELISA) at baseline and follow-up time point 1 (3-9 days) post symptom onset. ELISA plates were coated with an 18 amino acid peptide overlapping by 11 amino acids with S816-830. (**H**) Anti-S1-IgG binding antibody units (BAU) in cross-reactive (baseline SI≥3, red circles) and non-cross-reactive donors (baseline SI<3, white circles) were plotted against time (left), and compared between baseline and follow-up 3 (right). (**I**) Scatter plots show the relationship between anti-SARS-CoV-2 S1 IgG antibody levels (OD) at follow-up 4 and the SI of CD40L$^+$4-1BB$^+$ CD4$^+$ T cells upon S-II stimulation at baseline (left), the relationship between neutralizing antibody titers (PRNT50) at follow-up 4 and the SI of CD40L$^+$4-1BB$^+$ CD4$^+$ T cells upon S-II stimulation (left) or S-I stimulation (right) at baseline. (**J**) Heat map showing the delta (Δ) SI of CD40L$^+$4-1BB$^+$ CD4$^+$ T cells after stimulation with S-II pools of indicated HCoVs. Δ represents the change of the parameter at the given time point relative to baseline (i.e., white depicts no increase). "*" indicates S816-830 peptide responders. A, B, E, F: *$P<0.05$, **$P<0.01$, ***$P<0.001$ and ns for $P>0.05$ (repeated measure one-way-ANOVA with Dunnett`s correction). C, G: *$P<0.05$, **$P<0.01$, ***$P<0.001$ and ns for $P>0.05$ (paired Student's *t* test). D, H: *$P<0.05$, **$P<0.01$, ***$P<0.001$ and ns for $P>0.05$ (Student's *t* test). E: ns for $P>0.05$ (paired Student's *t* test). I, J: Pearson correlation.

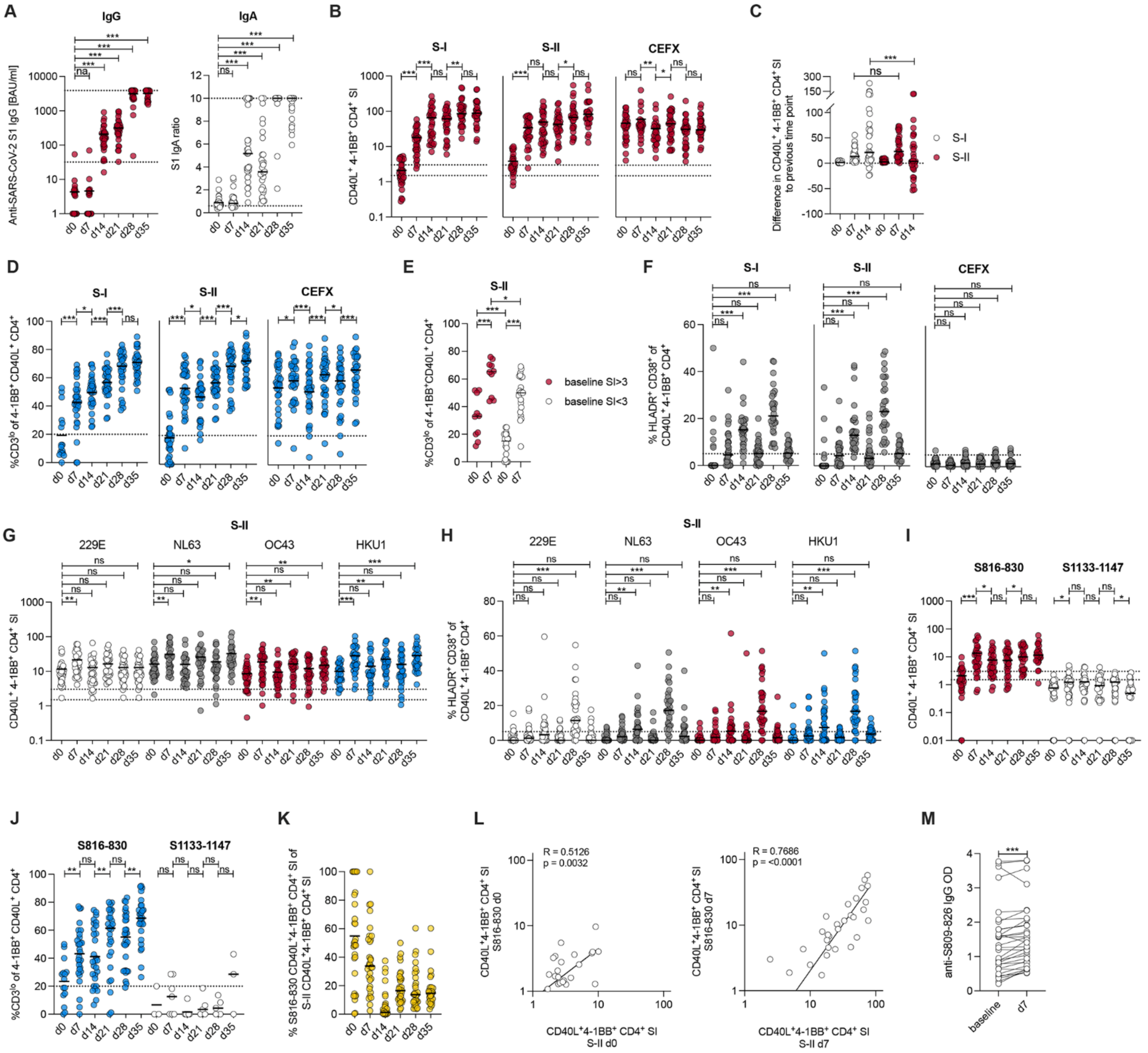
A
IgG
IgA
Anti-SARS-CoV-2 S1 IgG [BAU/ml]
S1 IgA ratio
B
S-I
S-II
CEFX
CD40L+ 4-1BB+ CD4+ SI
C
Difference in CD40L+ 4-1BB+ CD4+ SI to previous time point
S-I
S-II
D
S-I
S-II
CEFX
%CD3lo of 4-1BB+ CD40L+ CD4+
E
S-II
%CD3lo of 4-1BB+CD40L+ CD4+
baseline SI>3
baseline SI<3
F
S-I
S-II
CEFX
% HLADR+ CD38+ of CD40L+ 4-1BB+ CD4+
G
S-II
229E
NL63
OC43
HKU1
CD40L+ 4-1BB+ CD4+ SI
H
S-II
229E
NL63
OC43
HKU1
% HLADR+ CD38+ of CD40L+ 4-1BB+ CD4+
I
S816-830
S1133-1147
CD40L+ 4-1BB+ CD4+ SI
J
S816-830
S1133-1147
%CD3lo of 4-1BB+ CD40L+ CD4+
K
% S816-830 CD40L+4-1BB+ CD4+ SI of S-II CD40L+4-1BB+ CD4+ SI
L
R = 0.5126
p = 0.0032
CD40L+4-1BB+ CD4+ SI S816-830 d0
CD40L+4-1BB+ CD4+ SI S-II d0
R = 0.7686
p = <0.0001
CD40L+4-1BB+ CD4+ SI S816-830 d7
CD40L+4-1BB+ CD4+ SI S-II d7
M
anti-S809-826 IgG OD
baseline
d7

Fig. 6. HCoV-specific SARS-CoV-2-cross-reactive T cells are recruited into the BNT162b2 vaccine response. **(A)** Serum anti-SARS-CoV-2 S1 IgG binding antibody units (BAU) and IgA titer ratio were determined at baseline, d7, and d14 after primary vaccination with BNT162b, immediately before secondary vaccination (d21) as well as 1 (d28) and 2 weeks (d35) after secondary vaccination. All values below 1 were set to 1. The lower and upper cut-off levels for IgG were set at 32 and 3900, respectively, the corresponding IgA cut-offs at 0.6 and 10, respectively, indicated by dotted lines. **(B)** Plots show the SI of CD40L$^+$4-1BB$^+$ CD4$^+$ T cells after stimulation with S-I, S-II, and CEFX at baseline and indicated time points. **(C)** Difference in SI after stimulation with S-I and S-II at each time point relative to the previous time point. **(D)** Plots show the frequencies of CD3^{lo} of CD40L$^+$4-1BB$^+$ CD4$^+$ T cells after stimulation with S-I, S-II, and CEFX for responses with an SI≥1.5. **(E)** Frequencies of CD3^{lo} of CD40L$^+$4-1BB$^+$ CD4$^+$ T cells at d0 and d7 in cross-reactive donors (baseline SI≥3, red circles) and non-cross-reactive donors (baseline SI<3, white circles). **(F)** Frequencies of HLADR$^+$CD38$^+$ cells among CD40L$^+$4-1BB$^+$ CD4$^+$ T cells after stimulation with S-I, S-II, and CEFX at the indicated time points. **(G)** SI of CD40L$^+$4-1BB$^+$ CD4$^+$ T cells and **(H)** frequencies of HLADR$^+$CD38$^+$ among these cells after stimulation with HCoV S-II peptide pools at baseline and indicated time points. **(I)** SI of CD40L$^+$4-1BB$^+$ CD4$^+$ T cells and **(J)** frequencies of CD3^{lo} events (SI≥1.5) among these cells after stimulation with peptide S816-830 and control peptide S1133-1147 at baseline and indicated time points. **(K)** Proportion of S816-830-reactive T cells over SARS-CoV-2 S-II-reactive T cells. **(L)** The relationship between responses to S816-830 and SARS-CoV-2 S-II peptide pool stimulation at d0 (left) and d7 (right). **(M)** OD of anti-S809-826-peptide IgG ELISA from sera before and 7 days after primary vaccination. A, F, G, H, I, J: **P*<0.05, ***P*<0.01, ****P*<0.001 and ns for *P*>0.05 (repeated measure one-way-ANOVA with Dunnett`s correction). B, C, D, M: **P*<0.05, ***P*<0.01, ****P*<0.001 and ns for *P*>0.05 (paired Student's *t* test). E: **P*<0.05, ***P*<0.01, ****P*<0.001 and ns for *P*>0.05 (Student's *t* test). L: Pearson correlation.
