# Supplementary material for: Cross-reactive CD4+ T cells enhance SARS-CoV-2 immune responses upon infection and vaccination
Source: Science. 2021 Aug 31;374(6564):eabh1823. doi: 10.1126/science.abh1823 (PMC10026850; doi:10.1126/science.abh1823)
Supplement: Supplementary file 2 — Figs. S1 to S6 Tables S1 to S3 [file science.abh1823_sm.pdf]

Science AAAS

Supplementary Materials for

Cross-reactive CD4$^+$ T cells enhance SARS-CoV-2 immune responses upon infection and vaccination

Lucie Loyal *et al.*

Corresponding authors: Andreas Thiel, andreas.thiel@charite.de; Claudia Giesecke-Thiel, giesecke@molgen.mpg.de

Science **374**, eabh1823 (2021)
DOI: 10.1126/science.abh1823

The PDF file includes:

Figs. S1 to S6
Tables S1 to S3

Other Supplementary Material for this manuscript includes the following:

MDAR Reproducibility Checklist

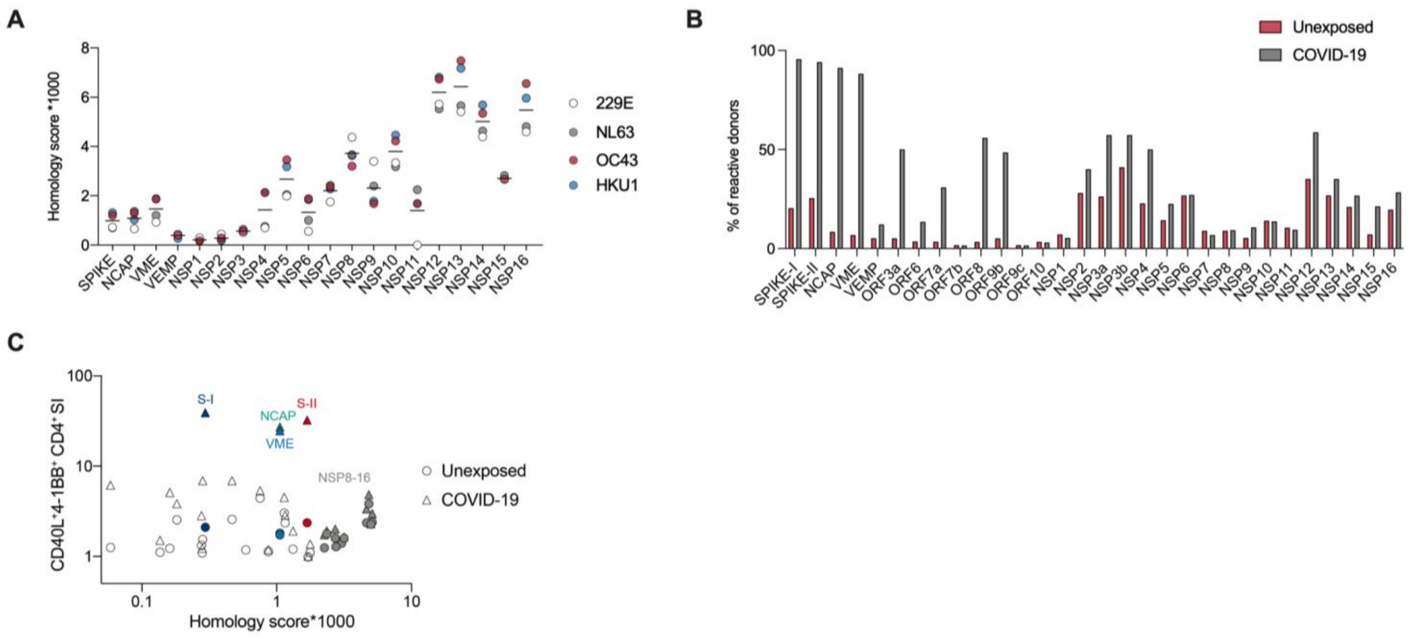

Fig. S1. HCoV homology scores and SARS-CoV-2 peptide pool reactivity **(A)** Similarity between SARS-CoV-2 and the different endemic coronaviruses 229E, NL63, OC43, and HKU1 (isolate N1). Scores were calculated using a PAM30 substitution matrix for all 9-mers of the respective antigen against all 9-mers of the proteome of SARS-CoV-2. The homology score is the percentage of 9-mer pairs with a score above 30. **(B)** Frequency of donors with T cells reactive against the indicated peptide pools in unexposed and COVID-19 convalescents from Fig. 1A with an SI≥3. **(C)** To visualize the probability of homologous epitope recognition, a homology score for the comparison of a SARS-CoV-2 antigen with all four endemic coronaviruses 229E, NL63, OC43, and HKU1 (isolates N1, N2 N5) was plotted against the mean SI of 60 unexposed donors (circles) and 59 COVID-19 convalescents (triangles). For the calculation of the homology score all theoretical possible 9 aa fragments in each peptide of a respective PepMix were scored against all possible 9 aa fragments of the complete proteome of all other HCoVs using a PAM30 substitution matrix. The homology score is the percentage of individual comparisons with a score > 30.

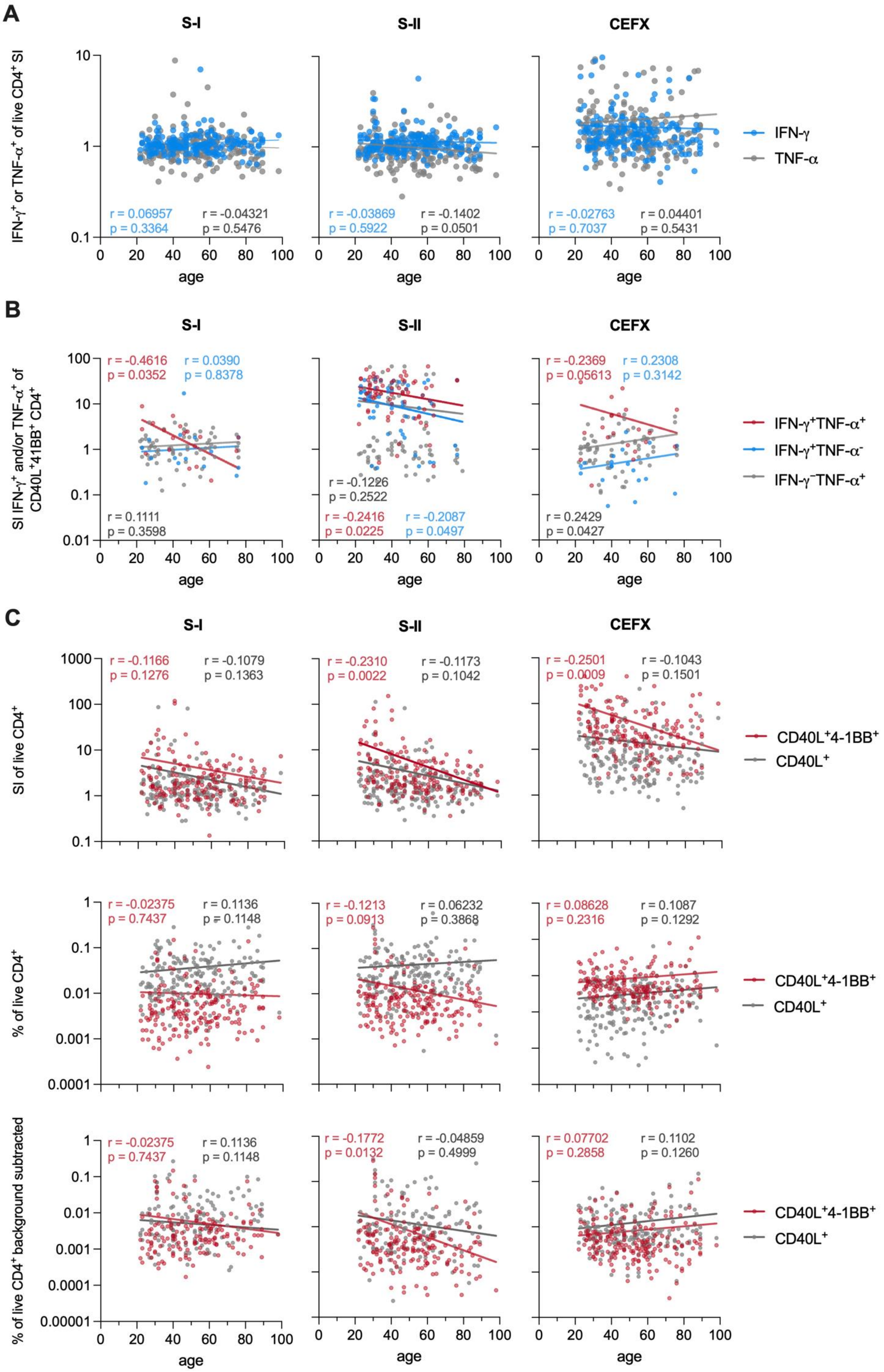
A
S-I
S-II
CEFX
IFN-γ+ or TNF-α+ of live CD4+ SI
IFN-γ
TNF-α
r = 0.06957
p = 0.3364
r = -0.04321
p = 0.5476
r = -0.03869
p = 0.5922
r = -0.1402
p = 0.0501
r = -0.02763
p = 0.7037
r = 0.04401
p = 0.5431
age
B
SI IFN-γ+ and/or TNF-α+ of CD40L+41BB+ CD4+
IFN-γ+TNF-α+
IFN-γ+TNF-α-
IFN-γ-TNF-α+
r = -0.4616
p = 0.0352
r = 0.0390
p = 0.8378
r = 0.1111
p = 0.3598
r = -0.1226
p = 0.2522
r = -0.2416
p = 0.0225
r = -0.2087
p = 0.0497
r = -0.2369
p = 0.05613
r = 0.2308
p = 0.3142
r = 0.2429
p = 0.0427
C
SI of live CD4+
CD40L+4-1BB+
CD40L+
r = -0.1166
p = 0.1276
r = -0.1079
p = 0.1363
r = -0.2310
p = 0.0022
r = -0.1173
p = 0.1042
r = -0.2501
p = 0.0009
r = -0.1043
p = 0.1501
% of live CD4+
r = -0.02375
p = 0.7437
r = 0.1136
p = 0.1148
r = -0.1213
p = 0.0913
r = 0.06232
p = 0.3868
r = 0.08628
p = 0.2316
r = 0.1087
p = 0.1292
% of live CD4+ background subtracted
r = -0.02375
p = 0.7437
r = 0.1136
p = 0.1148
r = -0.1772
p = 0.0132
r = -0.04859
p = 0.4999
r = 0.07702
p = 0.2858
r = 0.1102
p = 0.1260

Fig. S2. Cytokine characterization and activation molecule profiling of spike-specific $CD4^+$ T cells by participant age

(A, B) SI of IFN-γ^+ or TNF-α^+ of live $CD4^+$ T cells by age (A) and (B) SI of IFN-γ^+TNF-α^-, IFN-γ^-TNF-α^+ and IFN-γ^+TNF-α^+ $CD40L^+41BB^+$ $CD4^+$ T cells after stimulation of PBMCs with SARS-CoV-2 S-I, SARS-CoV-2 S-II, or CEFX in n=568 unexposed donors and n=174 COVID-19 convalescents. **(C)** SI of live $CD4^+$ T cells (upper row), frequencies of live $CD4^+$ T cells (middle row), and background (unstimulated control)-subtracted frequencies of live $CD4^+$ T cells (lower row) stimulated with S-I, S-II or CEFX and gated for $CD40L^+$4-1BB^+ or $CD40L^+$ expression.

A, B, C: Pearson correlation.

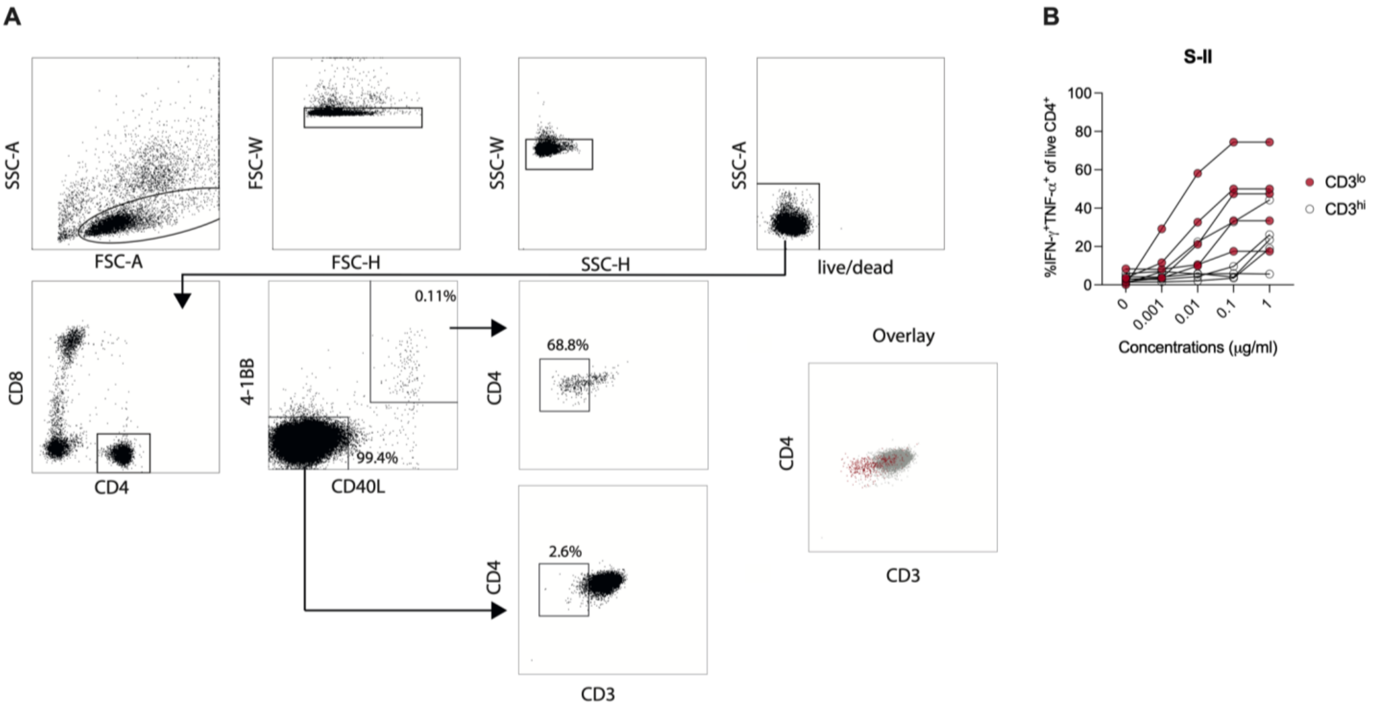

Fig. S3. CD3^{lo} gating in flow cytometry displays functional avidity.
(A) Representative gating strategy for CD3^{lo} cells within non-reactive CD4^{+} T cells and S-II-reactive CD40L^{+}4-1BB^{+} CD4^{+} T cells. Cells were pre-gated on live, doublet-free CD4^{+} lymphocytes. **(B)** S-II-reactive CD3^{lo} and CD3^{hi} CD40L^{+}41BB^{+} CD4^{+} T cells were sorted and expanded, respectively, and restimulated with indicated concentrations of S-II and the frequencies of IFN-γ^{+}TNF-α^{+} CD4^{+} T cells measured.

A

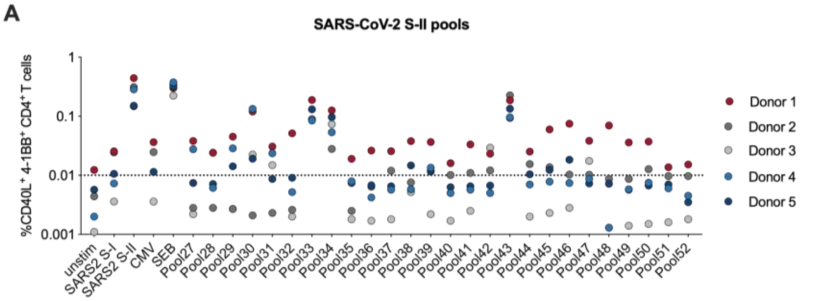

D

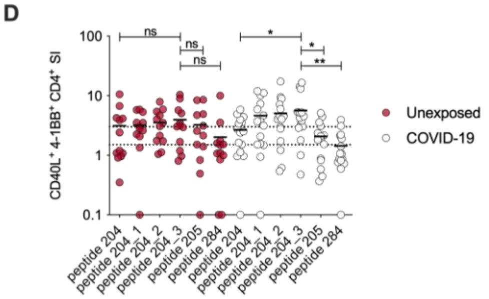

B

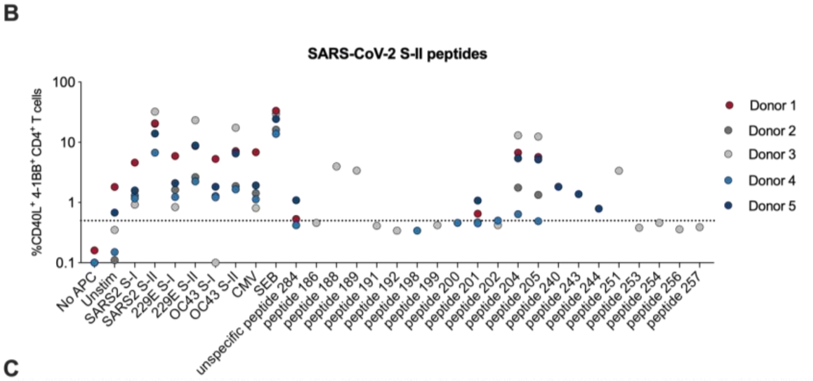

C

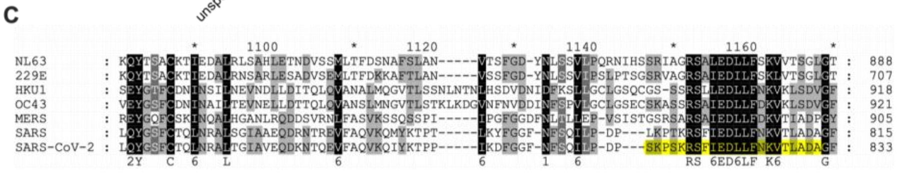

E

allele HLA-DPA1*01:03/	length	peptide	adjusted rank
DPB1*04:01	14	SFIEDLLFNKVTLA	0,8
DPB1*04:01	13	SFIEDLLFNKVTL	0,83
DPB1*04:01	11	SFIEDLLFNKV	0,97
DPB1*04:01	15	SFIEDLLFNKVTLAD	1,2
DPB1*04:02	14	SFIEDLLFNKVTLA	1,29
DPB1*04:02	13	SFIEDLLFNKVTL	1,37
DPB1*04:01	12	SFIEDLLFNKVT	1,6
DPB1*04:02	15	SFIEDLLFNKVTLAD	1,8
DPB1*04:02	11	SFIEDLLFNKV	1,83
DPB1*04:01	13	FIEDLLFNKVTLA	2,34
DPB1*04:02	12	SFIEDLLFNKVT	2,4
DPB1*04:01	14	FIEDLLFNKVTLAD	2,58
DPB1*04:01	12	FIEDLLFNKVTL	2,91
DPB1*04:02	13	FIEDLLFNKVTLA	3,74
DPB1*04:02	14	FIEDLLFNKVTLAD	3,88
DPB1*04:01	11	FIEDLLFNKVT	4,4
DPB1*04:02	12	FIEDLLFNKVTL	5,34
DPB1*04:02	11	FIEDLLFNKVT	7,43
DPB1*02:01	14	SFIEDLLFNKVTLA	8,18
DPB1*02:01	15	SFIEDLLFNKVTLAD	9,2
DPB1*02:01	13	SFIEDLLFNKVTL	9,82
DPB1*04:01	13	IEDLLFNKVTLAD	11,54
DPB1*02:01	12	SFIEDLLFNKVT	14,53
DPB1*02:01	14	FIEDLLFNKVTLAD	15,08
DPB1*04:01	12	IEDLLFNKVTLA	15,12
DPB1*04:02	13	IEDLLFNKVTLAD	15,28
DPB1*02:01	11	SFIEDLLFNKV	16
DPB1*02:01	13	FIEDLLFNKVTLA	18,71
DPB1*02:01	13	IEDLLFNKVTLAD	18,71
DPB1*04:02	12	IEDLLFNKVTLA	19,57

F

229E

allele DPA1*01:03/	length	peptide	adjusted rank
DPB1*04:02	14	SAIEDILFSKLVTS	2,58
DPB1*04:01	14	SAIEDILFSKLVTS	2,69
DPB1*04:02	15	SAIEDILFSKLVTSG	3,4
DPB1*04:01	13	SAIEDILFSKLVT	3,59
DPB1*04:01	15	SAIEDILFSKLVTSG	3,8
DPB1*04:02	13	SAIEDILFSKLVT	4,21
DPB1*04:01	13	AIEDILFSKLVTS	4,68
DPB1*04:01	14	AIEDILFSKLVTSG	4,74
DPB1*04:02	14	AIEDILFSKLVTSG	4,74
DPB1*04:01	12	SAIEDILFSKLV	4,74
DPB1*02:01	14	SAIEDILFSKLVTS	4,85
DPB1*04:02	13	AIEDILFSKLVTS	4,99
DPB1*02:01	15	SAIEDILFSKLVTSG	5,9
DPB1*04:01	12	AIEDILFSKLVT	6,52
DPB1*04:02	12	SAIEDILFSKLV	6,52
DPB1*02:01	13	SAIEDILFSKLVT	6,55
DPB1*04:01	11	AIEDILFSKLV	8
DPB1*04:02	12	AIEDILFSKLVT	8,9
DPB1*02:01	14	AIEDILFSKLVTSG	9,05
DPB1*04:01	11	SAIEDILFSKL	9,14
DPB1*02:01	12	SAIEDILFSKLV	9,19
DPB1*04:02	13	IEDILFSKLVTSG	9,51
DPB1*04:01	13	IEDILFSKLVTSG	9,67
DPB1*02:01	13	AIEDILFSKLVTS	9,98
DPB1*04:01	12	IEDILFSKLVTS	10,97
DPB1*02:01	13	IEDILFSKLVTSG	11,7
DPB1*04:02	12	IEDILFSKLVTS	11,86
DPB1*04:02	11	AIEDILFSKLV	12,57
DPB1*02:01	11	SAIEDILFSKL	14,28
DPB1*04:02	11	SAIEDILFSKL	14,28
DPB1*02:01	12	IEDILFSKLVTS	14,83
DPB1*04:01	11	IEDILFSKLVT	16,57

NL63

allele DPA1*01:03/	length	peptide	adjusted rank
DPB1*04:01	14	SALEDLLFSKVVTS	3,77
DPB1*04:02	14	SALEDLLFSKVVTS	4,2
DPB1*04:01	13	SALEDLLFSKVVT	4,21
DPB1*04:01	15	SALEDLLFSKVVTSG	5,1
DPB1*04:02	15	SALEDLLFSKVVTSG	5,4
DPB1*04:02	13	SALEDLLFSKVVT	5,46
DPB1*04:01	12	SALEDLLFSKVV	5,63
DPB1*04:01	13	ALEDLLFSKVVTS	7,02
DPB1*04:01	14	ALEDLLFSKVVTSG	7,11
DPB1*04:02	12	SALEDLLFSKVV	7,41
DPB1*04:02	14	ALEDLLFSKVVTSG	7,86
DPB1*04:02	13	ALEDLLFSKVVTS	8,11
DPB1*02:01	14	SALEDLLFSKVVTS	8,83
DPB1*04:01	12	ALEDLLFSKVVT	9,49
DPB1*02:01	13	SALEDLLFSKVVT	10,14
DPB1*04:01	11	ALEDLLFSKVV	10,28
DPB1*04:01	11	SALEDLLFSKV	10,28
DPB1*02:01	15	SALEDLLFSKVVTSG	11
DPB1*04:02	12	ALEDLLFSKVVT	13,94
DPB1*02:01	12	SALEDLLFSKVV	14,23
DPB1*02:01	14	ALEDLLFSKVVTSG	15,08
DPB1*04:02	11	ALEDLLFSKVV	17,14
DPB1*02:01	13	ALEDLLFSKVVTS	17,15
DPB1*04:01	13	LEDLLFSKVVTSG	17,15
DPB1*04:02	11	SALEDLLFSKV	17,71
DPB1*04:02	13	LEDLLFSKVVTSG	18,71
DPB1*04:01	12	LEDLLFSKVVTS	19,57

OC43

allele DPA1*01:03/	length	peptide	adjusted rank
DPB1*04:01	14	SAIEDLLFDKVKLS	6,25
DPB1*04:01	13	SAIEDLLFDKVKL	7,8
DPB1*04:01	15	SAIEDLLFDKVKLSD	8,7
DPB1*04:01	13	AIEDLLFDKVKLS	9,51
DPB1*04:01	14	AIEDLLFDKVKLSD	9,58
DPB1*04:02	14	SAIEDLLFDKVKLS	10,01
DPB1*04:02	13	SAIEDLLFDKVKL	12,79
DPB1*04:02	14	AIEDLLFDKVKLSD	14
DPB1*04:02	15	SAIEDLLFDKVKLSD	14
DPB1*04:02	13	AIEDLLFDKVKLS	14,66
DPB1*02:01	15	SAIEDLLFDKVKLSD	15
DPB1*02:01	14	SAIEDLLFDKVKLS	15,08
DPB1*04:01	12	AIEDLLFDKVKL	15,72
DPB1*04:01	13	IEDLLFDKVKLSD	17,15
DPB1*04:01	12	SAIEDLLFDKVK	18,68
DPB1*02:01	13	SAIEDLLFDKVKL	18,71

HKU1

allele DPA1*01:03/	length	peptide	adjusted rank
DPB1*04:01	13	SLLEDLLFNKVKL	2,03
DPB1*04:01	14	SLLEDLLFNKVKLS	2,05
DPB1*04:02	14	SLLEDLLFNKVKLS	2,91
DPB1*04:01	15	SLLEDLLFNKVKLSD	3
DPB1*04:02	13	SLLEDLLFNKVKL	3,12
DPB1*04:02	15	SLLEDLLFNKVKLSD	4
DPB1*04:01	13	LLEDLLFNKVKLS	4,05
DPB1*04:01	12	SLLEDLLFNKVK	4,15
DPB1*04:01	14	LLEDLLFNKVKLSD	4,2
DPB1*04:01	11	SLLEDLLFNKV	4,23
DPB1*04:01	12	LLEDLLFNKVKL	5,04
DPB1*04:02	13	LLEDLLFNKVKLS	5,93
DPB1*04:02	14	LLEDLLFNKVKLSD	6,14
DPB1*04:02	12	SLLEDLLFNKVK	6,23
DPB1*04:02	11	SLLEDLLFNKV	7,43
DPB1*02:01	14	SLLEDLLFNKVKLS	7,65
DPB1*04:02	12	LLEDLLFNKVKL	7,71
DPB1*02:01	15	SLLEDLLFNKVKLSD	8,1
DPB1*02:01	13	SLLEDLLFNKVKL	8,58
DPB1*04:01	11	LLEDLLFNKVK	9,14
DPB1*04:01	13	LEDLLFNKVKLSD	11,38
DPB1*02:01	14	LLEDLLFNKVKLSD	14
DPB1*04:01	12	LEDLLFNKVKLS	14,53
DPB1*04:02	11	LLEDLLFNKVK	14,85
DPB1*02:01	13	LEDLLFNKVKLSD	17,15
DPB1*04:02	13	LEDLLFNKVKLSD	17,15
DPB1*02:01	13	LLEDLLFNKVKLS	18,71

Fig. S4. Identification of S816-830 by narrowing down the sequence regions mapped in SARS-CoV-2 peptide pools that exhibited highest T cell cross-reactivity.

(A) Expanded SARS-CoV-2 S-I- or S-II specific CD40L$^+$4-1BB$^+$ CD4$^+$ short-term-culture T cells were restimulated with different matrix pools in the presence autologous feeder cells and the frequency of CD40L$^+$4-1BB$^+$ reactive cells determined. The cut-off is set at 0.01 based on unstimulated controls. **(B)** Expanded SARS-CoV-2 S-I- or S-II reactive CD40L$^+$4-1BB$^+$ CD4$^+$ T cells were restimulated with single peptide candidates determined from matrix stimulation (shown in A) in the presence of autologous feeder cells and the frequency CD40L$^+$4-1BB$^+$ reactive CD4$^+$ T cells was determined. The cut-off is set at 0.5 based on unstimulated controls. **(C)** Position of single peptides 204 and 205 (yellow) in sequence overlay of the known 7 human coronaviruses. Color code indicates homology: black: identical amino acid; gray: conservative amino acid replacement. **(D)** Identification of the dominant peptide at the 204 to 205 sequence intersection. The number behind the underline character indicates the number of amino acids by which the 15-mer was shifted towards the C-terminus beginning with the sequence of 204 and ending with the sequence of 205. A representative selection of donors who displayed an SI≥3 in one or more stimulations is shown. **(E, F)** MHC-II HLA binding prediction of HLA-DPA1*01:03/DPB1*02:01, HLA-DPA1*01:03/DPB1*04:01 and HLA-DPA1*01:03/DPB1*04:02 with S816-830 and derived fragments **(E)** or HCoV-derived homologous peptides and fragments thereof **(F)**.

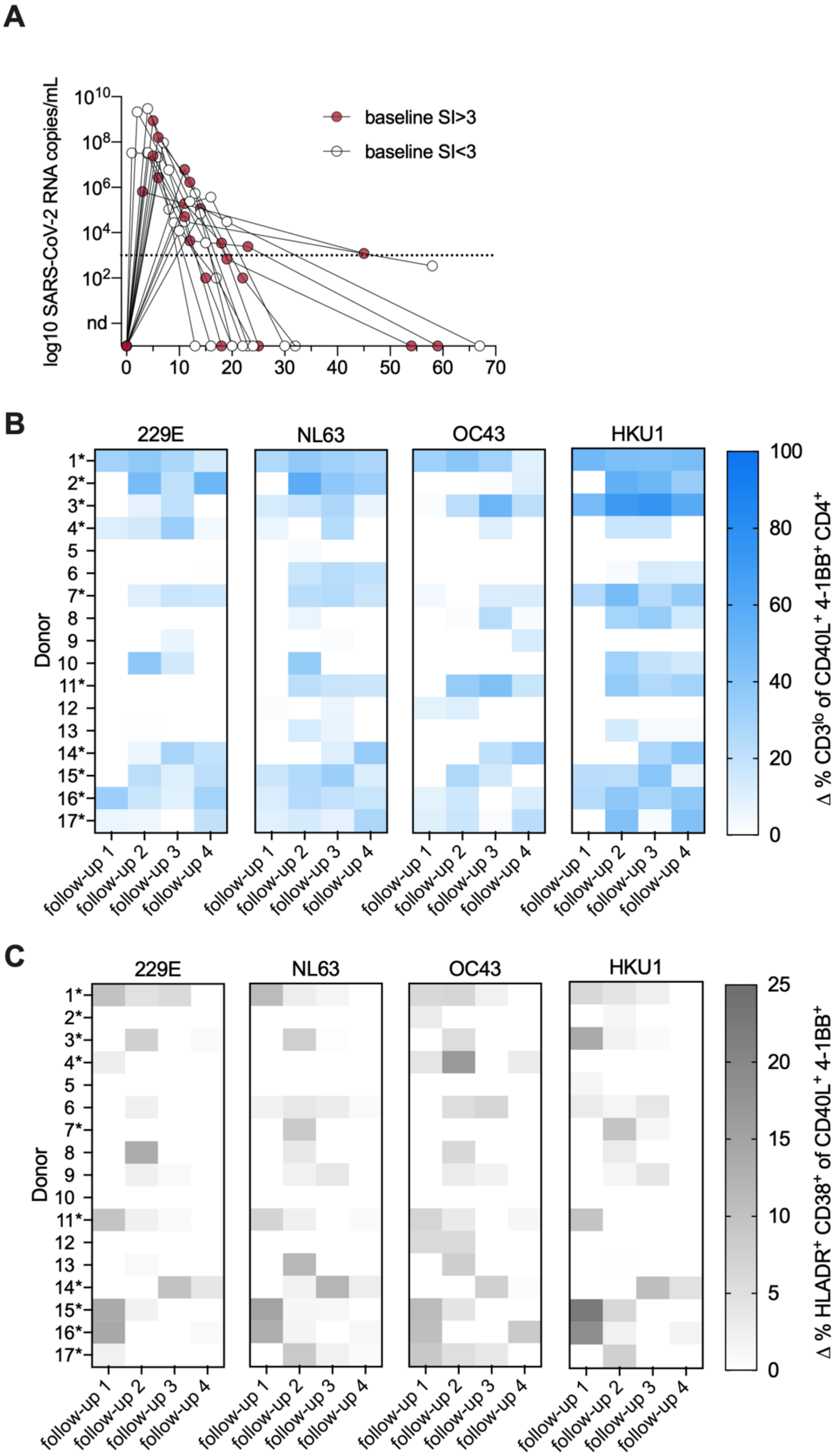
A
log10 SARS-CoV-2 RNA copies/mL
10^10
10^8
10^6
10^4
10^2
nd
0
10
20
30
40
50
60
70
baseline SI>3
baseline SI<3
B
229E
NL63
OC43
HKU1
Donor
1*
2*
3*
4*
5
6
7*
8
9
10
11*
12
13
14*
15*
16*
17*
follow-up 1
follow-up 2
follow-up 3
follow-up 4
Δ % CD3lo of CD40L+ 4-1BB+ CD4+
100
80
60
40
20
0
C
229E
NL63
OC43
HKU1
Donor
follow-up 1
follow-up 2
follow-up 3
follow-up 4
Δ % HLADR+ CD38+ of CD40L+ 4-1BB+
25
20
15
10
5
0

Fig. S5. SARS-CoV-2 RNA concentration kinetics and HCoV-reactive T cell characteristics upon SARS-CoV- infection.

(A) Viral RNA copies per mL of diluted swab material at indicated time points. The dotted line indicates the limit of quantification (nd = not detected). Donors with an SI≥3 for SARS-CoV-2 S-II at baseline are represented as red circles, individuals with an SI<3 are shown as white circles. **(B, C)** Heatmaps show the delta (Δ) frequency of $CD3^{lo}$ **(B)** and the Δ frequency of $HLADR^{+}CD38^{+}$ cells **(C)** among $CD40L^{+}4\text{-}1BB^{+}$ $CD4^{+}$ T cells after stimulation with S-II pools of the indicated HCoVs. Δ represents the change of the parameter at the given time point relative to baseline (i.e., white depicts no increase). "*" identifies S816-830 peptide responders.

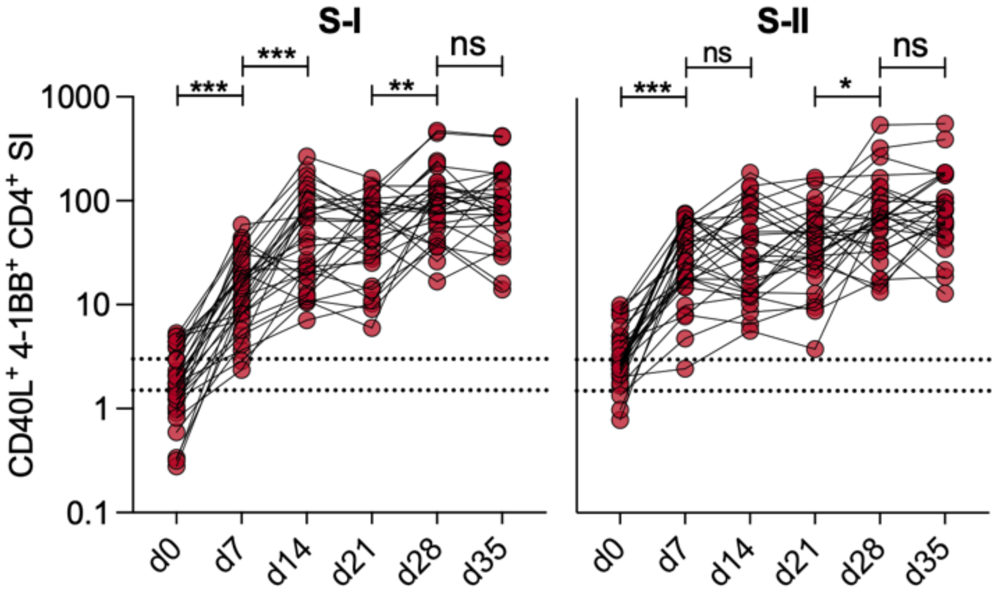

Fig. S6. Individual T cell responses to vaccination.

Response kinetics of CD40L$^+$4-1BB$^+$ CD4$^+$ T cells in individual vaccinees upon stimulation with SARS-CoV-2 S-I or S-II, as shown in Fig. 6B. **P*<0.05, ***P*<0.01, ****P*<0.001, and ns for *P*>0.05 (paired Student's *t* test). Secondary vaccination occurred on day 21.

Table S1. Donor characteristics.

Cohort	Subcohort	Number donors	Age mean (St.d.)	Age range	Gender (f/m)	ICU	Hospitalization	Mild/ asymptomatic	Measurement day p.s.o. mean (St.d.)	Measurement day p.s.o. range	Figure(s)
**Orfeome**											Fig. 1, S1
	COVID-19 convalescents	59	50.6 (15.0)	21-79	24/35	20	17	22	152 (57)	17-262	
	Unexposed	60	45.7 (16.1)	25-76	36/24	-	-	-	-	-	
**Basic CCC**											Fig. 2, 3, S3
	COVID-19 WHO 1-2	133	43.8 (13.6)	18-78	85/48	-	-	133	128 (74)	21-304	
	COVID-19 WHO 3-4	19	53.1 (12.8)	31-75	7/12	-	19	-	137 (41)	50-214	
	COVID-19 WHO 5-7	22	59.2 (13.3)	27-79	6/16	22	-	-	143 (59)	61-229	
	Unexposed	568	47.5 (11.8)	18-98	439/129	-	-	-	-	-	
**Other pathogen pools**											Fig. 2
	Young (<30)	20	23.5 (2.4)	20-29	11/9	-	-	-	-	-	
	Old (>65)	19	70.4 (3.8)	65-76	11/8	-	-	-	-	-	
**Follow-up**											Fig. 5
	COVID-19 convalescents	17	39.2 (10.8)	22-58	11/6			17	see Table 2	see Table 2	
**Vaccination**											
	Volunteers	31	40.9 (11.5)	20-67	12/17	-	-	-	-	-	Fig. 6, S5

p.s.o.=post symptom onset

Table S2. Characteristics of the follow-up donors.

Donor	Gender	Age	BMI (kg/m^2)	Symptoms	Comorbidities	BL %CD4$^+$	BL S-I SI	BL S-II SI	day BL	day FU1	day FU2	day FU 3	day FU4
**A**	m	30	22.9	Fatigue. headache. cough. fever. nausea	Allergies. asthma	48.0	1.1	5.0	-76	6	12	19	54
**B**	f	47	33.2	Cough. loss of taste and smell. body ace	Allergies	53.8	0.5	1.6	-110	-	15	22	29
**C**	m	31	25.5	Fatigue. headache. cough. fever. loss of taste and smell. dermatological symptoms. body ace	-	47.8	3.1	1.9	-32	8	15	22	35
**D**	f	31	34.8	Fatigue. headache. cough. fever. loss of taste and smell. nausea. diarrhea. body ache	Allergies. migraine. bronchial hyperreactivity	55.6	2.0	2.1	-39	4	12	19	67
**E**	f	40	32	Fatigue. headache. cough. fever. loss of taste and smell. nausea. diarrhea. dermatological symptoms	Allergies. asthma. migraine. Hashimoto-Thyroiditis	61.1	1.6	1.3	-64	7	13	20	48
**F**	f	47	33.1	Cough. fever. nausea. diarrhea	-	73.4	3.5	6.4	-73	5	11	18	54
**G**	f	31	31.1	Fatigue. headache. cough. fever. body ace	Acne vulgaris. pseudotumor cerebri	66.0	1.1	1.3	-82	4	11	18	58
**H**	f	31	27.1	Fatigue. headache. loss of taste and smell. body ace	Thyroidectomy	67.0	2.9	0.8	-38	-	10	24	53
**I**	f	22	28.5	Fatigue. headache. cough. fever. loss of taste and smell. nausea. diarrhea. dermatological symptoms. body ace	Gastritis	60.3	2.5	5.9	-37	-	11	25	54
**J**	f	37	20.3	Fatigue. headache. cough. loss of taste and smell. nausea	Allergies	56.4	1.0	1.4	-19	-	14	23	71
**K**	m	52	29.2	Fatigue. headache. cough. fever. body ace	Allergies	66.8	4.0	5.0	-39	-	11	22	58
**L**	f	55	37.7	Fatigue. headache. cough. fever. loss of taste and smell. nausea. dermatological symptoms. body ace	Allergies. asthma. hypertonia	78.8	2.3	2.7	-13	9	16	23	57
**M**	m	58	24.2	Headache. fever. loss of taste and smell	Cardiac arrhythmia	56.9	0.3	3.0	-82	5	12	23	56
**N**	m	53	23.5	Fatigue. headache. cough. loss of taste and smell. body ace	-	66.4	4.3	5.8	-56	3	14	-	45
**O**	f	40	22.1	Fatigue. headache. fever. diarrhea. dermatological symptoms. body ace	-	59.7	1.2	2.3	-84	6	13	20	42
**P**	f	31	21.1	Fatigue. headache. loss of smell. dermatological symptoms. body ace	-	61.4	3.3	3.8	-111	7	15	20	42
**Q**	m	31	24.2	Loss of taste and smell	Arterial hypertonia	53.0	1.0	2.0	-68	8	16	30	66

BL=baseline, FU=follow-up. None of the donors required hospitalization.

Table S3. List of antibodies used for flow cytometric analyses.

Marker	Fluorochrome	Clone	Manufacturer	RRID	Titration
**CD3**	FITC	REA613	Miltenyi	AB_2725966	1:50
**CD4**	VioGreen	REA623	Miltenyi	AB_2726041	1:50
**CD8**	VioBlue	REA734	Miltenyi	AB_2659239	1:50
**HLA-DR**	PerCpVio700	REA805	Miltenyi	AB_2652172	1:50
**CD38**	APC	REA671	Miltenyi	AB_2726164	1:100
**CD40L**	PEVio770	REA238	Miltenyi	AB_2751209	1:50
**4-1BB**	PE	REA765	Miltenyi	AB_2654986	1:50
**IFN-g**	A700	B27	Biolegend	AB_961351	1:100
**TNF-a**	BV605	MAb11	Biolegend	AB_11203719	1:100
